# Fluconazole resistance in *Candida albicans* is induced by *Pseudomonas aeruginosa* quorum sensing

**DOI:** 10.1038/s41598-020-64761-3

**Published:** 2020-05-08

**Authors:** H. M. H. N. Bandara, D. L. A. Wood, I. Vanwonterghem, P. Hugenholtz, B. P. K. Cheung, L. P. Samaranayake

**Affiliations:** 1 Oral Microbiology, Bristol Dental School, University of Bristol, Lower Maudlin Street, Bristol, BS1 2LY UK; 2 Australian Centre for Ecogenomics, School of Chemistry and Molecular Biosciences, The University of Queensland, St Lucia, QLD 4072 Australia; 3 Faculty of Dentistry, The University of Hong Kong, 34 Hospital Rd, Sai Ying Pun, Hong Kong SAR, China; 4 College of Dental Medicine, The University of Sharjah, P.O. Box, 27272 Sharjah, UAE

**Keywords:** Fungi, Microbiology, Antifungal agents, Antimicrobial resistance

## Abstract

Microorganisms employ quorum sensing (QS) mechanisms to communicate with each other within microbial ecosystems. Emerging evidence suggests that intraspecies and interspecies QS plays an important role in antimicrobial resistance in microbial communities. However, the relationship between interkingdom QS and antimicrobial resistance is largely unknown. Here, we demonstrate that interkingdom QS interactions between a bacterium, *Pseudomonas aeruginosa* and a yeast, *Candida albicans*, induce the resistance of the latter to a widely used antifungal fluconazole. Phenotypic, transcriptomic, and proteomic analyses reveal that *P. aeruginosa’s* main QS molecule, N-(3-Oxododecanoyl)-L-homoserine lactone, induces candidal resistance to fluconazole by reversing the antifungal’s effect on the ergosterol biosynthesis pathway. Accessory resistance mechanisms including upregulation of *C. albicans* drug-efflux, regulation of oxidative stress response, and maintenance of cell membrane integrity, further confirm this phenomenon. These findings demonstrate that *P. aeruginosa* QS molecules may confer protection to neighboring yeasts against azoles, in turn strengthening their co-existence in hostile polymicrobial infection sites.

## Introduction

Microbial communities residing within the human body, either transiently or permanently, play a pivotal role in human health and disease^1^. In particular, interkingdom polymicrobial infections due to pathogenic fungi and bacteria are relatively common and are seen in the oral cavity, respiratory tract, gastrointestinal system, skin, and urinary tract^1,2^. For instance, the focal fungal pathogen of our study, *Candida albicans*, contributes to >50% of the total microbial burden in mixed fungal-bacterial chronic wound infections, and has been frequently co-isolated with bacterial pathogens including *Pseudomonas aeruginosa* and *Staphylococcus aureus*^3–5^. *C. albicans* is considered an independent risk factor for ventilator associated pneumonia and co-exists with *P. aeruginosa* in 26% of these infections^6^. When superinfected with *Candida*, the prognosis of *P. aeruginosa* infections in cystic fibrosis lungs are significantly poorer compared to the bacterial infection alone^7,8^. Alarmingly, 27–56% of nosocomial *C. albicans* blood stream infections are associated with *Staphylococcus epidermidis, S. aureus* and *Enterococcus* species^9^. Moreover, *Candida spp*. are co-isolated with vaginal streptococci in 20–34% of recurrent vulvovaginal candidiasis, and with oral streptococci in 50–75% of denture stomatitis cases^10–12^. Candidal-bacterial polymicrobial infections are responsible for a high incidence of mortality and morbidity due to their increased dissemination, antimicrobial resistance, and the lack of sensitive diagnostics^9,13^. Hence, fungal-bacterial interkingdom infections represent an, as yet, understudied health issue warranting further investigation.

The severity and outcome of polymicrobial infections are dictated not only by the nature and the composition of the constituent microbiota, but also by the chemical communications between co-habitants. Quorum sensing (QS) is a universal chemical messenger system used by microorganisms to interact with each other. QS is defined as a cell-density dependent, coordinated gene expression in microbial communities in response to threshold concentrations of specific chemical signalling molecules (quorum sensing molecules; QSMs) leading to a synchronized population response^14^. QS is essential for microbes to optimize their survival in dynamic, constantly challenging niches, as the chemical messengers help correlate individual cellular functions to microbial community-based requirements^14^. These include regulation of biofilm development and maturation, motility and virulence, bacterial sporulation, formation of fungal fruiting bodies, conjugal plasmid transfer and antimicrobial resistance, and antibiotic synthesis^15–17^. QS interactions can occur among microbes from the same species (intraspecies QS), different species (interspecies QS) or even between members of different kingdoms (interkingdom QS)^14^. However, most studies have focused on intra- and interspecies QS, and our understanding of interkingdom QS is limited.

*Candida* QS interactions with the respiratory pathogen *P. aeruginosa* have been extensively studied due to their frequent co-isolation in cystic fibrosis lungs, wound infections, indwelling devices and nosocomial infections^18–20^. Farnesol, a major QSM secreted by *C. albicans*, is known to supress *P. aeruginosa* by inhibiting its homoserine lactone synthesis that leads to subsequent reduction in bacterial swarming, and pyocyanin and quinolone signaling (PQS, 2-alkyl-4-quinolones)^15,20–22^. Farnesol also acts on *C. albicans* itself by inhibiting hyphal development (filamentation) through repression of adenylyl cyclase (Cyr1p) in the Ras1–cyclic AMP–protein kinase A pathway, which positively regulates hyphal growth^23^. In addition, farnesol triggers cellular oxidative stress and apoptosis in *C. albicans*. Exposure to azole antifungal agents significantly increases farnesol synthesis in *C. albicans*^24,25^, and recent studies have shown that nonlethal concentrations of farnesol enhance the efficacy of azole antifungals by suppressing ABC multidrug efflux transporters and accumulating reactive oxygen species (ROS)^26,27^. Interestingly, among the wide array of QSMs secreted by *P. aeruginosa*, N-(3-Oxododecanoyl)-L-homoserine lactone (C_16_H_27_NO_4,_ C12AHL) has a significant structural resemblance to farnesol^28,29^. Therefore, C12AHL also inhibits *C. albicans* hyphal development using the same mechanism as farnesol^28,29^. However, despite being structurally similar to farnesol, the effects of C12AHL on *C. albicans’* cellular mechanisms upon exposure to antifungal agents, including multidrug efflux activity, cellular fitness, and ergosterol synthesis (the molecular target of azoles), are largely unknown.

We recently demonstrated that the co-delivery of C12AHL with fluconazole in a liposomal drug carrier increases the efficacy of the antifungal agent in elimination of *C. albicans* biofilms. However, free forms of drug + C12AHL failed to demonstrate similar antifungal efficacy^30^ suggesting the effects of C12AHL on *C. albicans* upon exposure to antifungal agents are drug and C12AHL formulation dependent. Owing to the recognized clinical importance of *Pseudomonas*-*Candida* interactions in various pathological states, lack of synergistic effects of free C12AHL + fluconazole on *C. albicans* biofilms observed in our recent study^30^, and the sparsity of data on the role of *Pseudomonas* QSMs on *C. albicans* antifungal sensitivity/resistance, we evaluated the cellular and molecular responses of *C. albicans* on *in vitro* exposure to a widely-used anti-fungal fluconazole in the presence of the QSM C12AHL. We assessed the minimum inhibitory concentration (MIC) of the active agents (Fluconazole, C12AHL, C12AHL + fluconazole) using broth dilution assay with a checkerboard approach. *C. albicans’* multidrug efflux pump activity when exposed to the active agents was quantified by measuring the efflux of an indicator dye, rhodamine 6 g (Rhodamine 6 g Assay) and further verified based on the expression of genes coding for efflux pumps by qPCR. Changes in the *C. albicans* transcriptome in response to the active agents were assessed using next generation sequencing (RNA-Seq) and their effect on yeast protein synthesis was evaluated via two-dimensional gel electrophoresis and mass spectrometry. We demonstrate that *P. aeruginosa* C12AHL induces *C. albicans’* fluconazole resistance through multiple mechanisms, predominantly by facilitating fungal ergosterol synthesis and restoring its cell wall integrity.

## Results

### *C. albicans* sensitivity to fluconazole decreases in the presence of C12AHL

We hypothesised that C12AHL would make *C. albicans* more sensitive to fluconazole due to its known inhibitory properties on the yeast, therefore the minimum inhibitory concentrations (MIC50 and MIC80) for fluconazole in the presence and absence of C12AHL was determined. Unexpectedly, the MIC50 of fluconazole exhibited a 16-fold increase in the presence of 100 µg mL^−1^ C12AHL (0.156 µg mL^−1^ vs 2.5 µg mL^−1^, Supplementary Fig. S1 and Supplementary Table S2) and 8-fold increase in the presence of 12.5–50 µg mL^−1^ C12AHL (0.156 µg mL^−1^ vs 1.25 µg mL^−1^, Supplementary Fig. S1). No MIC80 of fluconazole was observed when *C. albicans* was exposed to the antifungal agent alongside C12AHL within the concentration ranges assessed in this study. Therefore, MIC80 of fluconazole appears to increase more than 8-fold when treated with C12AHL with a concentration range of 12.5–100 µg mL^−1^ (1.25 vs >10 µg mL^−1^, Supplementary Fig S1 and Supplementary Table S2). C12AHL demonstrated a 20% maximum inhibition of *C. albicans* growth when treated with 100 µg mL^−1^.

### C12AHL stimulates the multidrug efflux activity of *C. albicans*

**Figure 1:**
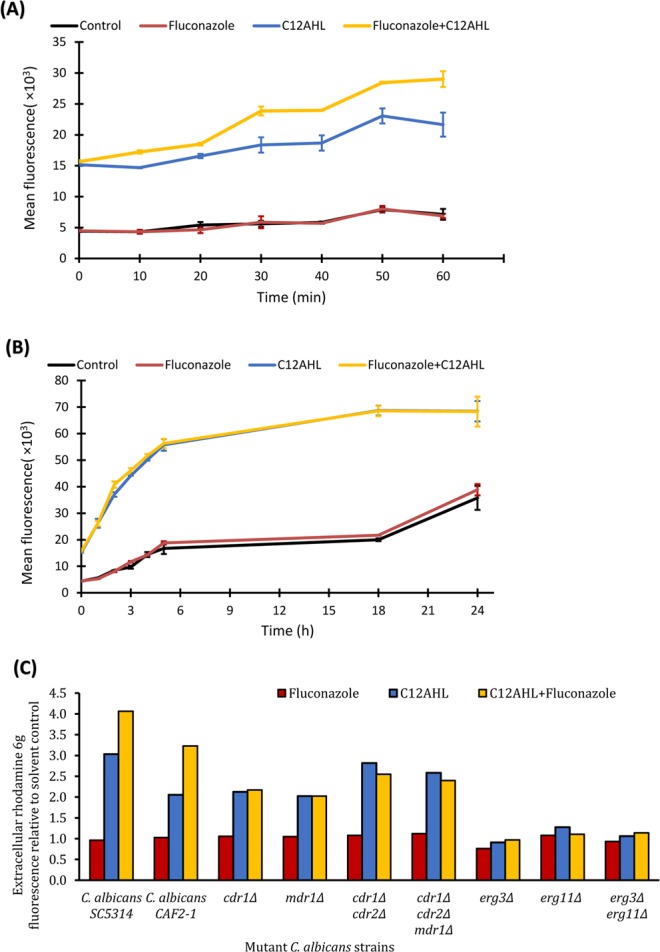
Mean drug efflux activity of *C. albicans* when exposed to C12AHL, fluconazole or their combination. (**A**) 1 h exposure, (**B**) 24 h exposure. Note the extracellular accumulation of significantly higher quantities of rhodamine 6 g when exposed to C12AHL or C12AHL + fluconazole compared to solvent control or fluconazole exposed samples. Standard deviations are presented as error bars (n = 18) (**C**) drug efflux activity of *C. albicans* mutants for a period of 1 h. There was a 2.0 to 2.8-fold increase in rhodamine 6 g efflux when *CDR* and *MDR* mutants were exposed to C12AHL or C12AHL + fluconazole for 1 h compared to solvent control, and insignificant changes in the *ERG* mutants (*p-value* < 0.05). *C. albicans* CAF2–1, the parental strain of the mutants tested, is included for comparison purposes.

Efflux of antifungal drugs via transport proteins is one of the main mechanisms employed by *C. albicans* when developing antifungal resistance^31^. Therefore, the activity of transport proteins was assessed in the presence of various treatment groups. When exposed to C12AHL or C12AHL + fluconazole, *C. albicans* pumped out significantly higher quantities of the indicator dyeR6G compared to fluconazole treated or the solvent (DMSO) controls for up to an exposure period of 24 h (Fig. 1A,B, P < 0.05). C12AHL + fluconazole exposure showed significantly higher R6G efflux compared to C12AHL treated *C. albicans* in the early stages of the exposure (up to 1 h of observation, Fig. 1A, P < 0.05). However, the latter difference appeared to wane during prolonged exposure to the QSM ± antifungal (up to 24 h of exposure, Fig. 1B, P > 0.05). In contrast, *C. albicans* exposed to fluconazole alone did not show any notable changes of rhodamine efflux compared to the control (Fig. 1A,B, P > 0.05).

Drug efflux pumps in *C. albicans* are mainly encoded by *CDR1, CDR2* and *MDR1*^32^, therefore, respective mutant strains were used to verify the efflux activity observed with the indicator dye. Efflux pump mutant strains of *C. albicans, cdr1Δ* (DSY448), *mdr1Δ* (DSY465)*, cdr1Δ/cdr2Δ* (DSY653), and *cdr1Δ/cdr2Δ/mdr1Δ* (DSY1050) demonstrated a 2.0 to 2.8-fold increase in rhodamine 6 g efflux when exposed to C12AHL or C12AHL + fluconazole for 1 h compared to the solvent control (Fig. 1C, P < 0.05). Exposure to fluconazole alone failed to increase R6G efflux significantly (Fig. 1C P > 0.05). Functions of the efflux pumps are known to be affected by the composition of cell membrane sterols (ergosterol in particular)^33,34^. Therefore, the mutant strains of *ERG11* and *ERG3*, the genes encoding rate limiting enzymes in ergosterol synthesis and sterol intermediates synthesis pathways^31^ were used to verify the role of sterols and the efflux activity observed with the indicator dye. Ergosterol mutant strains of *C. albicans*, *erg3Δ* (DSY1751)*, erg11Δ* (DSY1769), *erg3Δ erg11Δ* (DSY1764) did not exhibit significant changes in efflux activity when exposed to any of the treatments for 1 h compared to the solvent control (Fig. 1C, P > 0.05).

### C12AHL modulates the transcriptomic response of *C. albicans* when exposed to fluconazole

Transcriptomic sequencing was performed to determine which molecular mechanisms of *C. albicans* are modified in the presence of the QSM C12AHL, the antifungal fluconazole, or the combination of these molecules. First, an overall comparison of gene expression profiles was performed to assess whether there was an effect of the type of treatment. Then, differentially expressed genes were assessed for each treatment relative to the solvent control [(Fluconazole vs Control), (C12AHL vs Control), and (C12AHL + fluconazole vs Control)], as well as between treatments [(Fluconazole vs C12AHL), (Fluconazole vs C12AHL + fluconazole), and (C12AHL vs C12AHL + fluconazole)] to determine significant up- and/or downregulation of genes across the various treatments groups (adjusted *p-value* < 1e^−5^) (Supplementary Tables S3-S7).

**Figure 2:**
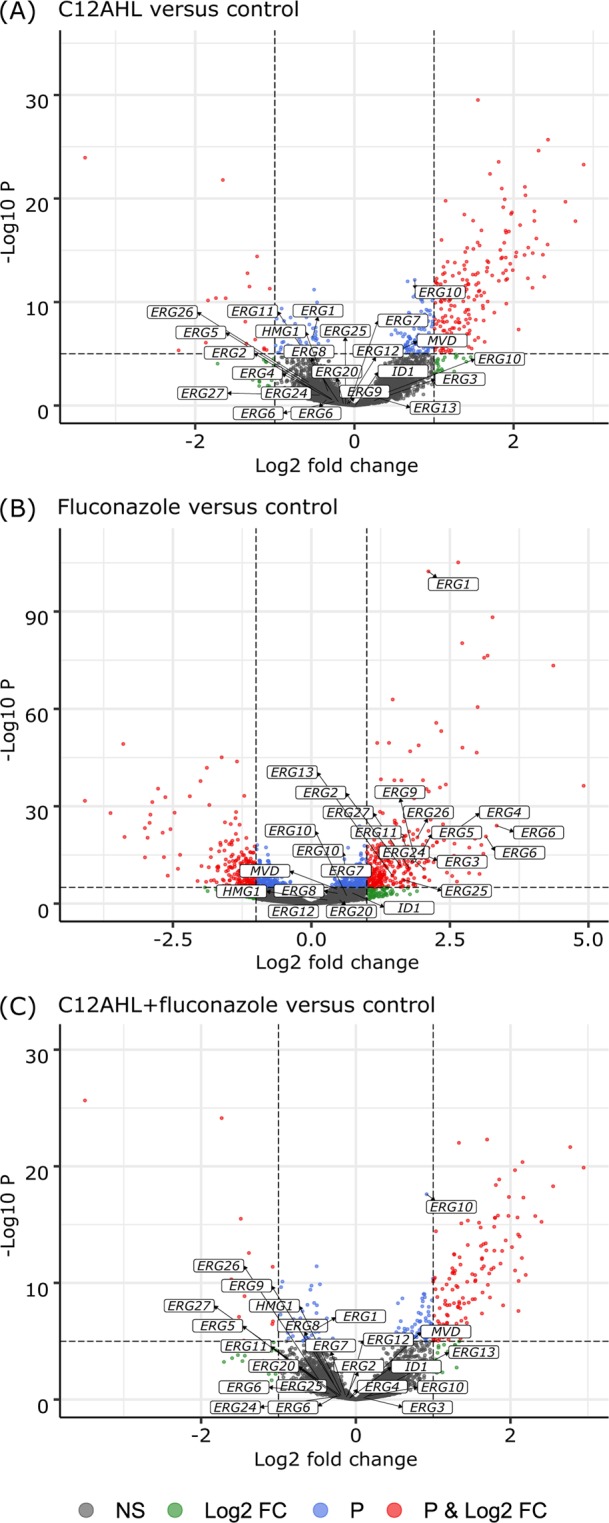
Comparison of gene expression profiles between each treatment and the control. Volcano plots showing RNA-Seq data from each treatment [(**A**) C12AHL, (**B**) fluconazole and (**C**) C12AHL + fluconazole] relative to the control. The dashed lines represent the cut-off values for *p-value* (=10^−6^) and log_2_ fold change (=2) to identify significantly different gene expression. The plots are coloured so that non-significant differentially expressed genes are represented in grey, those with log_2_ fold change >2 are shown in green, genes with *p-value* < 10^−6^ are coloured in blue, and those with both log_2_ fold change >2 and *p-value* < 10^−6^ are shown in red. Genes that represent proteins involved in the ergosterol biosynthesis pathway have been labelled in the plots.

**Figure 3:**
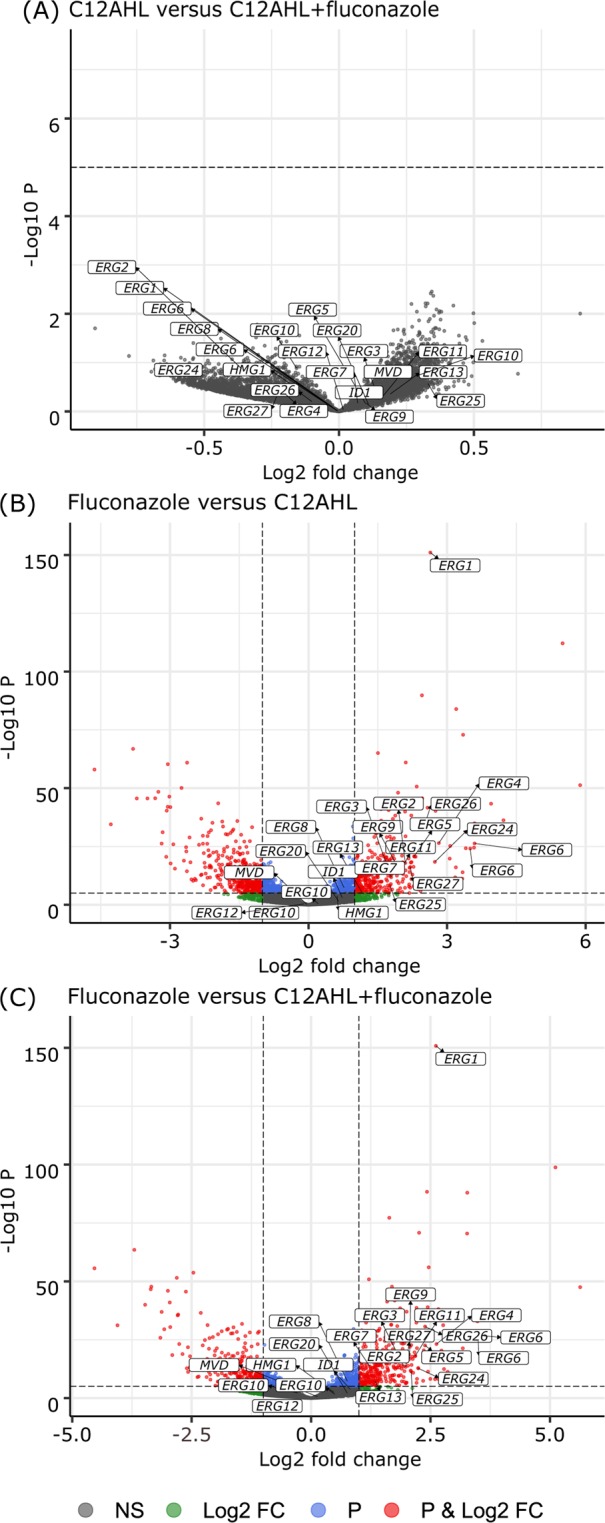
Comparison of gene expression profiles between the different treatments. Volcano plots showing the comparison of RNA-Seq data between the different treatments [(**A**) C12AHL vs C12AHL + fluconazole, (**B**) fluconazole vs C12AHL, and (**C**) fluconazole vs C12AHL + fluconazole]. The dashed lines represent the cut-off values for *p-value* (=10^−6^) and log_2_ fold change (=2) to identify significantly different gene expression. The plots are coloured so that non-significant differentially expressed genes are represented in grey, those with log_2_ fold change >2 are shown in green, genes with *p-value* < 10^−6^ are coloured in blue, and those with both log_2_ fold change >2 and *p-value* < 10^−6^ are shown in red. Genes that represent proteins involved in the ergosterol biosynthesis pathway have been labelled in the plots.

There was a significant influence of the type of treatment on the gene expression, with expression profiles for the fluconazole alone treated *C. albicans* being significantly different from the control, C12AHL and C12AHL + fluconazole samples (PERMANOVA, *p-value* < 0.05). This can also be observed graphically in a principle component analysis (Supplementary Fig. S8), where the fluconazole treated samples are statistically distinguishable from the control and other treatments in the second principle component (PC2), which accounts for almost a quarter of the total variation in the data. These results suggest that the effects of fluconazole were being ameliorated in the presence of C12AHL, and this is further confirmed when looking at the differentially expressed genes. Multiple genes were up- or downregulated in each treatment relative to the control samples, including the genes involved in ergosterol synthesis and antifungal resistance (Fig. 2, Supplementary Fig. S9 and S10, Supplementary Tables S3-S7). Genes were also differentially expressed between treatments with the exception of C12AHL vs C12AHL + fluconazole (Tables 1–3, Fig. 3, Supplementary Figs. S9 and S10). To determine the mechanisms of how C12AHL may be amending the effects of the antifungal molecule, we examined the differentially expressed pathways in more detail.

**Table 1:** Gene expression data; The *C. albicans* genes that were significantly affected by both fluconazole only and C12AHL only exposure compared to untreated controls [(Fluconazole vs Control) vs (C12AHL vs Control)]. Genes highlighted in blue are known to be associated with antifungal sensitivity/resistance. (http://www.candidagenome.org/).

Gene Name	Annotation	Fluconazole treated compared to control: log2FoldChange	Adjusted P value	C12AHL treated compared to control: log2FoldChange	Adjusted P value
*CSH1*	Aldo-keto reductase	3.36	5.11E-23	1.87	5.61E-07
*C3_03460C_A*	Protein of unknown function	3.35	5.16E-23	3.19	4.58E-20
*C1_01510W_A*	Protein of unknown function	2.78	1.81E-16	2.46	2.65E-12
*C1_04010C_A IFD1/IFD6 alias*	Protein with a NADP-dependent oxidoreductase domain	2.67	1.06E-19	1.68	1.47E-07
*LPG20*	Aldo-keto reductase family protein	2.44	4.3E-13	1.65	8.38E-06
*CRH11*	GPI-anchored cell wall transglycosylase	2.30	7.62E-08	−2.36	1.36E-07
*WOR4*	Predicted C2H2 zinc finger protein	2.09	7.17E-12	2.40	1.18E-14
*NRG1*	Transcription factor/repressor	1.86	1.93E-12	2.18	4.02E-16
*IFD6*	Aldo-keto reductase	1.75	6.62E-08	3.02	1.11E-21
*SRR1*	Two-component system response regulator	1.62	1.27E-06	2.24	1.98E-11
*C2_01750C_A*	Protein with a life-span regulatory factor domain	1.33	8.99E-07	1.51	4.57E-08
*RGS2*	Protein of RGS superfamily	1.29	1.1E-07	1.39	3.28E-08
*BCR1*	Transcription factor	1.26	3.25E-07	1.21	3.28E-06
*CR_10230W_A*	Histone acetyltransferase activity	0.85	3.82E-06	1.36	2.94E-14
*CDR4*	Putative ABC transporter superfamily	−1.43	2.18E-08	1.70	6.09E-11
*PHHB*	Putative 4a-hydroxytetrahydrobiopterin dehydratase	−1.62	5.69E-07	−1.72	2.87E-07
*IFR2*	Zinc-binding dehydrogenase	−1.79	2.02E-08	−1.78	8.07E-08
*RME1*	Zinc finger protein	−1.95	4.98E-07	2.17	3.28E-08
*ATX1*	Putative cytosolic copper metallochaperone	−2.22	8.01E-10	2.75	2.42E-14

**Table 2:** Gene expression data; The *C. albicans* genes that were significantly affected by both fluconazole only and C12AHL + fluconazole exposure compared to untreated controls [(Fluconazole vs Control) vs (C12AHL + fluconazole vs Control)]. Genes highlighted in blue are known to be associated with antifungal resistance/sensitivity. (http://www.candidagenome.org/).

Gene Name	Annotation	C12AHL-Fluconazole treated compared to control: log2FoldChange	Adjusted P value	Fluconazole treated compared to control: log2FoldChange	Adjusted P value
*C3_03460C_A*	Protein of unknown function	3.45	3.54E-23	3.35	5.16E-23
*IFD6*	Aldo-keto reductase	3.00	2.14E-21	1.75	6.62E-08
*ATX1*	Putative cytosolic copper metallochaperone	2.74	3.92E-14	−2.22	8.01E-10
*C1_01510W_A*	Protein of unknown function	2.41	1.41E-11	2.78	1.81E-16
*SRR1*	Two-component system response regulator	2.15	1.81E-10	1.62	1.27E-06
*WOR4*	Predicted C2H2 zinc finger protein	2.08	6.53E-11	2.09	7.17E-12
*RME1*	Zinc finger protein	1.98	9E-07	−1.95	4.98E-07
*NRG1*	Transcription factor/repressor	1.95	9.01E-13	1.86	1.93E-12
*CSH1*	Aldo-keto reductase	1.91	3.78E-07	3.36	5.11E-23
*CDR4*	Putative ABC transporter superfamily	1.76	1.68E-11	−1.43	2.18E-08
*LPG20*	Aldo-keto reductase family protein	1.71	3.87E-06	2.44	4.3E-13
*C1_04010C_A*	Protein with a NADP-dependent oxidoreductase domain	1.57	1.54E-06	2.67	1.06E-19
*RGS2*	Protein of RGS superfamily	1.17	7.97E-06	1.29	1.1E-07
*CR_10230W_A*	Histone acetyltransferase activity	1.07	8.15E-09	0.85	3.82E-06
*ENA21*	Predicted P-type ATPase sodium pump	−1.59	9.11E-06	−2.00	1.19E-09
*PHHB*	Putative 4a-hydroxytetrahydrobiopterin dehydratase	−1.78	1.04E-07	−1.62	5.69E-07
*IFR2*	Zinc-binding dehydrogenase	−1.82	4.01E-08	−1.79	2.02E-08

**Table 3:** Gene expression data; The *C. albicans* genes that were significantly affected by both C12AHL only and C12AHL + fluconazole exposure compared to untreated controls [(C12AHL vs Control) vs (C12AHL + fluconazole vs Control)]. Genes highlighted in blue are known to be associated with antifungal resistance/sensitivity. (http://www.candidagenome.org/).

Gene Name	Annotation	C12AHL-Fluconazole treated compared to control: log2FoldChange	Adjusted P value	C12AHL treated compared to control: log2FoldChange	Adjusted P value
*C3_03460C_A*	Protein of unknown function	3.45	3.54E-23	3.19	4.58E-20
*IFD6*	Aldo-keto reductase	3.00	2.14E-21	3.02	1.11E-21
*UGT51C1*	UDP-glucose:sterol glucosyltransferase	2.20	1.95E-19	2.38	1.45E-22
*CRZ2*	C2H2 transcription factor	2.30	2.41E-14	2.51	2.86E-17
*ATX1*	Putative cytosolic copper metallochaperone	2.74	3.92E-14	2.75	2.42E-14
*ALK2*	N-Alkane inducible cytochrome P450	1.93	2.75E-13	1.63	1.09E-09
*NRG1*	Transcription factor/repressor	1.95	9.01E-13	2.18	4.02E-16
*C1_01510W_A*	Protein of unknown function	2.41	1.41E-11	2.46	2.65E-12
*CDR4*	Putative ABC transporter superfamily	1.76	1.68E-11	1.70	6.09E-11
*YOR1*	ABC-type plasma membrane transporter	1.35	3.45E-11	1.41	2.33E-12
*WOR4*	Predicted C2H2 zinc finger protein	2.08	6.53E-11	2.40	1.18E-14
*ADA2*	Zinc finger and homeodomain transcriptional coactivator	1.51	8.98E-11	1.67	2.43E-13
*C6_02100W_A*	Secreted protein	−3.73	1.15E-10	−3.54	9.31E-10
*OPT3*	Oligopeptide transporter	2.16	1.39E-10	2.15	1.60E-10
*SRR1*	Two-component system response regulator	2.15	1.81E-10	2.24	1.98E-11
*C7_04090C_A*	Predicted mitochondrial cardiolipin-specific phospholipase	2.33	2.33E-10	2.35	1.59E-10
*C1_09210C_A*	Putative transporter	1.72	2.42E-10	1.47	8.96E-08
*C3_05450C_A*	Protein of unknown function	2.38	4.54E-10	2.53	2.17E-11
*ZCF1*	Zn(II)2Cys6 transcription factor	1.43	6.29E-10	1.59	2.33E-12
*RFG1*	HMG domain transcriptional repressor	1.81	7.32E-10	1.94	3.12E-11
*MOH1*	Ortholog of S. cerevisiae Moh1	1.61	2.14E-09	1.68	2.64E-10
*CR_09100C_A*	Aldo-keto reductase	2.30	4.70E-09	2.35	1.36E-09
*FBA1*	Fructose-bisphosphate aldolase	−1.79	5.31E-09	−1.64	8.92E-08
*EFG1*	bHLH transcription factor	1.88	7.64E-09	1.97	9.31E-10
*CR_10230W_A*	Ortholog(s) have histone acetyltransferase activity	1.07	8.15E-09	1.36	2.94E-14
*HSP78*	Heat-shock protein	2.23	1.33E-08	2.22	1.27E-08
*RPN4*	C2H2 transcription factor	1.34	1.56E-08	1.40	3.13E-09
*SNQ2*	Protein similar to S. cerevisiae Snq2p transporter	1.74	1.56E-08	1.55	6.73E-07
*CR_06140W_A*	Protein of unknown function	1.96	1.56E-08	1.98	1.08E-08
*C7_00770W_A*	Protein of unknown function	2.19	1.73E-08	1.81	6.81E-06
*HSP104*	Heat-shock protein	2.54	1.83E-08	2.47	3.99E-08
*GOR1*	Ortholog(s) have glyoxylate reductase activity	2.16	1.86E-08	2.00	2.37E-07
*IFR2*	Zinc-binding dehydrogenase	−1.82	4.01E-08	−1.78	8.07E-08
*AAF1*	Possible regulatory protein	2.13	5.63E-08	2.18	2.21E-08
*YIM1*	Protein similar to protease of mitochondrial inner membrane	1.95	6.00E-08	2.00	2.04E-08
*PHHB*	Putative 4a-hydroxytetrahydrobiopterin dehydratase	−1.78	1.04E-07	−1.72	2.87E-07
*ERO1*	Ortholog of S. cerevisiae Ero1	1.91	1.20E-07	2.00	1.82E-08
*MNN42*	Protein of unknown function	1.25	1.48E-07	1.43	6.88E-10
*HAL9*	Putative Zn(II)2Cys6 transcription factor	1.35	2.48E-07	1.40	5.22E-08
*ADH3*	Putative NAD-dependent (R,R)-butanediol dehydrogenase	3.05	2.81E-07	3.25	2.36E-08
*INO2*	Transcriptional activator	1.76	3.13E-07	1.92	1.24E-08
*CSH1*	Aldo-keto reductase	1.91	3.78E-07	1.87	5.61E-07
*C6_00290W_A*	Protein of unknown function	1.54	4.63E-07	1.67	2.04E-08
*GRP2*	Methylglyoxal reductase	2.50	4.86E-07	2.62	8.07E-08
*ISA1*	Putative mitochondrial iron-sulfur protein	1.39	7.93E-07	1.55	1.62E-08
*RME1*	Zinc finger protein	1.98	9.00E-07	2.17	3.28E-08
*C1_04010C_A*	Protein with a NADP-dependent oxidoreductase domain	1.57	1.54E-06	1.68	1.47E-07
*GAL102*	UDP-glucose 4,6-dehydratase	1.85	1.54E-06	2.05	3.84E-08
*C5_04030W_A*	Protein of unknown function	1.85	2.15E-06	2.08	3.99E-08
*C4_03020W_A*	Putative mitochondrial GTPase	0.98	2.64E-06	1.18	4.87E-09
*ALS7*	ALS family protein	0.95	2.83E-06	1.12	1.07E-08
*MNN12*	Predicted alpha-1,3-mannosyltransferase	−1.90	2.89E-06	−1.93	1.55E-06
*C3_02630C_A*	Protein of unknown function	1.20	3.87E-06	1.42	1.29E-08
*SIS1*	Putative Type II HSP40 co-chaperone	1.98	3.87E-06	1.94	5.25E-06
*LPG20*	Aldo-keto reductase family protein	1.71	3.87E-06	1.65	8.38E-06
*HSP90*	Essential chaperone	1.71	3.87E-06	1.85	2.86E-07
*MHP1*	Protein similar to S. cerevisiae Mhp1p	1.10	3.93E-06	1.09	4.69E-06
*C3_00360W_A*	Protein of unknown function	1.43	4.78E-06	1.88	2.21E-10
*C1_03990W_A*	Ortholog(s) have proteasome binding activity	1.64	6.48E-06	1.95	2.07E-08
*CR_06960W_A*	Ortholog(s) have ATP binding, DNA replication origin binding activity	1.29	6.48E-06	1.35	1.73E-06
*C1_01130W_A*	Putative ubiquitin ligase complex component	1.06	7.23E-06	1.09	2.80E-06
*RGS2*	Protein of RGS superfamily	1.17	7.97E-06	1.39	3.28E-08
*CR_07480W_A*	Predicted auxin family transmembrane transporter	1.02	9.33E-06	1.22	2.41E-08
*C4_02740W_A*	Protein of unknown function	1.48	9.33E-06	1.54	2.80E-06

### C12AHL modulates gene expression in the *C. albicans* ergosterol synthesis pathway upon exposure to fluconazole

**Figure 4:**
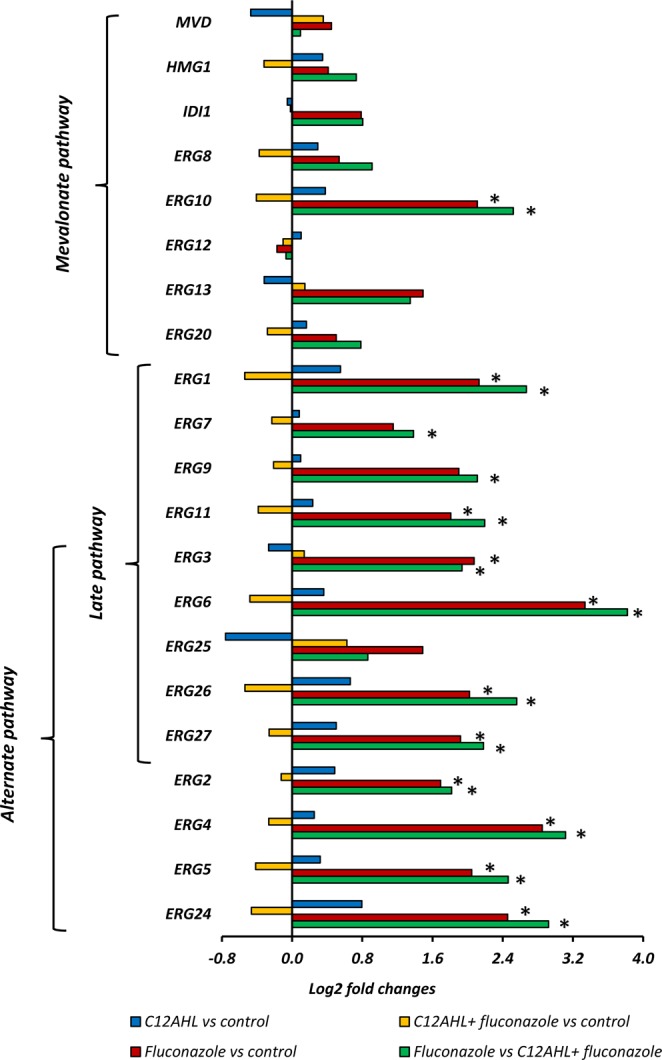
*C. albicans* molecular pathways analyses; The genes in the ergosterol biosynthesis pathway that are affected by fluconazole, C12AHL or C12AHL + fluconazole exposure. Comparisons denoted with * are significant (adjusted *p-value* < 1e^−5^). *ERG1*: Squalene epoxidase, *ERG2*: C-8 sterol isomerase, ERG3: C-5 sterol desaturase, *ERG4*: sterol C-24 reductase, *ERG5*: C-22 sterol desaturase, *ERG6*: Delta (24)-sterol C-methyltransferase, *ERG7*: 2,3-epoxysqualene-lanosterol cyclase (lanosterol synthase), *ERG8*: phosphomevalonate kinase, *ERG9*: farnesyl-diphosphate farnesyl transferase (squalene synthase), *ERG10*: Acetyl-CoA acetyltransferase, *ERG11*: Lanosterol 14-alpha-demethylase, *ERG12*: mevalonate kinase, *ERG13*: 3-hydroxy-3-methylglutaryl coenzyme A synthase, *ERG20*: farnesyl pyrophosphate synthetase, *ERG24*: C-14 sterol reductase, *ERG25*: C-4 methyl sterol oxidase, *ERG26*: C-3 sterol dehydrogenase, *ERG27*: 3-Keto sterol reductase, *HMG1*: HMG-CoA reductase, *IDI1*: isopentenyl-diphosphate delta-isomerase, and *MVD*: Mevalonate diphosphate decarboxylase.

**Figure 5:**
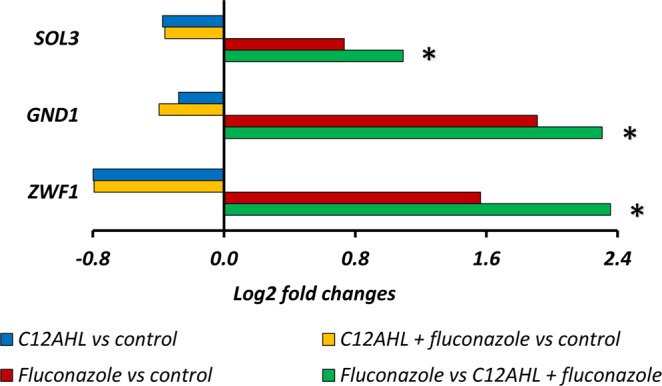
*C. albicans* molecular pathways analyses; Genes in the oxidative branch of the pentose phosphate pathway that are affected by fluconazole, C12AHL or C12AHL + fluconazole exposure. Comparisons denoted with * are significant (adjusted *p-value* < 1e^−5^). *ZWF1*: Glucose-6-phosphate dehydrogenase, *GND1*: 6-phosphogluconate dehydrogenase, and *SOL3*: 6-phosphogluconolactonase.

The *C. albicans* genes were collated into 156 different molecular pathways using published data from the *Candida* Genome Database. Once grouped, two molecular pathways in particular showed significant differences when comparing treatments, i.e. the ergosterol biosynthesis pathway and the pentose phosphate pathway. Comparison of genes involved in the ergosterol biosynthesis pathway (as shown in Fig. 4) revealed significant upregulation of *ERG1, ERG2, ERG4, ERG5, ERG6, ERG10, ERG11, ERG24, ERG26*, and *ERG27* in fluconazole treated samples compared to solvent controls, C12AHL treated, and C12AHL + fluconazole treated *C. albicans*. *ERG3* was upregulated in fluconazole treated *C. albicans* compared to solvent controls, while *ERG7* and *ERG9* were upregulated in fluconazole treated *C. albicans* compared to C12AHL + fluconazole treated samples (Fig. 4). Genes involved in the oxidative branch of the pentose phosphate pathway (*SOL3, GND1, ZWF1*), were significantly upregulated in fluconazole treated *C. albicans* compared to C12AHL treated and C12AHL + fluconazole treated *C. albicans* (Fig. 5).

### C12AHL alters protein expression in *C. albicans* exposed to fluconazole

In order to further assess the findings of the transcriptomic analyses, protein expression of *C. albicans* exposed to fluconazole with/without C12AHL was investigated. According to 2-dimensional electrophoresis and mass spectrometric data, addition of C12AHL led to differential expression of a variety of *C. albicans* proteins in the presence of fluconazole. A total of 17 under-expressed (Supplementary Table S11) and seven over-expressed (Supplementary Table S12) proteins were identified in the C12AHL + fluconazole treated *C. albicans* compared to the fluconazole only control (*p-value* < 0.05).

Several proteins that are known to be induced by fluconazole and/or other antifungals exposure (Gcy1p, Lsc1p, Pda1p, Atp1p, Mxr1p and Ach1p) were identified in fluconazole only treated *C. albicans* whereas they were absent in C12AHL + fluconazole treated samples (Supplementary Tables S11 and S12).

### C12AHL upregulates multidrug efflux pumps-coding genes in C. albicans exposed to fluconazole

Surprisingly, there was no indication of significantly increased expression of multidrug efflux pump genes at either the RNA or protein level after 24 h exposure to fluconazole in the presence of C12AHL. This is despite the observed increase in indicator dye efflux activity of the *C. albicans* strain in the presence of C12AHL + fluconazole. To understand whether there was any involvement of multidrug efflux pump genes on indicator dye efflux activity at the early stages of C12AHL + fluconazole exposure (1 h), the expression of these genes was assessed using qPCR^32^. Exposure to C12AHL + fluconazole for 1 h significantly upregulated *C. albicans CDR1* (2.4-fold) and *CDR2* (5-fold) genes compared to solvent controls (Supplementary Fig. S13, P < 0.05). C12AHL treated *C. albicans* also showed upregulation of *CDR2* (1.9-fold), however, *CDR1* and *MDR1* were downregulated compared to solvent controls. In contrast, both *CDR* genes were downregulated when exposed to fluconazole alone (*CDR1*:2.5 fold and *CDR2*:1.4-fold). *MDR1* expression was not affected by C12AHL + fluconazole treatment.

## Discussion

In host-associated environments, fungi and bacteria interact physically (e.g. co-aggregation) and chemically (e.g. quorum sensing), thereby impacting their immediate surroundings as well as the host immune response^35^. Such mutualistic/synergistic interactions have evolved to facilitate epithelial cohabitation and efficient use of metabolic by-products, while competitive antagonistic approaches have also developed during co-colonization^36^. Although the fundamental role of QS in optimizing microbial survival in polymicrobial environments has been well-studied, very little is known of the interactions and effects of interkingdom QS systems during antimicrobial therapy. Here we studied the effect of the *P. aeruginosa* quorum sensing molecule, C12AHL, on the cellular and molecular responses of *C. albicans* when exposed to the anti-fungal molecule fluconazole.

The role of C12AHL in interactions between *C. albicans* and *P. aeruginosa* has been increasingly studied in recent years. For example, it is known that *P. aeruginosa* cells preferentially adhere to *C. albicans* hyphae using their surface adherence proteins^37,38^. Using C12AHL defective mutants, Ovchinnikova *et al*. (2012) demonstrated that C12AHL is essential for the expression of *P. aeruginosa* adhesion proteins, and therefore is critical for adherence to *C. albicans* hyphae in a mixed fungal-bacterial environment^37^. Previous studies based on gas chromatography-mass spectrometric analyses, have reported that *P. aeruginosa* biofilms may contain over 600 µM (~178.5 µg mL^−1^) of peak C12AHL^28,39^. It is also noteworthy that QSM concentrations within the microbial populations vary depending on the different stages of biofilm growth, with lower concentrations during early stages of biofilm formation and biofilm dispersal, and higher concentrations during maturation. Subsequent studies have further suggested that >200 μM of C12AHL (59.5 µg mL^−1^) suppresses *C. albicans* hyphal growth whilst >500 μM (148.5 µg mL^−1^) of C12AHL inhibits fungal growth completely^28^. Hence, from the range of concentrations tested, we used sub-growth and sub-hyphal inhibitory concentration (50 µg mL^−1^, 168 µM) of C12AHL throughout our study. The inability to form hyphae with concentrations of C12AHL used (12.5–100 µg mL^−1^) was confirmed via a germ tube assay (Data not shown).

The 16-fold measured increase in the MIC of fluconazole indicates that *C. albicans* sensitivity to fluconazole decreases when it is simultaneously exposed to the antifungal and C12AHL (100 µg mL^−1^). Indeed, even at lower concentrations (12.5–50 µg mL^−1^) C12AHL induced fluconazole resistance in *C. albicans* compared to fluconazole-treated controls (Fig. 1A, *p-value* < 0.05). In our latest published work, we witnessed this lack of inhibitory properties of C12AHL and fluconazole when delivered to *C. albicans* biofilms as free forms without encapsulating into a drug carrier molecule (i.e. liposomes)^30^. Although the exact mechanism is yet to decipher, the synergy we observed with fluconazole and C12AHL in the form of liposomes is likely to be attributed to the characteristics of the liposomal formulation (i.e. enhanced penetration), slow and sustained drug release, the encapsulated dosage of the active agents and the intrinsic properties of *Candida* biofilm phenotype. This evidence further supported aforementioned hypothesis of fluconazole and C12AHL antagonism. In line with this finding, recent studies have also shown an increase in *C. albicans’* antifungal resistance in *C. albicans-P. aeruginosa* mixed species biofilms via upregulated *C. albicans* proteins associated with drug resistance and virulence^40^. However, it is known that *C. albicans* exhibits distinctive responses to antifungal agents and the environmental stresses (such as to bacterial QSMs) based on its physiological forms (i.e. biofilm vs planktonic phases). Therefore, in part, the lack of *C. albicans* inhibition observed with C12AHL ± fluconazole in our study compared to the existing *Candida* biofilm literature is likely to be associated the variations in the experimental design as this study focuses on planktonic phenotype, the usage of sub-growth and sub-hyphal inhibitory concentrations of C12AHL and other unknown fungal mechanisms. Accordingly, cautions must be taken when generalised inferences are made.

Intracellular R6G indicator dye accumulation is commonly used to identify azole-resistant *C. albicans* strains as it has been shown that the retention of R6G within fungal cells is inversely correlated with the expression of the drug efflux pump protein, Cdr1p, in *C. albicans*^41,42^. Our R6G assay reveals for the first time that exposure to the QSM C12AHL can upregulate the efflux activity of *C. albicans*. The presence of fluconazole in combination with C12AHL further enhances this increased efflux activity, indicating a potential mechanism of C12AHL-mediated fluconazole resistance in the yeast. Unlike C12AHL, its structural analogue farnesol produced by *C. albicans* is known to inhibit *C. albicans* drug efflux during fluconazole exposure, thereby potentiating the activity of the antifungal drug^26,27^. This result demonstrates the functional diversity of C12AHL and farnesol, despite their structural resemblance^43^.

*C. albicans* possesses three major plasma membrane drug efflux pump proteins: Cdr1p, Cdr2p [ATP-binding cassette (ABC) pumps] and Mdr1p [the major facilitator superfamily (MFS) transporters] that are known to regulate azole efflux^32^. Using a set of isogenic *C. albicans* strains lacking the genes for one or more of these drug efflux pumps (*CDR1, CDR2* and *MDR1*), Mukherjee *et al*. concluded that drug efflux pumps play a significant role in candidal resistance to fluconazole in early planktonic and biofilm phases (0–6 h)^44^. Our qPCR data indicated that short-term exposure (1 h) of *C. albicans* (azole-sensitive strain) to fluconazole in the presence of C12AHL upregulates the expression of *CDR1* and *CDR2*. This observation supports a previous study that showed upregulation of *CDR1* in *C. albicans* biofilms when exposed to *P. aeruginosa* secretory factors^45^. Taken together, these findings suggest that C12AHL ± fluconazole triggers phenotypic and transcriptional changes in multidrug efflux mechanisms in *C. albicans* within a short period of exposure (~1 h), suggesting a potential mechanism of early azole resistance. Further mechanistic investigations on *C. albicans* early antifungal resistance are necessary to confirm this hypothesis.

In contrast to previous reports on the temporal nature of the efflux activity and the absence of significant expression of *CDR1* and *CDR2* in the latter stages of planktonic/biofilm phases (e.g. 24 h), we noted that the efflux activity in *C. albicans* remained significantly higher for 24 h upon exposure to C12AHL ± fluconazole. Interestingly, transcriptomic data did not show significant changes in *CDR1, CDR2*, *MDR1*, *FLU1* or *TAC1* gene expression after 24 h treatment. In addition, we also noted that *C. albicans cdr1Δ*, *mdr1Δ, cdr1Δ/cdr2Δ*, and *cdr1Δ/cdr2Δ/mdr1Δ* strains continued to transport R6G outside of the cell, albeit to a lesser degree compared to the wild type, despite the respective mutations. Hence, the persistent efflux activity observed here is likely regulated via another mechanism(s).

Fungal ABC superfamily efflux pump proteins (Cdr1p in particular) are sensitive to imbalanced lipid composition in the yeast plasma membrane, and their mutual interactions with membrane sterols (mainly ergosterol) are critical for the sorting and functioning of efflux pump proteins in *C. albicans*^33,34^. Therefore, we hypothesized that increased efflux pump activity of *C. albicans* when exposed to C12AHL ± fluconazole is likely to be associated with changes in ergosterol biosynthesis. In order to test this, we assessed the R6G efflux activity of two mutant C. *albicans* strains with knockout of key genes involved in the ergosterol biosynthesis pathway (*ERG11* and *ERG3*). Interestingly, we noted that *erg11Δ, erg3Δ* and *erg3Δ/erg11Δ* strains were incapable of altering their efflux activity in response to 1 h exposure to C12AHL ± fluconazole, in contrast to the wild type. This suggests that the effect of C12AHL ± fluconazole on efflux pump activity may be associated with changes in the ergosterol synthesis pathway.

Azoles and many other antifungal drugs primarily target ergosterol biosynthesis in *C. albicans*. The sterol biosynthesis pathway possesses three distinct sub-pathways; mevalonate, late and alternate pathways (Supplementary Fig. S14). The mevalonate pathway, the first step in the sterol synthesis process, entails the production of farnesyl pyrophosphate (FPP) from acetyl-coenzyme A (acetyl-CoA)^46^. The resulting FPP is fed into many different cellular pathways as it is an essential intermediate in the biosynthesis of sterols (i.e. ergosterol), heme, ubiquinone, dolichol, and prenylated proteins^46,47^. The pathway responsible for the catalysation of FPP to synthesise ergosterol is identified as the late pathway. When antifungal agents such as azoles interfere with the late pathway, it branches out to the alternate pathway that produces sterol intermediates instead of ergosterol. Some of these sterol intermediates are known to be toxic and their intracellular accumulation arrests cell growth^48,49^.

Fluconazole suppresses C14α-demythylase encoded by *ERG11* in the late pathway, which normally catalyzes lanosterol to C14-demethyl-lanosterol and would ultimately lead to the synthesis of ergosterol. Suppression of *ERG11* reroutes the late pathway to the alternate pathway by expressing C24 methyl transferase (*ERG6*) with the synthesis of various sterol intermediates as a result. One particular intermediate is the toxic compound 14α-methyl-3,6-diol, which is catalysed by C5 desaturase *(ERG3*) in the final step and ultimately arrests fungal growth^46^. Ergosterol is a major sterol component of the yeast cell wall and mitochondrial membrane, and is vital in maintaining membrane fluidity and permeability, enzyme activity, cell cycle progression and cell morphology^50^. In addition, sterols and sphingolipids together form lipid rafts, i.e. a type of microdomain located in the fungal cell membrane, that is enriched with numerous molecules such as efflux pumps, sodium and potassium pumps, receptors, and nutrient transporters^51,52^.

In this study, gene and protein expression data provided strong evidence to suggest that C12AHL mediated induction of fluconazole resistance in *C. albicans* is associated with ergosterol biosynthesis. Previous studies have established that prolonged exposure to azoles (fluconazole, itraconazole, ketoconazole, clotrimazole, and miconazole) can trigger over expression of *ERG11* and other genes associated with the alternate pathway of sterol synthesis *(ERG9*, *ERG1*, *ERG7, ERG3)*, particularly during the logarithmic growth phase of the yeast^50,53,54^. Our gene expression data also demonstrated similar findings, for example, all genes of both the late and alternate pathways (except *ERG25*) of sterol biosynthesis were significantly upregulated when *C. albicans* was exposed to fluconazole but remained unaffected with either C12AHL + fluconazole or C12AHL exposure. Therefore, these results suggest that the effect of fluconazole on *C. albicans’* late and alternate pathways of sterol synthesis is suppressed in the presence of C12AHL. Functional investigations using relevant key mutant strains of the ergosterol synthesis pathway could provide valuable mechanistic insights to further support the observed changes in gene expression.

The enzymes that catalyse the sterol biosynthesis pathway are regulated in part by the zinc-cysteine finger transcription factor paralogs Upc2p in *C. albicans*^55,56^. Upc2p senses sterol levels within the yeast and when these levels are reduced, for example due to fluconazole interference, it activates genes for sterol biosynthesis and uptake^55,56^. Our gene expression data confirmed significant upregulation of *UPC2* (codes for Upc2p) in fluconazole treated *C. albicans* as a result of fluconazole-mediated inhibition of ergosterol synthesis. This finding explains why not only genes in the alternate pathway were upregulated in the fluconazole treated samples, but there was also indirect upregulation of the genes in the late pathway. Experiments using *UPC2* mutant *C. albicans* could be performed to further confirm this observation.

Notably, neither C12AHL nor C12AHL + fluconazole treated samples elicited the changes observed in the presence of fluconazole alone, indicating that the regulation of sterol biosynthesis/uptake in the yeast in the presence of the QSM was unaffected. This is further evidenced by no significant change in the expression of *ECM33* relative to the control, which codes for protein molecules within lipid rafts that are sensitive to changes in the cell membrane composition. The maintenance of lipid rafts is critically important for proper functioning of a variety of cellular processes, cell signalling, protein sorting, virulence, stress responses, and environmental adaptations^33,34,51,57^. *ECM33* is known to be significantly upregulated during exposure to fluconazole as observed in our data^58^. We also noted an upregulation of *UGT51C1* in C12AHL + fluconazole treated but not in fluconazole treated *C. albicans. UGT51C1* codes for UDP-glucose:sterol glucosyltransferase that catalyses the biosynthesis of sterol glycosides from ergosterol^59^. Upregulation of this gene indicates that there is likely to be a continual supply of ergosterol as substrate to the enzyme, thus further supporting the hypothesis of unaffected ergosterol synthesis in *C. albicans* by fluconazole when C12AHL is present.

*C. albicans* can use three antioxidant systems (i.e. catalase, thioredoxin and glutathione) and two major oxidative stress signalling pathways (i.e. Cap1 and Hog 1) to respond to oxidative stress induced by antifungals. Oxidative stress induced by antifungals stimulates NADPH production in *C. albicans* via the oxidative branch of the pentose phosphate pathway (PPP)^60–62^. NADPH is an essential cofactor for glutathione- and thioredoxin-dependent enzymes in antioxidant systems (thioredoxin and glutathione, respectively) that neutralize reactive oxygen species (ROS)^63–65^. Therefore, the oxidative branch of the PPP is critical for fungal survival against oxidative stress^66,67^. Glucose-6-phosphate-1-dehydrogenase coded by *ZWF1* regulates the rate limiting first step of the oxidative branch of PPP^68^ and the gene expression profiles from this experiment showed significant upregulation of genes in the oxidative arm of the PPP, in particular *ZWF1*, in the fluconazole treated samples. This effect was however not observed when C12AHL was present. In addition, as observed in the protein expression data, several key proteins that play a role in protecting the fungus from oxidative stress, Sod1p (superoxide dismutase^69^), Pst1p (Flavodoxin-like protein^70^), Mxr1p (methionine sulfoxide reductase^62^, Adh4p (alcohol dehydrogenase^71^), and Cyp5p (Peptidyl-prolyl cis-trans isomerase^72^), were downregulated in *C. albicans* treated with C12AHL + fluconazole compared to fluconazole alone treated samples. These results suggest that the presence of C12AHL prevents the oxidative stress otherwise imposed by fluconazole on the yeast cells.

Another interesting finding was that the presence of C12AHL appears to increase the overall fitness of *C. albicans* when challenged with fluconazole. For instance, we observed significant upregulation of genes *GAL102, C2_00770W_A*, and *DAG7* that lower the sensitivity of the yeast to toxic sterol analogues accumulated via the alternate pathway^73,74^. Similarly, *GAL102* plays an important role in yeast cell wall synthesis and resistance to antifungal drugs by stabilizing the cell wall^73^. Upregulation of *GAL102* together with other genes that are known to regulate yeast cell wall synthesis and repair (i.e. *INO2, ADA2, PHR1* and *MNN12*) may further indicate that the presence of C12AHL prevents the impact of fluconazole on yeast cell wall integrity and improves the overall cellular fitness^75,76^.

In summary, our data suggest that the presence of C12AHL favorably affects *C. albicans* challenged with fluconazole by preventing changes in sterol biosynthesis, increasing drug efflux pump activity, reducing the oxidative stress response, and maintaining yeast cell membrane integrity. These conclusions are largely based on our transcriptomic data; therefore, appropriate functional assessments as indicated are necessary to verify these claims. Further investigations on sterol analyses (including total cellular sterol and sterol intermediates) as well as changes in plasma membrane composition of the yeast are necessary to confirm this hypothesis. In addition, recent studies have highlighted some of the complex interactions between *C. albicans* and *P. aeruginosa* in polymicrobial infections. For example, certain compounds produced by *C. albicans*, that remain to be characterized, have been shown to stimulate the synthesis of virulence factors (e.g. phenazine production) by *Pseudomonas* spp., as well as to reduce swarming motility which leads to enhanced biofilm development^20,77^. Therefore, further investigations on *C. albicans* and *P. aeruginosa* cocultured in a polymicrobial biofilm environment would be beneficial to understand the specific interactions between these two microorganisms when exposed to fluconazole. Selective physical interactions between *P. aeruginosa* and *C. albicans* filaments, together with mutual inhibitory and beneficial effects of the QSMs C12AHL and farnesol, speak to the importance of co-existence and the interdependence of *P. aeruginosa* and *C. albicans* for their survival in mixed microbial communities. Hence, the core finding of our study, that C12AHL induces antifungal resistance in *C. albicans*, thereby protecting the fungal population, is likely to be another control mechanism employed by *P. aeruginosa* in optimizing its survival in challenging polymicrobial environments.

## Material and methods

### Microorganisms and quorum sensing molecules

*C. albicans* SC5314 (a fluconazole sensitive strain) was used throughout this study. Microbial identity was reconfirmed with commercially available API 32 C for *Candida* strains (Biomérieux, Mercy I’Etoile, France). Mutant *C. albicans* strains DSY448, DSY465, DSY654, DSY1050, DSY1751, DSY1764, DSY1769 and parental strain *C. albicans* CAF2–1 (Supplementary Table S15) were kindly gifted by Associate Professor Dominique Sanglard from the Institute of Microbiology, University Hospital Lausanne, Switzerland. All isolates were stored in multiple aliquots at –70 °C, after confirming their purity.

C12AHL from *P. aeruginosa* (Catalogue No. O9139) and fluconazole (Catalogue No. F8929) were purchased from Sigma Aldrich (St. Louis, MO), dissolved in dimethyl sulfoxide (DMSO) and stored at −20 °C until further use.

### Growth media

Sabouraud dextrose agar and yeast nitrogen base with amino acids (YNB; Catalogue No. Y1250; Sigma Aldrich, St. Louis, MO) solution supplemented with 100 mm glucose were used for culturing *C. albicans*. RPMI 1640 media supplemented with MOPS (morpholinepropanesulfonic acid) was used for broth microdilution assays.

### Yeast inocula

Before each experiment, both *C. albicans* wild type and mutant strains were subcultured on Sabouraud Dextrose Agar for 18 h at 37 °C. A single colony from overnight *C. albicans* growth was inoculated into YNB medium and incubated for 18 h in an orbital shaker (150 rpm) at 37 °C. The resultant culture was harvested, washed twice in phosphate-buffered saline (PBS, pH 7.2) and resuspended in YNB. Cell suspensions were adjusted to 1 × 10^7^ cells mL^−1^ (standard unless otherwise specified) by spectrophotometry and confirmed by hemocytometric counting.

### Determination of minimum inhibitory concentration (MIC)

The MIC was determined by a broth microdilution assay in accordance with the CLSI guidelines^78^. Briefly, *C. albicans* suspensions (1 × 10^3^ cells mL^−1^) were treated with fluconazole, C12AHL or both using a checker-board approach (C12AHL: 12.5 µg mL^−1^–100 µg mL^−1^ and Fluconazole: 0.078 µg mL^−1^–80 µg mL^−1^) and incubated in a 96-well microtiter plate for 24 h at 37 °C. At the end of this incubation, the optical density of the fungal growth was measured spectrometrically at 595 nm and MICs were determined. The MIC50 and MIC80 were defined as the lowest concentration of the tested agent that inhibited 50% and 80%, respectively, of fungal growth compared to solvent controls. The assay was performed as quadruplicates three separate times (n = 12).

### Treatment groups and doses

Three test groups (fluconazole, C12AHL, fluconazole+C12AHL) and one solvent control group (DMSO; the solvent for C12AHL and fluconazole) were used. Following concentrations were used throughout the study unless otherwise specified; 1.25 µg mL^−1^ fluconazole, 50 µg mL^−1^ C12AHL, 1.25 µg mL^−1^ fluconazole + 50 µg mL^−1^ C12AHL, or DMSO (Control, 2% V/V). The chosen concentration of fluconazole is the minimum concentration required to inhibit 80% of *C. albicans* cells (MIC80). A sub-growth and sub-hyphal inhibitory concentration of C12AHL (50 µg mL^−1^, 168 µM)^28^ was chosen to prevent growth or hyphae development associated effects on *C. albicans*.

### Drug efflux activity assay

The activity of *C. albicans* drug efflux pumps when treated with C12AHL and/or fluconazole was assessed using an indicator dye, rhodamine 6 g (R6G), as described by Holmes, A. R. *et al*. 2018^79^. Briefly, standard suspensions of *C. albicans* SC5314 and mutant strains DSY448, DSY465, DSY654, DSY1050, DSY1751, DSY1764 and DSY1769 were prepared in PBS and starved for 2 h at room temperature (25 °C). R6G was added (10 µM final concentration) and incubated in dark conditions for further 1 h at 37 °C and 200 rpm. At the end of the incubation, cells were washed three times with PBS, resuspended and 100 µl was added to wells in a 96 well plate. Rhodamine 6 g loaded *C. albicans* were treated with either fluconazole, C12AHL, C12AHL + fluconazole, or DMSO and the plate was incubated at 37 °C in the dark. After 5 min of post-treatment, 1 mM glucose was added to each well and further incubated. The cell suspensions were removed at given time points (every 10 min up to 1 h, hourly up to 5 h, 18 h, and 24 h), centrifuged (10 min, 13000 rpm, 25 °C), and the amount of R6G released into the supernatant was read using a spectrophotometer at 485 nm excitation/535 nm emission. Each assay was conducted in sextuplicate at 3 different occasions (n = 18).

## Gene expression analyses

### Next generation sequencing (RNA-Seq)

Changes in the *C. albicans* transcriptome were assessed with next generation sequencing (RNA-Seq). *C. albicans* SC5314 standard suspension was prepared as described above, treated with either fluconazole, C12AHL, C12AHL + fluconazole, or DMSO and incubated at 37 °C statically for 24 h. Cells were washed 3 times in PBS and total RNA was extracted using the SV total RNA isolation system (Catalog No. Z3100, Promega, Madison, WI). Three biological replicates were processed for each treatment group. RNA-Seq libraries were prepared using Illumina ScriptSeq Complete Gold (Yeast) Kit (Illumina, Inc., San Diego, CA) according to manufacturer’s instructions. One µg of total RNA from each sample was used for library preparation. All libraries were sequenced 2 ×150 bp high output v2 kit (100 Gb) on the Illumina NextSeq 500 platform.

Reads were mapped to the *Candida* genome using the RNA-seq processing pipeline STAR v2.5.2a^80^ in AlignReads mode with a maximum intron size of 30Kb. Gene expression was quantified by counting reads using htseq v0.6.1^80^ for all genes in the *Candida* Genome Database (http://www.candidagenome.org, Stanford Genome Technology Centre) gene model *C. albicans SC5314* version A22-s07-m01-r30. RNA-Seq data was analyzed in R v3.6.1 (https://www.R-project.org/)^81^ by broadly exploring differences between samples using principle component analysis after normalizing read counts into centered log ratio values. Statistical comparison of gene expression between treatment types was performed using PERMANOVA. Differentially expressed genes were then identified using DESeq. 2 v1.14.1^82^, with the sample group set as the design formula and contrasts between groups used to identify differentially abundant genes, and visualized using EnhancedVolcano v1.2.0 (https://github.com/kevinblighe/EnhancedVolcano)^83^. The *Candida* genome database was used to determine pathways affected by the different treatments, using an adjusted *p-value* < 1e^−5^ as the cut-off for statistically significant gene expression comparison.

### Real‐time PCR assay

Changes in the expression of *CDR1, CDR2* and *MDR1* (the genes coding *C. albicans* drug efflux pumps) when exposed to fluconazole, C12AHL or C12AHL + fluconazole for a shorter duration (1 h) were quantitatively assessed by real‐time polymerase chain reaction (qPCR). *C. albicans* SC5314 suspensions (1 × 10^3^ cells mL^−1^) were prepared as mentioned above and treated with fluconazole, C12AHL or both (C12AHL + fluconazole) for 1 h at 37 °C in static conditions. Cells were harvested, washed 3 times with PBS, and RNA was extracted using the SV total RNA isolation system (Catalog No. Z3100, Promega, Madison, WI) using 2 μg template for reverse transcription with Superscript II (Invitrogen, Carlsbad, CA). qPCR was performed as described previously^84^ using primers shown in Supplementary Table S16. Relative gene expression was quantified using *EFB1* as the housekeeping (reference) gene^85^. All experiments were carried out in duplicate on three different occasions (n = 6).

### Protein expression analysis

The changes in *C. albicans* protein expression when treated with C12AHL + fluconazole compared to fluconazole were assessed with 2-dimensional gel electrophoresis and mass spectrometry. *C. albicans* SC5314 standard suspension was prepared as described above, treated with either fluconazole or C12AHL + fluconazole and incubated at 37 °C statically for 24 h. At the end of the incubation, cells were washed 3 times with PBS and total protein were extracted, after which first‐ and second-dimension electrophoreses were performed as described previously^84^. Protein gels derived from three biological replicates (with three technical replicates) from each group were analysed using a two‐dimensional analysis software (PD Quest; Bio‐Rad Laboratories). Protein spots were identified using default settings and verified manually to eliminate background noise and artefacts. Only consistent and reproducible protein spots (in all replicates) were progressed for further analysis, and spots that were missing in either fluconazole or fluconazole + C12AHL samples (in all replicates) were considered as differentially expressed in *C. albicans* in response to respective exposure^84^.

Proteins that were differentially expressed were in-gel digested, peptides were extracted and subjected to tandem mass spectrometry as described previously^84^. Briefly, matrix‐assisted laser desorption/ionization time‐of‐flight mass spectroscopy/mass spectroscopy (MALDI TOF MS/MS) was performed using a Bruker Autoflex III MALDI TOF/TOF Mass Spectrometer (Bruker Daltonics, Bremen, Germany) and Dionex UltiMate 3000 nano‐LC system using a 50‐Hz frequency laser beam. Candidate proteins were identified in the NCBI nr database using Mascot software (http://www.matrixscience.com/) (parameters used: Type of search: MS/MS Ion Search, Enzyme: Trypsin/P, Fixed modifications: Carbamidomethyl (C), Variable modifications: Oxidation (M), Mass values: Monoisotopic, Protein Mass: Unrestricted, Peptide Mass Tolerance: ±50 ppm, Fragment Mass Tolerance: ±0.5 Da, Max Missed Cleavages: 1, Instrument type: MALDI‐TOF‐TOF). Protein scores were derived from ion scores as a non‐probabilistic basis for ranking the protein hits at a significance level of *p-value* < 0.05^84^. Identified proteins were functionally characterized, and encoding genes were determined using the *Candida* genome database, NCBI database (http://www.ncbi.nlm.nih.gov/), SWISSPROT and TrEMBL non‐redundant protein databases (http://www.expasy.ch/sport/)^84^.

### Statistical analyses

All other assays not mentioned in the sections above were performed using nonparametric Mann—Whitney U-tests with SPSS software (version 16.0) for comparison of test conditions to corresponding control groups. A *p-value* < 0.05 was considered statistically significant.

## Supplementary information

Supplementary Information.

## Data Availability

Sequencing data that support the findings of this study have been deposited in NCBI Sequence Read Archive (SRNA) under Bio Project Accession No. PRJNA599446. (https://www.ncbi.nlm.nih.gov/bioproject/599446).
