# Supplementary material for: Fluconazole resistance in Candida albicans is induced by Pseudomonas aeruginosa quorum sensing
Source: Sci Rep. 2020 May 8;10:7769. doi: 10.1038/s41598-020-64761-3 (PMC7211000; doi:10.1038/s41598-020-64761-3)
Supplement: Supplementary file 1 — Supplementary Information. [file 41598_2020_64761_MOESM1_ESM.pdf]

Supplementary materials

Fluconazole resistance in *Candida albicans* is induced by *Pseudomonas aeruginosa* quorum sensing

H. M. H. N. Bandara[1]*, D. L. A. Wood[2], I. Vanwonterghem[2], P. Hugenholtz[2], B.P. K. Cheung[3], and L. P. Samaranayake[4]

[1.] Oral Microbiology, Bristol Dental School, University of Bristol, Lower Maudlin Street, Bristol BS1 2LY, UK

[2.] Australian Centre for Ecogenomics, School of Chemistry and Molecular Biosciences, The University of Queensland, St Lucia QLD 4072, Australia

[3.] Faculty of Dentistry, The University of Hong Kong, 34 Hospital Rd, Sai Ying Pun, Hong Kong SAR, China

[4.] College of Dental Medicine, The University of Sharjah, P.O. Box: 27272, Sharjah, UAE

*Corresponding author;

Dr H. M. H. N. Bandara

Address: Bristol Dental School, Lower Maudlin Street, Bristol BS1 2LY, United Kingdom

Telephone: +44 117 342 9497

E mail: nihal.bandara@bristol.ac.uk

Supplementary materials

Supplementary Figure S1: Minimum inhibitory concentrations (MIC50 and MIC80) of fluconazole against *C. albicans* in the presence of various concentrations of C12AHL (0 – 100 µg mL^{-1}).

Supplementary Table S2: Minimum inhibitory concentrations (MIC50 and MIC80) of fluconazole, C12AHL or Fluconazole+C12AHL against *C. albicans*.

Supplementary Table S3: Gene expression data; Significantly upregulated/downregulated genes in *C. albicans* treated with fluconazole compared to untreated controls. The genes that are known to be associated with antimicrobial sensitivity/resistance are shaded in light blue (adjusted p-value < $1e^{-5}$).

Supplementary Table S4: Gene expression data; Significantly upregulated/downregulated genes in *C. albicans* treated with C12AHL compared to untreated controls. The genes that are known to be associated with antimicrobial sensitivity/resistance are shaded in light blue (adjusted p-value < $1e^{-5}$).

Supplementary Table S5: Gene expression data; Significantly upregulated/downregulated genes in *C. albicans* treated with C12AHL+fluconazole compared to untreated controls. The genes that are known to be associated with antimicrobial sensitivity/resistance are shaded in light blue (adjusted p-value < $1e^{-5}$).

Supplementary Table S6: Gene expression data; Significantly upregulated/downregulated genes in *C. albicans* treated with C12AHL compared to fluconazole only treated samples. The genes that are known to be associated with antimicrobial sensitivity/resistance are shaded in light blue (adjusted *p-value* < $1e^{-5}$).

Supplementary Table S7: Gene expression data; Significantly upregulated/downregulated genes in *C. albicans* treated with C12AHL+fluconazole compared to fluconazole treated samples. The genes that are known to be associated with antimicrobial sensitivity/resistance are shaded in light blue (adjusted p-value < $1e^{-5}$).

Supplementary Figure S8: Principle component analysis of *C. albicans* gene expression. The gene expression profiles for the control, C12AHL, C12AHL+fluconazole, and fluconazole only treatments were statistically compared using a principle component analysis to identify variability in gene expression between treatments. Each colour represents a different treatment as indicated in the right-side panel, and each circle represents the gene expression for one of the replicates per treatment.

Supplementary Figure S9: Comparisons among significantly downregulated genes in *C. albicans* exposed to fluconazole, C12AHL or C12AHL+fluconazole. Genes that are highlighted in red are known

to be associated with antifungal resistance (adjusted p-value < $1e^{-5}$). Genes that were significantly downregulated between treatments are shown in Supplementary Tables 2-6.

Supplementary Figure S10: Comparisons among significantly upregulated genes in *C. albicans* exposed to fluconazole, C12AHL or C12AHL+fluconazole. Genes that are highlighted in red are known to be associated with antifungal resistance (adjusted p-value < $1e^{-5}$). Genes that were significantly upregulated between treatments are shown in Supplementary Tables 2-6.

Supplementary Table S11: Protein expression data; Under-expressed proteins in C12AHL+fluconazole treated *C. albicans* samples compared to fluconazole only treated samples.

Supplementary Table S12: Protein expression data: Over-expressed proteins when treated with C12AHL+fluconazole + *C. albicans* compared to fluconazole only treated samples.

Supplementary Figure S13: Differential expression of *CDR1, CDR2* and *MDR1* to C12AHL, fluconazole or their combination. Real-time quantitative PCR assay data. Note the significant upregulation of *C. albicans CDR1* and *CDR2* when exposed to C12AHL+fluconazole and *CDR2* when exposed to C12AHL compared to solvent controls ($P<0.05$).

Supplementary Figure S14: *C. albicans* ergosterol synthesis pathway. Significantly upregulated genes when exposed to fluconazole compared to C12AHL+fluconazole treated *C. albicans* appear in bold.

Supplementary Table S15: *Candida albicans* strains used in this study

Supplementary Table S16: The primers used for the Real-time quantitative PCR in this study

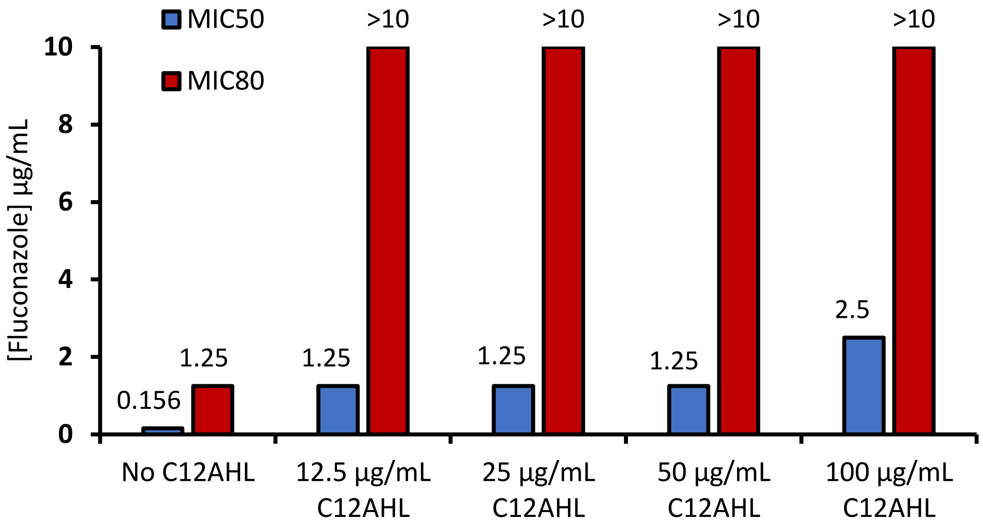

Supplementary Figure S1: Minimum inhibitory concentrations (MIC50 and MIC80) of fluconazole against *C. albicans* in the presence of various concentrations of C12AHL (0 – 100 µg mL^{-1}).

**Percentage inhibition of *C. albicans***	**Fluconazole only µg/ml (µM)**	**Fluconazole with 100µg/ml (336µM) C12AHL**	**Fluconazole with 50µg/ml (168µM) C12AHL**	**Fluconazole with 25µg/ml (84µM) C12AHL**	**Fluconazole with 12.5µg/ml (42µM) C12AHL**
**50**	0.156 (0.5µM)	2.5 (8.2µM)	1.25 (4.1µM)	1.25 (4.1µM)	1.25 (4.1µM)
**80**	1.25 (4.1µM)	>10 (>32.6µM)	>10 (>32.6µM)	>10 (>32.6µM)	>10 (>32.6µM)

Supplementary Table S2: Minimum inhibitory concentrations (MIC50 and MIC80) of fluconazole, C12AHL or Fluconazole+C12AHL against *C. albicans*.

Name	Annotation	log2 Fold Change	Adjusted P value
C3_01540W_A	Plasma-membrane-localized protein	5.43	8.21E-29
PGA13	GPI-anchored cell wall protein	4.43	3.04E-25
C2_10160W_A	Secreted protein	4.17	4.98E-25
MRV8	Protein of unknown function	4.16	1.95E-13
PGA23	Putative GPI-anchored protein of unknown function	3.99	4.69E-18
PGA45	Putative GPI-anchored cell wall protein	3.92	9.61E-12
C3_06660C_A	Protein of unknown function	3.77	1.96E-08
C7_03310W_A	Protein of unknown function	3.53	6.54E-10
C2_02910W_A	Protein of unknown function	3.44	4.87E-15
MNN4-4	Mannosyltransferase	3.37	2.70E-18
CSH1	Aldo-keto reductase	3.36	5.11E-23
C3_03460C_A	Protein of unknown function	3.35	5.16E-23
ERG6	Delta(24)-sterol C-methyltransferase	3.34	8.85E-21
C6_03260W_A	Putative ribonuclease H1	3.26	2.77E-23
DDR48	Immunogenic stress-associated protein	3.22	1.60E-10
IPK2	Putative inositol polyphosphate multikinase	3.21	3.69E-34
RBT4	Pry family protein	3.15	1.13E-11
RBR1	Glycosylphosphatidylinositol (GPI)-anchored cell wall protein	2.97	1.62E-08
C1_11260C_A	Putative adhesin-like protein	2.92	3.67E-17
DAG7	Secretory protein	2.88	6.51E-08
ERG4	Protein similar to sterol C-24 reductase	2.85	1.00E-09
UPC2	Zn2-Cys6 transcript factor	2.79	1.21E-48
C1_01510W_A	Protein of unknown function	2.78	1.81E-16
CR_00290W_A	Ortholog of S. cerevisiae Rts3	2.72	1.55E-30
C1_04590W_A	Protein of unknown function	2.68	1.21E-14
C1_04010C_A	Protein with a NADP-dependent oxidoreductase domain	2.67	1.06E-19
BMT7	Beta-mannosyltransferase	2.62	6.52E-10
PGA6	GPI-anchored cell wall adhesin-like protein	2.56	1.03E-07
C2_09810C_A	Protein with predicted peptidase domains	2.54	4.41E-09
ERG24	C-14 sterol reductase	2.46	3.16E-17
LPG20	Aldo-keto reductase family protein	2.44	4.30E-13
ECM331	GPI-anchored protein	2.43	5.60E-08
C6_00850W_A	Putative glutathione peroxidase	2.42	8.67E-10
GRE2	Putative reductase	2.40	1.85E-09
C2_07220W_A	Putative 20S proteasome assembly protein	2.35	1.15E-13
C1_13490C_A	Ortholog(s) have role in cellular calcium ion homeostasis	2.35	6.45E-24
GAL4	Zn(II)2Cys6 transcription factor	2.33	2.61E-13
GFA1	Glucosamine-6-phosphate synthase	2.32	1.27E-13
C1_11790W_A	Ortholog(s) have thymidylate kinase activity	2.31	2.34E-15
C1_09130W_A	Protein of unknown function; Spider biofilm induced	2.31	1.20E-13
CRH11	GPI-anchored cell wall transglycosylase	2.30	7.62E-08
TPM2	Putative tropomyosin isoform 2	2.30	1.53E-12
TUB1	Alpha-tubulin	2.29	1.78E-24
CHO1	Putative phosphatidylserine synthase	2.29	1.89E-10
CR_09040W_A	Protein of unknown function	2.19	5.77E-08
CR_04120C_A	Ortholog(s) have DNA-dependent ATPase activity	2.17	2.97E-12
C4_00860C_A	Protein of unknown function	2.15	4.41E-09

AHP1	Alkyl hydroperoxide reductase	2.15	8.33E-06
YKE2	Possible heterohexameric Gim/prefoldin protein complex subunit	2.14	5.22E-11
ERG1	Squalene epoxidase	2.13	1.33E-19
ERG10	Acetyl-CoA acetyltransferase	2.11	2.55E-08
C3_04740C_A	Ortholog(s) have DNA topoisomerase type I activity	2.11	5.34E-10
MLC1	Microtubule-dependent localized protein	2.09	4.07E-15
WOR4	Predicted C2H2 zinc finger protein	2.09	7.17E-12
ADH5	Putative alcohol dehydrogenase	2.09	4.57E-08
ERG3	C-5 sterol desaturase	2.07	1.24E-06
LAP41	Putative aminopeptidase yscI precursor	2.06	9.49E-09
RNR1	Ribonucleotide reductase large subunit	2.06	3.05E-13
ERG5	Putative C-22 sterol desaturase	2.05	1.93E-08
ERG26	C-3 sterol dehydrogenase	2.02	1.49E-11
INN1	Protein with similarity to *S. cerevisiae* Inn1p	2.02	2.40E-29
CRL1	Predicted GTPase of RHO family	2.00	6.85E-21
C5_03490C_A	Stationary phase enriched protein	1.99	1.06E-19
WOR2	Zn(II)2Cys6 transcription factor	1.99	8.15E-06
C6_04420W_A	Protein of unknown function	1.97	2.54E-06
C5_02850W_A	H/ACA box small nucleolar RNA (snoRNA)	1.96	5.29E-08
NIP100	p150 subunit of dynactin	1.95	4.09E-09
C1_09980C_A	Ortholog(s) have acylglycerol lipase activity, serine hydrolase activity	1.94	2.07E-06
CHS2	Chitin synthase	1.93	2.44E-25
ERG27	3-Keto sterol reductase	1.92	4.41E-09
FAV2	Adhesin-like protein	1.92	3.71E-06
C3_06860C_A	Putative xylose and arabinose reductase	1.90	4.78E-06
CR_09070C_A	Protein of unknown function	1.90	1.33E-10
CR_04870C_A	Trimethylaminobutyraldehyde dehydrogenase	1.89	1.27E-06
CR_04770C_A	Protein of unknown function	1.88	2.80E-11
CYB5	Cytochrome b(5)	1.88	1.42E-08
RRNL	Mitochondrial ribosomal RNA of the large ribosomal subunit	1.88	1.82E-06
C1_11720W_A	Ortholog of *Candida albicans* WO-1 : CAWG_00265	1.87	9.94E-06
NRG1	Transcription factor/repressor	1.86	1.93E-12
C1_11730W_A	Protein with SEL-1 like protein domain	1.86	5.87E-14
BMT3	Beta-mannosyltransferase	1.85	2.55E-13
C1_04470C_A	Protein of unknown function	1.84	5.84E-09
C3_03370C_A	Protein of unknown function	1.84	8.63E-12
SET3	NAD-dependent histone deacetylase	1.83	5.25E-17
C3_02870C_A	Protein of unknown function	1.82	3.69E-07
PHR2	Glycosidase	1.81	4.38E-12
ERG11	Lanosterol 14-alpha-demethylase	1.81	6.90E-06
REV3	Ortholog(s) have DNA-directed DNA polymerase activity	1.80	1.81E-11
C7_04060W_A	Protein of unknown function	1.79	2.80E-11
C1_04580C_A	Ortholog of Rmd6 involved in *S. cerevisiae* sporulation	1.79	9.66E-11
CEK1	ERK-family protein kinase	1.77	3.10E-10
ECM33	GPI-anchored cell wall protein	1.76	2.78E-10
IFD6	Aldo-keto reductase	1.75	6.62E-08
SVF1	Putative survival factor; stationary phase enriched protein	1.75	2.90E-14
BTS1	Putative geranylgeranyl diphosphate synthase	1.74	2.40E-15
SSO2	Plasma membrane t-SNARE	1.73	5.11E-23

DAP1	Similar to mammalian membrane-associated progesterone receptors	1.72	1.48E-14
GTT11	Glutathione S-transferase	1.72	6.33E-12
CR_02880W_A	Protein of unknown function	1.70	1.59E-08
STE13	Putative pheromone-processing dipeptidyl aminopeptidase	1.70	1.51E-12
ERG2	C-8 sterol isomerase	1.69	1.27E-07
C1_02440C_A	Ortholog(s) have thiol-dependent ubiquitin-specific protease activity	1.68	1.34E-11
PDI1	Putative protein disulfide-isomerase	1.68	8.24E-09
CHS8	Chitin synthase	1.66	3.45E-09
C1_04230W_A	Ortholog(s) have ubiquitin binding activity	1.65	2.57E-14
SLD1	Sphingolipid delta-8 desaturase	1.64	5.21E-10
YVC1	Putative vacuolar cation channel shock	1.64	6.64E-09
MSC7	role in reciprocal meiotic recombination	1.63	1.10E-28
SRR1	Two-component system response regulator	1.62	1.27E-06
SRB1	Essential GDP-mannose pyrophosphorylase	1.61	3.65E-07
PSD2	Ortholog(s) have phosphatidylserine decarboxylase activity	1.61	3.64E-15
C2_08620W_A	Protein of unknown function	1.57	3.03E-08
C1_13500C_A	H/ACA box small nucleolar RNA (snoRNA)	1.56	2.09E-06
CR_09330C_A	Protein with a Staphylococcal nuclease domain	1.53	1.77E-07
AHR1	Zn(II)2Cys6 transcription factor	1.53	7.61E-08
CDC12	Septin	1.52	1.53E-08
FAS2	Alpha subunit of fatty-acid synthase	1.52	4.39E-06
C6_04410C_A	Predicted alcohol dehydrogenase	1.52	2.67E-06
CDC3	Septin	1.50	1.20E-11
C5_04350C_A	Ortholog(s) have cell division site	1.50	2.35E-11
C4_06990W_A	Possible mannosyltransferase	1.49	2.25E-12
GCY1	Aldo/keto reductase	1.48	2.48E-06
C2_09480W_A	Ortholog(s) have DNA-directed DNA polymerase activity	1.47	8.79E-10
RAD50	Putative DNA double-strand break repair factor	1.46	3.48E-09
C1_11120C_A	Protein similar to *S. cerevisiae* Gvp36p	1.43	1.20E-13
FBP1	Fructose-1,6-bisphosphatase	1.43	6.89E-07
PDS5	Putative protein with a predicted role in establishment and maintenance of sister chromatid condensation and cohesion	1.42	5.46E-07
BFR1	Protein involved in the maintenance of normal ploidy	1.42	1.08E-07
CR_02300C_A	Protein with a Vps9 vacuolar protein sorting protein domain	1.41	4.76E-12
PMM1	Phosphomannomutase	1.41	3.08E-07
MPT5	Putative RNA-binding protein	1.38	3.14E-12
C5_02520W_A	Protein of unknown function	1.38	1.23E-06
C2_05720C_A	Putative oxysterol binding protein family	1.37	2.34E-15
C6_02490C_A	Protein of unknown function	1.37	8.34E-07
SFH5	Putative phosphatidylinositol transporter	1.36	1.93E-07
LEU4	Putative 2-isopropylmalate synthase	1.35	9.85E-06
C1_11250W_A	Microtubule-binding protein of the cortical microtubule	1.35	2.37E-08
CDC13	Essential protein with similarity to S. cerevisiae Cdc13p	1.35	3.69E-07
MYO1	Component of actomyosin ring at neck of newly-emerged bud	1.34	3.05E-13
PRE10	Alpha7 (C8) subunit of the 20S proteasome	1.34	2.15E-11
C4_05810W_A	Ortholog of *S. cerevisiae* : CAJ1	1.34	9.29E-06
C2_01750C_A	Protein with a life-span regulatory factor domain	1.33	8.99E-07
KTR4	Mannosyltransferase	1.31	1.31E-07
CHS5	Putative chitin biosynthesis protein	1.30	1.28E-06

RGS2	Protein of RGS superfamily	1.29	1.10E-07
SCT2	Putative glycerol-3-phosphate acyltransferase	1.28	5.27E-07
VPS1	Dynamin-family GTPase-related protein	1.28	1.76E-09
CR_08430W_A	Protein similar to S. cerevisiae Vac14p	1.27	3.94E-13
GIG1	Protein induced by N-acetylglucosamine (GlcNAc)	1.27	7.65E-07
C7_04210C_A	Protein similar to Aspergillus CYSK O-acetylserine sulfhydrylase	1.27	2.95E-08
C1_07260C_A	Ortholog(s) have DNA replication origin binding, single-stranded DNA binding activity	1.26	1.83E-16
C5_00750C_A	Predicted protein tyrosine phosphatase	1.26	4.25E-10
BCR1	Transcription factor	1.26	3.25E-07
RDH54	Putative DNA-dependent ATPase with a predicted role in DNA recombination and repair	1.25	4.83E-08
IML2	Protein of unknown function	1.25	4.59E-08
SEN1	Putative helicase	1.25	1.62E-08
C6_00890W_A	Ortholog(s) have cytoplasm localization	1.25	4.03E-08
C5_01090C_A	Planktonic growth-induced gene	1.24	7.45E-07
USO6	Putative vesicular transport protein	1.24	2.74E-09
SCL1	Proteasome subunit YC7alpha	1.23	2.36E-06
C4_06740C_A	Ortholog(s) have protein binding, bridging activity	1.21	1.53E-08
NPT1	Putative nicotinate phosphoribosyltransferase	1.19	3.00E-10
C5_02050W_A	Ortholog(s) have role in Golgi to endosome transport,	1.18	5.03E-10
RAD7	Protein similar to S. cerevisiae Rad7p	1.18	3.49E-09
C1_01300W_A	Putative thioredoxin	1.17	2.98E-09
C3_07770C_A	Ortholog(s) have microtubule binding, protein homodimerization activity	1.17	3.48E-08
C4_04750W_A	Ortholog(s) have role in negative regulation of gluconeogenesis	1.16	1.38E-09
NCP1	NADPH-cytochrome P450 reductase	1.15	1.09E-07
CCH1	Voltage-gated Ca2+ channel of the high affinity calcium uptake system	1.15	1.86E-11
C4_01220C_A	Protein with a glycoside hydrolase domain	1.14	1.05E-06
RTA2	Flippase	1.14	5.45E-10
C1_10730W_A	Putative protein of unknown function	1.14	5.13E-06
BET2	Putative Type II geranylgeranyltransferase beta subunit	1.13	2.11E-07
C2_02020W_A	Ortholog(s) have Lys48-specific deubiquitinase activity	1.12	1.05E-06
CR_04080C_A	Endoplasmic reticulum (ER) protein-translocation complex subunit	1.11	7.29E-09
C4_05800C_A	Protein of unknown function	1.11	3.07E-06
C3_03430C_A	Ortholog(s) have role in ATP export	1.10	4.80E-08
C2_00230W_A	Ortholog(s) have arginyltransferase activity	1.09	2.34E-06
DYN1	Dynein heavy chain	1.07	3.48E-09
CR_05890C_A	Ortholog(s) have acetyltransferase activator activity, histone binding activity and role in double-strand break repair	1.07	5.11E-08
C6_02810C_A	Ortholog of *C. dubliniensis CD36*: Cd36_63300	1.07	9.48E-06
PMC1	Vacuolar calcium P-type ATPase	1.06	1.76E-09
PRE6	Putative alpha-4 subunit of the proteasome	1.05	1.53E-06
C4_01790W_A	Adapter protein for pexophagy and the cytoplasm-to-vacuole targeting (Cvt) pathway	1.05	1.84E-08
HEM2	Putative porphobilinogen synthase	1.04	8.71E-07
C1_02770W_A	Protein of unknown function	1.03	6.84E-06
C4_01670C_A	Ortholog(s) have role in TOR signaling	1.03	5.67E-07
ARO80	Zn(II)2Cys6 transcription factor	1.02	5.14E-09
SSN8	Ortholog of S. cerevisiae Ssn8; a component of RNA polymerase II holoenzyme	1.01	5.40E-07

RFA1	Putative DNA replication factor A	0.99	4.01E-07
VPS36	ESCRT II protein sorting complex subunit	0.97	2.85E-06
MKC1	MAP kinase	0.97	6.90E-06
PSD1	Phosphatidylserine decarboxylase	0.97	8.67E-08
C2_07010W_A	Predicted ER protein involved in ER-nucleus signaling	0.94	2.67E-06
UBP6	Putative ubiquitin-specific protease of the 26S proteasome	0.93	9.04E-06
C3_07230W_A	Aminophospholipid translocase (flippase)	0.92	2.56E-09
COQ6	Ortholog(s) have oxidoreductase activity	0.92	1.46E-06
AKL1	Putative serine/threonine protein kinase	0.91	1.86E-07
C4_06020C_A	Ortholog(s) have DNA-directed DNA polymerase activity	0.90	1.33E-06
C4_04090C_A	Calnexin	0.88	1.49E-06
C3_06710W_A	Protein of unknown function	0.88	9.45E-07
C1_03330C_A	Ortholog(s) have phosphatidylinositol-3,5-bisphosphate binding activity a	0.88	1.81E-08
APN1	Ortholog of S. cerevisiae Apn1; an AP endonuclease	0.86	6.60E-10
CR_10230W_A	Ortholog(s) have histone acetyltransferase activity	0.85	3.82E-06
ZNC1	Zn(2)-Cys(6) transcription factor	0.81	3.14E-06
C2_00570W_A	Ortholog(s) have DNA translocase activit	-0.45	9.04E-06
IML1	Putative protein with a role in autophagy	-0.81	1.09E-06
C1_11300C_A	Ortholog(s) have RNA binding	-0.84	4.13E-09
ZCF10	Putative transcription factor with zinc cluster DNA-binding motif	-0.86	5.25E-07
C3_03820C_A	Ortholog(s) have role in protein insertion into mitochondrial membrane from inner side	-0.86	1.38E-09
C6_01000C_A	Ortholog(s) have translation regulator activity	-0.87	1.86E-07
C4_04130W_A	Ortholog(s) have role in mitochondrial respiratory chain complex IV assembly	-0.89	8.48E-06
C4_03780C_A	Putative mitochondrial membrane protein	-0.90	1.12E-06
C1_13010W_A	Ortholog(s) have mRNA binding, translation regulator activity	-0.90	4.42E-06
C6_03530C_A	Predicted ORF from Assembly 19	-0.91	2.49E-06
C1_14200W_A	Possible Golgi membrane protein	-0.97	7.96E-06
CR_03120W_A	Mitochondrial intermembrane space protein of unknown function	-1.02	5.08E-06
TSC2	Putative GTPase-activating protein	-1.02	9.65E-08
C5_00820W_A	Ortholog(s) have structural constituent of ribosome activity	-1.04	2.03E-06
AVT1	Putative vacuolar transporter	-1.05	1.35E-06
C1_05210C_A	Protein of unknown function	-1.08	2.60E-06
C6_00390W_A	Ortholog(s) have serine-tRNA ligase activity	-1.08	6.54E-10
ARF1	ADP-ribosylation factor	-1.08	1.47E-08
C4_00820W_A	Ortholog(s) have magnesium ion transmembrane transporter activity	-1.09	9.29E-06
HIP1	Amino acid permeases	-1.10	1.42E-07
MRPS9	Mitochondrial ribosomal protein S9	-1.11	9.71E-06
C2_03560C_A	Ortholog(s) have structural constituent of ribosome activity	-1.11	3.29E-06
CR_01370C_A	Ortholog(s) have structural constituent of ribosome activity	-1.13	1.25E-06
C2_07190C_A	Putative mitochondrial ribosomal protein	-1.14	2.29E-07
MIF2	Centromere-associated protein	-1.14	4.89E-12
IFM1	Putative mitochondrial translation initiation factor	-1.14	2.19E-07
C7_01610W_A	Ortholog(s) have role in mitochondrial respiratory chain complex IV assembly	-1.15	2.64E-06
CR_07760W_A	Ortholog(s) have structural constituent of ribosome activity	-1.17	3.73E-08
C6_03730C_A	Ortholog(s) have structural constituent of ribosome activity	-1.18	1.23E-06
C7_01720W_A	Flavin-linked sulfhydryl oxidase	-1.19	1.82E-12
C5_03980W_A	Protein of unknown function	-1.21	6.26E-07

C5_04530W_A	Putative mitochondrial ribosomal protein of the small subunit	-1.21	2.33E-06
RIM9	Protein required for alkaline pH response via the Rim101 signaling pathway	-1.21	5.14E-07
TIM23	Protein involved in mitochondrial matrix protein import	-1.22	3.85E-08
CR_01100C_A	Ortholog(s) have role in mitochondrial respiratory chain complex assembly	-1.22	1.17E-07
CWC22	Predicted spliceosome-associated protein	-1.23	1.85E-08
RSN1	Protein of unknown function	-1.23	8.26E-08
CR_03110W_A	Ortholog(s) have role in mitochondrial translation	-1.24	2.89E-09
C4_04820C_A	Ortholog(s) have structural constituent of ribosome activity	-1.26	2.47E-06
C1_14330W_A	Ortholog(s) have tRNA-5-taurinomethyluridine 2-sulfurtransferase activity	-1.28	2.37E-07
C6_03200W_A	Ortholog of *C. dubliniensis* CD36 : Cd36_63860	-1.28	1.21E-06
ALS9	ALS family cell-surface glycoprotein	-1.29	1.60E-09
CDR11	Putative transporter of PDR subfamily of ABC family	-1.30	8.34E-07
MRPL3	Ribosomal protein of the large subunit	-1.32	2.87E-06
SSC1	Heat shock protein	-1.35	8.52E-08
C6_03440W_A	Ortholog(s) have rRNA binding activity	-1.38	2.54E-07
C1_14190C_A	Protein phosphatase inhibitor	-1.38	3.80E-06
MGE1	Putative mitochondrial matrix cochaperone;	-1.39	4.76E-06
C1_01150C_A	Ortholog(s) have tRNA methyltransferase activity	-1.39	1.79E-06
SSY1	Amino acid sensor	-1.40	7.56E-06
GAP1	Amino acid permease	-1.41	1.47E-08
C3_01850W_A	Ortholog(s) have structural constituent of ribosome activity	-1.43	5.98E-07
CDR4	Putative ABC transporter superfamily	-1.43	2.18E-08
C4_00810C_A	Ortholog(s) have serine-type endopeptidase activity	-1.46	2.77E-06
C7_01590C_A	Ortholog of C. dubliniensis CD36 : Cd36_71420	-1.46	6.19E-10
C1_08520C_A	Ortholog(s) have structural constituent of ribosome activity	-1.46	2.05E-09
C1_13060C_A	Ortholog(s) have role in mitochondrial translation	-1.48	3.00E-06
C5_02740W_A	Ortholog(s) have oxidoreductase activity	-1.48	2.39E-12
C1_06350W_A	Protein of unknown function	-1.48	2.56E-09
C4_03410W_A	Ortholog(s) have structural constituent of ribosome activity	-1.49	1.08E-12
ZCF29	Zn(II)2Cys6 transcription factor	-1.49	4.80E-11
CAL0000190891	Predicted MFS family membrane transporter	-1.49	9.80E-06
CHA1	Similar to catabolic ser/thr dehydratases	-1.49	2.66E-06
MRPL10	Putative mitochondrial large subunit ribosomal protein	-1.49	1.86E-08
MNN22	Alpha-1,2-mannosyltransferase	-1.57	4.60E-11
C6_03540W_A	Ortholog(s) have structural constituent of ribosome activity	-1.58	5.35E-14
C2_04790C_A	Predicted membrane transporter	-1.59	2.45E-07
C2_00480C_A	Ortholog(s) have structural constituent of ribosome activity	-1.61	8.76E-10
PHHB	Putative 4a-hydroxytetrahydrobiopterin dehydratase	-1.62	5.69E-07
TUF1	Translation elongation factor TU	-1.63	3.73E-08
PLB3	GPI-anchored cell surface phospholipase B	-1.64	6.05E-06
RIM101	Transcription factor	-1.64	4.41E-09
C2_02180W_A	Ortholog of *S. cerevisiae* Zrt3, vacuolar membrane zinc transporter	-1.65	5.08E-15
MSW1	Protein similar to mitochondrial tryptophanyl-tRNA synthetase	-1.68	1.14E-16
C7_01510W_A	Predicted membrane transporter	-1.71	4.81E-07
C4_07140W_A	Predicted mitochondrial intermembrane space protein	-1.73	4.39E-07
GIT3	Glycerophosphocholine permease	-1.74	2.97E-07
IFR2	Zinc-binding dehydrogenase	-1.79	2.02E-08

C1_06840C_A	Ortholog(s) have role in mitochondrial respiratory chain complex IV assembly	-1.82	7.14E-07
ENA2	Putative sodium transporter	-1.82	5.21E-10
CTR1	Copper transporter	-1.84	3.47E-07
SKN1	Protein with a role in beta-1,6-glucan synthesis;	-1.91	2.04E-06
CAN3	Predicted amino acid transmembrane transporter	-1.91	5.76E-11
C3_00840C_A	Protein of unknown function	-1.95	3.44E-08
RME1	Zinc finger protein	-1.95	4.98E-07
FCY21	High affinity, high capacity, hypoxanthine-adenine-guanine-cytosine/H+ symporter	-1.98	2.47E-06
C3_01260C_A	Putative adhesin-like protein	-1.99	3.88E-09
ENA21	Predicted P-type ATPase sodium pump	-2.00	1.19E-09
ALP1	Cystine transporter	-2.13	3.95E-07
COX15	Cytochrome oxidase assembly protein	-2.15	7.72E-11
ATX1	Putative cytosolic copper metallochaperone	-2.22	8.01E-10
C1_08350C_A	Ortholog of *C. dubliniensis* CD36: Cd36_02630	-2.28	7.27E-13
CR_08880C_A	Protein of unknown function	-2.59	1.83E-16
AOX2	Alternative oxidase	-2.70	2.10E-07
CRP1	Copper transporter	-2.73	4.16E-13
SIT1	Transporter of ferrichrome siderophores, not ferrioxamine B	-2.83	1.53E-09
C7_03560W_A	Protein of unknown function	-2.98	1.34E-27
TPO3	Putative polyamine transporter; MFS-MDR family	-2.99	9.59E-16
DLD1	Putative D-lactate dehydrogenase	-3.00	7.03E-16
CTN1	Carnitine acetyl transferase	-3.00	1.57E-11
FMP45	Predicted membrane protein induced during mating	-3.01	9.17E-10
C1_07160C_A	Protein conserved among the CTG-clade	-3.19	3.11E-12
tR(UCU)4	tRNA-Arg, predicted by tRNAscan-SE; UCU anticodon	-3.25	7.34E-09
GIT2	Putative glycerophosphoinositol permease	-3.34	1.89E-24
HSP30	Putative heat shock protein	-3.36	2.98E-11
C1_11320C_A	Protein of unknown function	-3.48	4.80E-18
PHO89	Putative phosphate permease	-3.59	1.28E-07
ZRT2	Zinc transporter	-3.78	1.92E-23
C5_04980W_A	Putative adhesin-like protein	-4.13	1.60E-24
PHO84	High-affinity phosphate transporter	-4.29	1.81E-17
HNM3	Putative transporter	-4.30	3.60E-20
PRA1	Cell surface protein that sequesters zinc from host tissue	-4.40	1.51E-12
JEN2	Dicarboxylic acid transporter	-5.34	3.80E-47

Supplementary Table S3: Gene expression data; Significantly upregulated/downregulated genes in *C. albicans* treated with fluconazole compared to untreated controls. The genes that are known to be associated with antimicrobial sensitivity/resistance are shaded in light blue (adjusted *p-value* < $1e^{-5}$).

Name	Annotation	log2 Fold Change	Adjusted P value
ADH3	Putative NAD-dependent (R,R)-butanediol dehydrogenase	3.25	2.36E-08
C3_03460C_A	Protein of unknown function	3.19	4.58E-20
IFD6	Aldo-keto reductase	3.02	1.11E-21
ATX1	Putative cytosolic copper metallochaperone	2.75	2.42E-14
GRP2	Methylglyoxal reductase	2.62	8.07E-08
C3_05450C_A	Protein of unknown function	2.53	2.17E-11
CRZ2	C2H2 transcription factor	2.51	2.86E-17
HSP104	Heat-shock protein	2.47	3.99E-08
C1_01510W_A	Protein of unknown function	2.46	2.65E-12
WOR4	Predicted C2H2 zinc finger protein	2.40	1.18E-14
UGT51C1	UDP-glucose:sterol glucosyltransferase	2.38	1.45E-22
CR_09100C_A	Aldo-keto reductase	2.35	1.36E-09
C7_04090C_A	Predicted mitochondrial cardiolipin-specific phospholipase	2.35	1.59E-10
SRR1	Two-component system response regulator	2.24	1.98E-11
HSP78	Heat-shock protein	2.22	1.27E-08
AAF1	Possible regulatory protein	2.18	2.21E-08
NRG1	Transcription factor/repressor	2.18	4.02E-16
RME1	Zinc finger protein	2.17	3.28E-08
OPT3	Oligopeptide transporter	2.15	1.60E-10
C5_04030W_A	Protein of unknown function	2.08	3.99E-08
GAL102	UDP-glucose 4,6-dehydratase	2.05	3.84E-08
ERO1	Ortholog of *S. cerevisiae* Ero1	2.00	1.82E-08
YIM1	Protein similar to protease of mitochondrial inner membrane;	2.00	2.04E-08
GOR1	Ortholog(s) have glyoxylate reductase activity	2.00	2.37E-07
CR_06140W_A	Protein of unknown function	1.98	1.08E-08
EFG1	bHLH transcription factor	1.97	9.31E-10
C1_03990W_A	Ortholog(s) have proteasome binding activity	1.95	2.07E-08
SIS1	Putative Type II HSP40 co-chaperone	1.94	5.25E-06
RFG1	HMG domain transcriptional repressor of filamentous growth and hyphal genes	1.94	3.12E-11
INO2	Transcriptional activator	1.92	1.24E-08
C3_00360W_A	Protein of unknown function	1.88	2.21E-10
CSH1	Aldo-keto reductase	1.87	5.61E-07
HSP90	Essential chaperone	1.85	2.86E-07
C7_00770W_A	Protein of unknown function	1.81	6.81E-06
STI1	Protein that interacts with Cdc37 and Crk1 in two-hybrid; may be involved in Cdc37 chaperone activity;	1.74	7.75E-06
CDR4	Putative ABC transporter superfamily	1.70	6.09E-11
MOH1	Ortholog of *S. cerevisiae* Moh1, essential for staionary phase growth	1.68	2.64E-10
C1_04010C_A	Protein with a NADP-dependent oxidoreductase domain	1.68	1.47E-07
C6_00290W_A	Protein of unknown function	1.67	2.04E-08
ADA2	Zinc finger and homeodomain transcriptional coactivator	1.67	2.43E-13
CUP9	Transcription factor	1.66	7.42E-07
LPG20	Aldo-keto reductase family protein	1.65	8.38E-06
ALK2	N-Alkane inducible cytochrome P450	1.63	1.09E-09
C6_00920W_A	Putative mitochondrial intermembrane space protein	1.61	4.02E-08

ZCF1	Zn(II)2Cys6 transcription factor	1.59	2.33E-12
C2_02920W_A	Putative adhesin-like protein	1.58	6.18E-07
PGA52	GPI-anchored cell surface protein of unknown function	1.57	3.79E-06
SNQ2	Protein similar to S. cerevisiae Snq2p transporter	1.55	6.73E-07
ISA1	Putative mitochondrial iron-sulfur protein	1.55	1.62E-08
C4_02740W_A	Protein of unknown function	1.54	2.80E-06
C2_01750C_A	Protein with a life-span regulatory factor domain	1.51	4.57E-08
AXL1	Putative endoprotease	1.50	8.38E-06
C1_09210C_A	Putative transporter	1.47	8.96E-08
C2_03690C_A	Protein of unknown function	1.43	6.88E-10
C2_09880C_A	Putative protein of unknown function	1.42	7.26E-06
C3_02630C_A	Protein of unknown function	1.42	1.29E-08
YOR1	Protein similar to *S. cerevisiae* Yor1	1.41	2.33E-12
HAL9	Putative Zn(II)2Cys6 transcription factor	1.40	5.22E-08
RPN4	C2H2 transcription factor	1.40	3.13E-09
ZCF39	Zn(II)2Cys6 transcription factor	1.39	1.55E-06
RGS2	Protein of RGS superfamily	1.39	3.28E-08
CR_10230W_A	Ortholog(s) have histone acetyltransferase activity	1.36	2.94E-14
CR_06960W_A	Ortholog(s) have ATP binding, DNA replication origin binding activity	1.35	1.73E-06
C6_00110C_A	Ortholog of C. dubliniensis CD36 : Cd36_60130	1.29	1.88E-07
CR_00040C_A	Putative adhesin-like protein	1.28	1.01E-06
CR_07480W_A	A predicted auxin family transmembrane transporter	1.22	2.41E-08
BCR1	Transcription factor	1.21	3.28E-06
C4_03020W_A	Putative mitochondrial GTPase	1.18	4.87E-09
CR_05860W_A	Protein of unknown function	1.16	4.22E-06
ALS7	ALS family protein	1.12	1.07E-08
C1_01130W_A	Putative ubiquitin ligase complex component	1.09	2.80E-06
MHP1	Protein similar to *S. cerevisiae* Mhp1p	1.09	4.69E-06
ARD	D-arabitol dehydrogenase	1.09	6.87E-06
C3_04330C_A	Protein of unknown function	1.07	8.47E-06
TEL1	Ortholog(s) have protein serine/threonine kinase activity	-0.85	1.49E-07
FBA1	Fructose-bisphosphate aldolase	-1.64	8.92E-08
PHHB	Putative 4a-hydroxytetrahydrobiopterin dehydratase	-1.72	2.87E-07
IFR2	Zinc-binding dehydrogenase	-1.78	8.07E-08
MNN12	Predicted alpha-1,3-mannosyltransferase activity	-1.93	1.55E-06
CRH11	GPI-anchored cell wall transglycosylase	-2.36	1.36E-07
SOD5	Cu-containing superoxide dismutase	-3.26	4.51E-08
C6_02100W_A	Secreted protein	-3.54	9.31E-10

Supplementary Table S4: Gene expression data; Significantly upregulated/downregulated genes in *C. albicans* treated with C12AHL compared to untreated controls. The genes that are known to be associated with antimicrobial sensitivity/resistance are shaded in light blue (adjusted *p-value* < $1e^{-5}$).

Name	Annotation	log2 Fold Change	Adjusted P value
C3_03460C_A	Protein of unknown function	3.45	3.54E-23
ADH3	Putative NAD-dependent (R,R)-butanediol dehydrogenase	3.05	2.81E-07
IFD6	Aldo-keto reductase	3.00	2.14E-21
ATX1	Putative cytosolic copper metallochaperone	2.74	3.92E-14
HSP104	Heat-shock protein	2.54	1.83E-08
GRP2	Methylglyoxal reductase	2.50	4.86E-07
C1_01510W_A	Protein of unknown function	2.41	1.41E-11
C7_00350C_A	Protein of unknown function	2.39	4.64E-06
C3_05450C_A	Protein of unknown function	2.38	4.54E-10
C7_04090C_A	Predicted mitochondrial cardiolipin-specific phospholipase	2.33	2.33E-10
CRZ2	C2H2 transcription factor	2.30	2.41E-14
CR_09100C_A	Aldo-keto reductase	2.30	4.70E-09
HSP78	Heat-shock protein	2.23	1.33E-08
UGT51C1	UDP-glucose:sterol glucosyltransferase	2.20	1.95E-19
C7_00770W_A	Protein of unknown function	2.19	1.73E-08
OPT3	Oligopeptide transporter	2.16	1.39E-10
GOR1	Ortholog(s) have glyoxylate reductase activity	2.16	1.86E-08
SRR1	Two-component system response regulator	2.15	1.81E-10
AAF1	Possible regulatory protein	2.13	5.63E-08
WOR4	Predicted C2H2 zinc finger protein	2.08	6.53E-11
RME1	Zinc finger protein	1.98	9.00E-07
SIS1	Putative Type II HSP40 co-chaperone	1.98	3.87E-06
CR_06140W_A	Protein of unknown function	1.96	1.56E-08
NRG1	Transcription factor/repressor	1.95	9.01E-13
YIM1	Protein similar to protease of mitochondrial inner membrane	1.95	6.00E-08
ALK2	N-Alkane inducible cytochrome P450	1.93	2.75E-13
CSH1	Aldo-keto reductase	1.91	3.78E-07
ERO1	Ortholog of *S. cerevisiae* Ero1	1.91	1.20E-07
EFG1	bHLH transcription factor	1.88	7.64E-09
C5_04030W_A	Protein of unknown function	1.85	2.15E-06
GAL102	UDP-glucose 4,6-dehydratase	1.85	1.54E-06
RFG1	HMG domain transcriptional repressor of filamentous growth and hyphal genes	1.81	7.32E-10
CDR4	Putative ABC transporter superfamily	1.76	1.68E-11
INO2	Transcriptional activator that forms a heterodimer with Ino4p	1.76	3.13E-07
SNQ2	Protein similar to S. cerevisiae Snq2p transporter	1.74	1.56E-08
C1_09210C_A	Putative transporter	1.72	2.42E-10
LPG20	Aldo-keto reductase family protein	1.71	3.87E-06
HSP90	Essential chaperone	1.71	3.87E-06
C1_03990W_A	Ortholog(s) have proteasome binding activity	1.64	6.48E-06
MOH1	Ortholog of *S. cerevisiae* Moh1	1.61	2.14E-09
C1_04010C_A	Protein with a NADP-dependent oxidoreductase domain	1.57	1.54E-06
C6_00290W_A	Protein of unknown function	1.54	4.63E-07
ADA2	Zinc finger and homeodomain transcriptional coactivator	1.51	8.98E-11
C4_02740W_A	Protein of unknown function	1.48	9.33E-06
C2_00770W_A	Putative protein of unknown function	1.47	9.00E-07

C3_00360W_A	Protein of unknown function	1.43	4.78E-06
ZCF1	Zn(II)2Cys6 transcription factor	1.43	6.29E-10
ISA1	Putative mitochondrial iron-sulfur protein	1.39	7.93E-07
YOR1	Protein similar to *S. cerevisiae* Yor1	1.35	3.45E-11
HAL9	Putative Zn(II)2Cys6 transcription factor	1.35	2.48E-07
RPN4	C2H2 transcription factor	1.34	1.56E-08
CR_06960W_A	Ortholog(s) have ATP binding, DNA replication origin binding activity	1.29	6.48E-06
C2_03690C_A	Protein of unknown function	1.25	1.48E-07
C3_02630C_A	Protein of unknown function	1.20	3.87E-06
RGS2	Protein of RGS superfamily	1.17	7.97E-06
MHP1	Protein similar to *S. cerevisiae* Mhp1p	1.10	3.93E-06
CR_10230W_A	Ortholog(s) have histone acetyltransferase activity	1.07	8.15E-09
C1_01130W_A	Putative ubiquitin ligase complex component	1.06	7.23E-06
CR_07480W_A	A predicted auxin family transmembrane transporter	1.02	9.33E-06
C4_03020W_A	Putative mitochondrial GTPase	0.98	2.64E-06
ALS7	ALS family protein	0.95	2.83E-06
HOL4	Putative ion transporter	-1.24	1.54E-06
ENA21	Predicted P-type ATPase sodium pump	-1.59	9.11E-06
PHHB	Putative 4a-hydroxytetrahydrobiopterin dehydratase	-1.78	1.04E-07
FBA1	Fructose-bisphosphate aldolase	-1.79	5.31E-09
IFR2	Zinc-binding dehydrogenase	-1.82	4.01E-08
PHR1	Cell surface glycosidase	-1.84	3.61E-06
MNN12	Predicted alpha-1,3-mannosyltransferase	-1.90	2.89E-06
C6_02100W_A	Secreted protein	-3.73	1.15E-10

Supplementary Table S5: Gene expression data; Significantly upregulated/downregulated genes in *C. albicans* treated with C12AHL+fluconazole compared to untreated controls. The genes that are known to be associated with antimicrobial sensitivity/resistance are shaded in light blue (adjusted *p-value* < $1e^{-5}$).

Name	Annotation	log2 Fold Change	Adjusted P value
JEN2	Dicarboxylic acid transporter	5.50	3.54E-50
ATX1	Putative cytosolic copper metallochaperone	4.97	2.48E-48
RME1	Zinc finger protein	4.13	4.48E-30
CRP1	Copper transporter	3.86	3.57E-26
HSP30	Putative heat shock protein	3.76	4.82E-14
PHO84	High-affinity phosphate transporter	3.73	1.68E-13
ADH3	Putative NAD-dependent (R,R)-butanediol dehydrogenase	3.71	2.22E-11
TPO3	Putative polyamine transporter	3.58	1.37E-22
OPT3	Oligopeptide transporter	3.57	3.50E-30
C5_04980W_A	Putative adhesin-like protein	3.51	5.28E-18
IFE2	Putative alcohol dehydrogenase	3.49	4.57E-13
C7_03560W_A	Protein of unknown function	3.47	4.79E-38
FCY21	High affinity, high capacity, hypoxanthine-adenine-guanine-cytosine/H+ symporter	3.41	6.13E-18
HSP104	Heat-shock protein	3.34	2.05E-15
HSP78	Heat-shock protein	3.32	4.07E-20
HNM3	Putative transporter	3.32	2.84E-12
CTN1	Carnitine acetyl transferase	3.30	5.56E-14
UGT51C1	UDP-glucose: sterol glucosyltransferase	3.21	4.24E-43
ZRT2	Zinc transporter	3.17	1.07E-16
C1_11320C_A	Protein of unknown function	3.16	4.80E-15
CDR4	Putative ABC transporter superfamily	3.13	5.04E-39
C4_07140W_A	Predicted mitochondrial intermembrane space protein	3.10	7.70E-22
ALP1	Cystine transporter	2.96	2.66E-13
SSA2	HSP70 family chaperone	2.95	6.26E-10
GCV2	Glycine decarboxylase P subunit	2.90	8.28E-11
C7_00350C_A	Protein of unknown function	2.88	1.78E-09
SIT1	Transporter of ferrichrome siderophores	2.87	6.07E-10
DLD1	Putative D-lactate dehydrogenase	2.79	6.07E-14
FMP45	Predicted membrane protein induced during mating	2.71	3.36E-08
MDH1-1	Predicted malate dehydrogenase precursor	2.67	5.83E-07
HSP21	Small heat shock protein	2.65	5.86E-08
CAN1	Basic amino acid permease	2.65	1.81E-08
C6_00810C_A	Protein of unknown function	2.47	3.77E-08
CR_08880C_A	Protein of unknown function	2.47	3.83E-15
CYS3	Cystathionine gamma-lyase; induced by alkaline, amphotericin B	2.46	6.06E-06
C1_08350C_A	Ortholog of C. dubliniensis CD36: Cd36_02630	2.38	3.96E-14
ELF1	Putative mRNA export protein	2.37	6.55E-06
MUP1	Putative high affinity methionine permease	2.35	2.37E-08

C1_07160C_A	Protein conserved among the CTG-clade	2.35	4.40E-07
GIT3	Glycerophosphocholine permease	2.35	8.31E-13
C2_03110W_A	Protein of unknown function	2.33	9.30E-11
C7_01170C_A	Putative oxidoreductase	2.28	1.25E-06
GNP1	Similar to asparagine and glutamine permease	2.26	5.40E-16
SSC1	Heat shock protein	2.25	4.72E-21
tV(AAC)4	tRNA-Val	2.24	4.45E-07
C7_04090C_A	Predicted mitochondrial cardiolipin-specific phospholipase	2.24	1.99E-10
ARG1	Argininosuccinate synthase	2.23	7.64E-08
CTA4	Zn(II)2Cys6 transcription factor	2.23	4.72E-11
AQY1	Aquaporin water channel	2.22	1.60E-08
C6_00290W_A	Protein of unknown function	2.21	2.13E-15
C6_03180C_A	Ortholog(s) have protein binding, bridging, ubiquitin protein ligase binding activity	2.20	4.99E-09
C2_00760C_A	Protein of unknown function	2.20	8.21E-11
CDR11	Putative transporter of PDR subfamily	2.18	3.82E-18
RPS27A	Ribosomal protein S27	2.16	4.11E-09
GRF10	Putative homeodomain transcription factor	2.11	7.64E-10
NDT80	Meiosis-specific transcription factor	2.11	3.13E-12
ALR1	Putative transporter of divalent cations	2.09	1.58E-06
C1_13820C_A	Protein of unknown function	2.08	3.87E-09
KGD1	Putative 2-oxoglutarate dehydrogenase	2.08	7.55E-10
SIS1	Putative Type II HSP40 co-chaperone	2.06	2.22E-07
DED1	Predicted ATP-dependent RNA helicase	2.05	2.26E-09
PHO87	Putative phosphate permease	2.05	6.46E-09
CPH2	Myc-bHLH transcription factor	2.04	7.55E-17
C7_00880C_A	Protein of unknown function	2.02	1.97E-10
PTR22	Oligopeptide transporter	2.01	4.78E-08
SUL2	Putative sulfate transporter	1.97	6.15E-10
CR_08420W_A	Putative DnaJ-like heat shock/chaperone	1.96	4.04E-06
C2_08100W_A	Mitochondrial apoptosis-inducing factor	1.95	2.05E-06
MET3	ATP sulfurylase	1.94	7.42E-06
C2_06440C_A	Protein with homology to peroxisomal membrane proteins	1.93	2.60E-06
COX15	Cytochrome oxidase assembly protein	1.93	4.99E-09
ARD	D-arabitol dehydrogenase	1.92	7.27E-19
ALS7	ALS family protein	1.92	1.57E-26
C1_01130W_A	Putative ubiquitin ligase complex component	1.91	1.06E-19
C7_03590C_A	Putative member of the multi-drug and toxin extrusion (MATE) family	1.90	1.44E-06
CR_06960W_A	Ortholog(s) have ATP binding, DNA replication origin binding activity	1.90	3.68E-13
GOR1	Ortholog(s) have glyoxylate reductase activity	1.90	2.97E-07
CAN3	Predicted amino acid transmembrane transporter	1.89	8.06E-11

HSP90	Essential chaperone	1.86	5.25E-08
STI1	Protein that interacts with Cdc37 and Crk1 in two-hybrid	1.86	2.42E-07
MNN22	Alpha-1,2-mannosyltransferase	1.86	2.25E-15
SNQ2	Protein similar to S. cerevisiae Snq2p transporter	1.84	2.49E-10
MET2	Homoserine acetyltransferase	1.84	1.41E-07
ITR1	MFS inositol transporter	1.83	1.68E-09
ALS1	Cell-surface adhesin	1.83	4.55E-08
RIM101	Transcription factor	1.80	5.30E-11
PLB3	GPI-anchored cell surface phospholipase B	1.80	3.10E-07
C4_05900C_A	Protein of unknown function	1.80	2.82E-12
ROA1	Putative PDR-subfamily ABC transporter	1.79	9.99E-09
CR_07170W_A	Possible dehydrogenase	1.78	3.28E-09
C6_03200W_A	Ortholog of C. dubliniensis CD36 : Cd36_63860	1.78	2.23E-12
GIT2	Putative glycerophosphoinositol permease	1.77	2.36E-07
PDE2	High affinity cyclic nucleotide phosphodiesterase	1.77	3.53E-10
C3_01260C_A	Putative adhesin-like protein	1.76	2.13E-07
TUF1	Translation elongation factor TU	1.75	1.80E-09
YIM1	Protein similar to protease of mitochondrial inner membrane	1.75	3.63E-07
HSP60	Heat shock protein	1.73	3.68E-13
RNR3	Putative ribonucleotide reductase large subunit	1.72	7.33E-11
GAP5	General amino acid permease	1.71	7.67E-08
MDJ1	Putative member of the HSP40 (DnaJ) family of chaperones	1.69	5.21E-07
RPO41	Putative mitochondrial RNA polymerase	1.69	2.75E-06
C5_00570W_A	Putative aminophospholipid translocase (flippase)	1.69	1.62E-07
C6_01620W_A	RING/FYVE/PHD zinc finger protein	1.68	2.37E-13
C3_00840C_A	Ortholog(s) have mitochondrion localization	1.68	2.22E-06
CR_03120W_A	Ortholog of S. cerevisiae Mpm1	1.68	1.90E-15
HAL9	Putative Zn(II)2Cys6 transcription factor	1.66	7.61E-12
TIM23	Protein involved in mitochondrial matrix protein import	1.66	1.04E-14
FET31	Putative multicopper oxidase	1.65	3.85E-06
C1_08520C_A	Ortholog(s) have structural constituent of ribosome activity	1.64	8.63E-12
MIF2	Centromere-associated protein	1.64	1.04E-24
HRR25	Predicted protein serine/threonine kinase	1.63	2.15E-08
CR_10470C_A	Ortholog(s) have role in maturation of SSU-rRNA from tricistronic rRNA transcript	1.63	2.74E-06
MSS116	Putative DEAD-box protein	1.62	7.61E-06
DCK1	Putative guanine nucleotide exchange factor	1.61	4.47E-06
GCN4	bZIP transcription factor	1.61	3.12E-06
C5_02590C_A	Putative mitochondrial membrane protein	1.60	9.23E-06
C2_10090C_A	Ortholog(s) have mitochondrion localization	1.60	1.48E-08
RIM2	Putative mitochondrial carrier protein	1.59	1.58E-07
SSY1	Amino acid sensor	1.58	2.17E-07

ISW2	Ortholog of S. cerevisiae Isw2	1.57	9.68E-09
C6_03440W_A	Ortholog(s) have rRNA binding activity	1.57	1.75E-09
CR_03940W_A	Putative rRNA processing protein	1.56	3.69E-08
HNM1	Putative choline/ethanolamine transporter	1.55	1.41E-07
C2_04790C_A	Ortholog(s) have structural constituent of ribosome activity	1.55	2.93E-09
C2_03490C_A	Ortholog of C. dubliniensis CD36: Cd36_18230	1.54	1.37E-11
CCC1	Manganese transporter	1.52	3.42E-06
C4_03410W_A	Ortholog(s) have structural constituent of ribosome activity	1.52	2.37E-13
GIS2	Translational activator for mRNAs	1.52	9.61E-09
HIP1	Amino acid permeases	1.51	7.94E-14
MGE1	Putative mitochondrial matrix cochaperone	1.51	3.33E-07
CR_09620C_A	Ortholog(s) have phosphatidic acid transporter activity	1.51	5.68E-06
C5_04050W_A	Plasma membrane-localized protein of unknown function	1.50	4.42E-08
MSI3	Essential HSP70 family protein	1.49	2.10E-06
SFP1	C2H2 transcription factor involved in regulation of biofilm formation	1.49	5.13E-06
ZCF29	Zn (II)2Cys6 transcription factor	1.48	4.04E-11
WSC1	Putative cell wall component	1.46	6.09E-10
MIR1	Putative mitochondrial phosphate transporter	1.46	7.19E-07
C3_01850W_A	Ortholog(s) have structural constituent of ribosome activity	1.45	2.84E-07
C1_14190C_A	Protein phosphatase inhibitor	1.45	7.85E-07
MIA40	Predicted component of the mitochondrial intermembrane space import machinery	1.44	1.54E-09
C5_02740W_A	Ortholog(s) have oxidoreductase activity	1.43	1.09E-11
C4_00920C_A	Possible similarity to mutator-like element (MULE) transposase	1.43	7.23E-06
CDC34	Putative ubiquitin-protein ligase	1.43	2.57E-06
RSN1	Protein of unknown function	1.43	1.89E-10
MRPL10	Putative mitochondrial large subunit ribosomal protein	1.43	6.62E-08
C1_01150C_A	Ortholog(s) have tRNA methyltransferase activity	1.43	6.98E-07
C1_13060C_A	Ortholog(s) have role in mitochondrial translation	1.42	6.33E-06
HEM1	Putative 5-aminolevulinate synthase	1.41	9.55E-06
FLO8	Transcription factor	1.41	8.50E-09
CRZ2	C2H2 transcription factor	1.38	4.41E-06
RIM9	Protein required for alkaline pH response	1.38	5.32E-09
C1_06350W_A	Protein of unknown function	1.38	2.87E-08
FGR15	Putative transcription factor with zinc finger DNA-binding motif	1.37	9.45E-11
CR_06230W_A	Predicted cation transmembrane transporter	1.37	2.24E-07
C5_00250C_A	Protein with a predicted role in microtubule-related processes	1.37	3.23E-08
WHI3	Putative RNA binding protein	1.36	2.90E-08
PTC8	Predicted type 2C protein phosphatase	1.34	5.96E-07
MRPL3	Ribosomal protein of the large subunit	1.33	1.79E-06

SCO1	Putative copper transporter	1.33	7.38E-06
C1_04370C_A	Ortholog(s) have structural constituent of ribosome activity	1.33	7.20E-07
C2_02180W_A	Vacuolar membrane zinc transporter	1.33	4.40E-10
C5_01700W_A	Mitochondrial ribosomal protein of the large subunit	1.32	3.86E-06
MSW1	Mitochondrial tryptophanyl-tRNA synthetase	1.32	1.95E-10
C7_03000C_A	Ortholog(s) have structural constituent of ribosome activity	1.32	6.47E-10
MNN23	Alpha-1,2-mannosyltransferase	1.31	5.09E-07
C6_00920W_A	Putative mitochondrial intermembrane space protein	1.31	4.83E-06
DOT6	Protein with a predicted role in telomeric gene silencing and filamentation	1.30	9.45E-06
C1_14090W_A	Ortholog(s) have chaperone binding, unfolded protein binding activity	1.29	2.26E-08
C1_05210C_A	Protein of unknown function	1.29	7.40E-09
SSU81	Predicted adaptor protein	1.28	2.36E-07
FZO1	Mitochondrial biogenesis protein	1.28	4.29E-06
CTA2	Putative transcription factor	1.27	2.93E-07
CR_10420W_A	Protein with a mitochondrial distribution and morphology domain	1.25	7.63E-10
ADA2	Zinc finger and homeodomain transcriptional coactivator	1.24	3.29E-08
CWC22	Predicted spliceosome-associated protein	1.24	1.03E-08
C2_07440C_A	Ortholog(s) have sterol esterase activity	1.22	3.22E-08
BRE1	Putative transcription factor with C3HC4 zinc finger DNA-binding motif	1.21	5.25E-06
C6_03730C_A	Ortholog(s) have structural constituent of ribosome activity	1.19	7.21E-07
C1_14330W_A	Ortholog(s) have tRNA-5-taurinomethyluridine 2-sulfurtransferase activity	1.19	1.57E-06
LCB4	Putative sphingosine kinase	1.18	2.64E-06
C7_01910C_A	Similar to bacterial DnaJ	1.17	4.57E-09
CR_08470W_A	Protein involved in N-glycosylation	1.16	2.84E-07
C2_05380W_A	Protein of unknown function	1.16	3.59E-06
CR_04580W_A	Ortholog(s) have structural constituent of ribosome activity	1.16	9.24E-06
OPY2	Predicted transmembrane protein	1.15	2.85E-06
REG1	Putative protein phosphatase regulatory subunit	1.14	1.84E-06
STP2	Amino-acid-regulated transcription factor	1.14	3.24E-06
DFG5	N-linked mannoprotein	1.13	1.79E-07
C1_13880C_A	C2H2 transcription factor	1.12	8.35E-08
KCS1	Putative bZIP transcription factor	1.12	1.31E-07
C4_00910C_A	Protein with similarity to mutator-like element (MULE) transposase	1.12	1.26E-09
C2_03560C_A	Ortholog(s) have structural constituent of ribosome activity	1.10	3.74E-06
C3_03250W_A	Putative haloacid dehalogenase	1.09	6.73E-06
YOR1	ABC-type plasma membrane transporter	1.09	4.21E-08
ALS6	ALS family protein	1.08	2.56E-08
C1_11850W_A	Protein of unknown function	1.08	4.77E-06

CHO2	Putative phosphatidyl-ethanolamine N-methyltransferase	1.06	9.19E-07
CR_01370C_A	Ortholog(s) have structural constituent of ribosome activity	1.06	4.40E-06
CR_03110W_A	Ortholog(s) have role in mitochondrial translation	1.05	5.42E-07
SMC4	Ortholog(s) have ATPase activity	1.05	6.73E-06
RPN4	C2H2 transcription factor	1.03	8.03E-06
C4_04240C_A	Ortholog of S. cerevisiae Skg6	1.02	5.61E-06
AFG3	a subunit of the mitochondrial inner membrane m-AAA protease	1.02	4.94E-13
ARF1	ADP-ribosylation factor	1.01	1.42E-07
IFM1	Putative mitochondrial translation initiation factor	0.99	7.11E-06
C7_03840W_A	CCR4-NOT complex component	0.98	1.80E-06
C2_00730C_A	Ortholog of C. dubliniensis CD36 : Cd36_15670	0.98	7.61E-17
NBP35	Similar to nucleotide-binding proteins	0.97	3.15E-06
C5_00350C_A	Putative DnaJ-like chaperone	0.96	4.07E-06
C4_03780C_A	Putative mitochondrial membrane protein	0.95	1.58E-07
C6_00390W_A	Ortholog(s) have serine-tRNA ligase activity	0.95	6.24E-08
C1_02750C_A	Ortholog(s) have DNA translocase activity	0.94	5.07E-06
C2_07060W_A	Ortholog(s) have RNA binding activity	0.92	5.68E-06
SWI6	Putative component of the MBF and SBF transcription complexes	0.84	1.97E-09
C6_03460W_A	Nucleoporin component of central core of the nuclear pore complex	0.82	1.70E-06
PBP2	Putative RNA binding protein	0.79	1.57E-06
MBP1	Putative component of the MBF transcription complex	0.77	1.16E-06
WAR1	Zn(II)2Cys6 transcription factor	0.77	4.25E-07
PDR6	Putative pleiotropic drug resistance regulatory protein 6	0.73	2.56E-08
C5_03940C_A	Putative multidrug resistance protein; upregulated by Efg1p	-0.63	2.93E-06
C1_02720W_A	Putative transcription factor	-0.67	1.16E-06
C3_06260C_A	Ortholog of *C. dubliniensis* CD36 : Cd36_86220	-0.68	9.05E-06
VPS8	Putative vacuolar sorting protein	-0.70	9.83E-08
ZCF9	Putative Zn(II)2Cys6 transcription factor	-0.70	8.35E-09
C3_05010C_A	Putative DNA repair helicase	-0.78	1.13E-06
C7_01650W_A	RNA polymerase III transcription initiation factor complex (TFIIIC) subunit	-0.79	9.93E-06
PSD1	Phosphatidylserine decarboxylase	-0.81	8.54E-06
C2_01490C_A	Putative RNA polymerase transcription factor TFIIH core component	-0.82	9.00E-06
APL5	Ortholog of *S. cerevisiae* and *S. pombe* Apl5	-0.88	1.19E-06
C4_01790W_A	Adapter protein for pexophagy and the cytoplasm-to-vacuole targeting (Cvt) pathway	-0.88	2.60E-06
ORC3	A component of the origin recognition complex	-0.89	7.15E-07
CWH43	Putative sensor/transporter protein	-0.90	1.14E-06

CR_08550W_A	Ortholog(s) have fructose-2,6-bisphosphate 2-phosphatase activity	-0.91	5.29E-06
CR_01130W_A	Ortholog(s) have role in positive regulation of TORC1 signaling	-0.93	4.86E-08
GPA2	G-protein alpha subunit	-0.93	6.18E-06
PKH2	Putative serine/threonine protein kinase	-0.93	3.45E-07
POM152	Putative nuclear pore membrane glycoprotein	-0.94	2.53E-07
C1_12740W_A	Predicted cysteine proteinase domain	-0.95	4.93E-07
MKC1	MAP kinase	-0.96	6.33E-06
RTA2	Flippase	-0.96	2.22E-07
CR_06470W_A	Ortholog(s) have ubiquitin protein ligase activity	-0.98	8.06E-06
C3_02690C_A	Ortholog(s) have dolichol kinase activity	-0.99	3.45E-06
C1_00920W_A	Ortholog(s) have cell division site	-0.99	1.10E-06
LYS12	Homoisocitrate dehydrogenase	-1.01	4.08E-06
C3_07230W_A	Aminophospholipid translocase (flippase)	-1.01	3.72E-11
C2_02020W_A	Ortholog(s) have Lys48-specific deubiquitinase activity	-1.01	9.92E-06
C5_01350W_A	Ortholog(s) have role in ER-dependent peroxisome organization	-1.01	5.36E-07
ERV46	Putative ER-derived vesicle protein	-1.03	5.57E-07
C5_00330C_A	Ortholog(s) have role in fermentation	-1.03	1.25E-06
C3_03430C_A	Ortholog(s) have role in ATP export	-1.03	3.64E-07
HEM2	Putative porphobilinogen synthase	-1.04	7.87E-07
C1_04780C_A	Ortholog(s) have role in negative regulation of TORC1 signaling	-1.04	6.72E-13
C4_00380W_A	Ortholog(s) have D-arabinose 1-dehydrogenase [NAD(P)+] activity	-1.04	9.05E-08
VPS1	Dynamin-family GTPase-related protein	-1.04	1.06E-06
C2_05850C_A	Ortholog(s) have role in CVT pathway	-1.05	1.52E-07
SSN8	A component of RNA polymerase II holoenzyme	-1.06	9.32E-08
CR_05890C_A	Ortholog(s) have acetyltransferase activator activity	-1.06	5.67E-08
NUP82	Linker nucleoporin of the nuclear pore complex	-1.07	2.16E-06
SHE3	mRNA-binding protein	-1.07	9.76E-08
C4_03130W_A	Putative GPI transamidase component	-1.08	2.57E-06
C2_07010W_A	Predicted ER protein	-1.08	3.80E-08
C1_03330C_A	Ortholog(s) have phosphatidylinositol-3,5-bisphosphate binding activity	-1.09	1.19E-12
C4_05800C_A	Protein of unknown function	-1.09	4.92E-06
CHS7	Protein required for wild-type chitin synthase III activity	-1.10	1.74E-07
C5_01090C_A	Planktonic growth-induced gene	-1.11	9.93E-06
SOL3	Putative 6-phosphogluconolactonase	-1.11	6.06E-06
C5_04320C_A	RNI-like superfamily domain-containing protein	-1.11	2.02E-08
CR_04080C_A	Endoplasmic reticulum (ER) protein-translocation complex subunit	-1.11	4.82E-09
PCT1	Putative choline-phosphate cytidylyl transferase	-1.11	5.81E-06

CAS4	RAM cell wall integrity signaling network protein	-1.11	3.47E-09
C4_01430C_A	Protein of unknown function	-1.12	5.97E-06
C1_12400C_A	Ortholog of Candida albicans WO-1 : CAWG_00201	-1.12	4.40E-06
GSL2	Protein similar to beta-1,3-glucan synthase	-1.12	6.15E-06
CCH1	Voltage-gated Ca2+ channel of the high affinity calcium uptake system	-1.13	2.72E-11
USO1	Ortholog(s) have role in ER to Golgi vesicle-mediated transport	-1.13	4.70E-07
VPS36	ESCRT II protein sorting complex subunit	-1.13	2.56E-08
C1_10140C_A	Protein of unknown function	-1.14	5.41E-06
LSC1	Putative succinate-CoA ligase subunit	-1.15	4.83E-06
C4_06020C_A	Ortholog(s) have DNA-directed DNA polymerase activity	-1.16	1.04E-10
C3_06800C_A	Ortholog(s) have NEDD8 transferase activity	-1.16	2.26E-06
C1_12170C_A	Ortholog(s) have cytoplasm localization	-1.17	2.67E-06
MYO2	Class V myosin	-1.17	3.75E-06
C7_00140C_A	Ortholog(s) have cytosol, nucleus localization	-1.17	2.59E-10
CDC10	Septin	-1.18	7.23E-06
AGE1	Putative GTPase activator	-1.18	1.37E-07
C5_02050W_A	Ortholog(s) have role in Golgi to endosome transport	-1.18	3.08E-10
CR_09340W_A	Ortholog(s) have ubiquitin conjugating enzyme activity	-1.19	5.25E-08
C1_02440C_A	Ortholog(s) have thiol-dependent ubiquitin-specific protease activity	-1.19	3.06E-06
INP51	Putative phosphatidylinositol-4,5-bisphosphate phosphatase	-1.19	1.13E-06
DBR1	Debranchase	-1.19	6.22E-08
AYR1	Putative oxidoreductase	-1.19	3.37E-06
GIG1	Protein induced by N-acetylglucosamine (GlcNAc	-1.20	3.36E-06
SEN1	Putative helicase	-1.20	4.61E-08
IML2	Protein of unknown function	-1.20	1.22E-07
TUB1	Alpha-tubulin	-1.21	2.30E-07
TIP120	protein involved in regulation of SCF complexes	-1.21	6.36E-06
C5_01940W_A	Ortholog(s) have 2,5-diamino-6-ribitylamino-4(3H)-pyrimidinone 5'-phosphate deaminase activity	-1.21	4.23E-09
PTP3	Putative protein tyrosine phosphatase	-1.21	1.17E-07
CLA4	Ste20p family Ser/Thr kinase	-1.21	3.92E-06
CR_08430W_A	A protein involved in regulated synthesis of PtdIns(3,5)P(2)	-1.21	5.04E-12
C1_01400C_A	Ortholog(s) have sphingosine-1-phosphate phosphatase activity	-1.23	1.72E-06
TEL1	Ortholog(s) have protein serine/threonine kinase activity	-1.23	3.29E-16
C2_07650C_A	Ortholog(s) have extracellular region localization	-1.24	9.75E-06
C3_06750W_A	Ortholog(s) have P-P-bond-hydrolysis-driven protein transmembrane transporter activity	-1.24	1.60E-06
NPT1	Putative nicotinate phosphoribosyltransferase	-1.25	3.21E-11
BMT3	Beta-mannosyltransferase	-1.26	1.29E-06

CR_01080W_A	Putative ortholog of *S. cerevisiae* Laa1p	-1.26	3.63E-07
C1_08180C_A	Putative oxysterol-binding protein	-1.26	5.83E-06
RBK1	Ortholog(s) have cytosol, nucleus localization	-1.27	3.72E-06
C2_05720C_A	Putative oxysterol binding protein family	-1.27	3.05E-13
SEC7	Putative guanine nucleotide exchange factor	-1.27	1.97E-11
C4_06130W_A	Ortholog(s) have tubulin binding activity	-1.28	3.86E-09
C3_02080W_A	Has domain(s) with predicted zinc ion binding activity	-1.28	1.09E-11
C1_02770W_A	Protein of unknown function	-1.29	7.82E-09
KTR4	Mannosyltransferase	-1.30	1.16E-07
ARO80	Zn(II)2Cys6 transcription factor	-1.30	3.47E-14
COQ6	Ortholog(s) have oxidoreductase activity	-1.31	1.17E-12
C6_00890W_A	Ortholog(s) have cytoplasm localization	-1.31	5.54E-09
C6_01960W_A	Ortholog of C. dubliniensis CD36 : Cd36_62090	-1.32	6.36E-06
CYK3	Essential protein involved in cytokinesis	-1.32	1.76E-07
SEC13	Putative protein transport factor	-1.32	1.34E-07
MPT5	Putative RNA-binding protein	-1.34	1.22E-11
ACF2	Putative endo-1,3-beta-glucanase	-1.34	2.86E-07
C3_07770C_A	Ortholog(s) have microtubule binding, protein homodimerization activity	-1.34	1.29E-10
BFR1	Protein involved in the maintenance of normal ploidy	-1.34	4.07E-07
LIG4	DNA ligase	-1.35	5.14E-10
C2_09480W_A	Ortholog(s) have DNA-directed DNA polymerase activity	-1.35	2.03E-08
RHO2	Ortholog(s) have GTPase activity	-1.36	1.62E-07
MRP8	Mitochondrial ribosomal protein	-1.37	2.90E-06
STT3	Putative oligosaccharyltransferase complex component	-1.37	6.34E-07
BZZ1	SH3 domain protein involved in the regulation of actin polymerization	-1.39	2.23E-07
RNR1	Ribonucleotide reductase large subunit	-1.39	1.73E-06
UBA4	Putative ubiquitin activating protein	-1.39	1.33E-11
C4_01280C_A	Ortholog(s) have RNA binding activity	-1.39	3.79E-10
C1_14170W_A	Ortholog(s) have ubiquitin-protein transferase activity	-1.40	1.73E-10
CR_02880W_A	Protein of unknown function	-1.40	4.55E-06
SCT2	Putative glycerol-3-phosphate acyltransferase;	-1.40	2.26E-08
MYO1	Component of actomyosin ring at neck of newly emerged bud	-1.40	1.59E-14
CHS5	Putative chitin biosynthesis protein	-1.42	5.94E-08
C2_00230W_A	Ortholog(s) have arginyltransferase activity	-1.42	1.84E-10
CR_00130C_A	Protein with a pyridoxal phosphate-dependent transferase domain	-1.44	7.24E-06
SMP2	Putative Mg2+-dependent phosphatidate phosphatase	-1.45	4.40E-07
C1_07260C_A	Ortholog(s) have DNA replication origin binding, single-stranded DNA binding activity	-1.46	1.16E-21
PMM1	Phosphomannomutase	-1.46	7.69E-08
C1_01930W_A	Protein of unknown function	-1.47	2.13E-06

SET3	NAD-dependent histone deacetylase	-1.47	2.72E-11
BET2	Putative Type II geranylgeranyltransferase beta subunit	-1.48	5.13E-12
CR_02300C_A	Protein with a Vps9 vacuolar protein sorting protein domain	-1.48	2.37E-13
PDS5	Putative protein with a predicted role in establishment and maintenance of sister chromatid condensation and cohesion	-1.49	1.04E-07
NCP1	NADPH-cytochrome P450 reductase	-1.49	1.30E-12
C3_06710W_A	Protein of unknown function	-1.50	1.83E-18
FAS2	Alpha subunit of fatty-acid synthase	-1.50	4.22E-06
C3_04460W_A	Ortholog(s) have role in positive regulation of TORC1 signaling and Seh1-associated complex	-1.50	2.82E-08
USO6	Putative vesicular transport protein	-1.50	1.83E-13
CDC3	Septin	-1.51	8.19E-12
BTS1	Putative geranylgeranyl diphosphate synthase	-1.51	8.03E-12
MAE1	Malic enzyme	-1.51	3.13E-07
CYB5	Cytochrome b(5)	-1.53	4.28E-06
CR_01280C_A	Ortholog(s) have role in intracellular sterol transport	-1.54	5.24E-08
C1_03100W_A	Putative lipid-binding protein	-1.55	9.48E-09
GTT11	Glutathione S-transferase	-1.55	7.07E-10
SVF1	Putative survival factor	-1.55	1.49E-11
C6_02680W_A	Ortholog(s) have SNAP receptor activity	-1.56	3.17E-08
C5_04350C_A	Ortholog(s) have cell division site, cell tip, cytosol, nucleus localization	-1.56	2.51E-12
C1_06700W_A	SH3-domain protein	-1.56	2.23E-07
ECM33	GPI-anchored cell wall protein	-1.57	1.85E-08
DYN1	Dynein heavy chain	-1.57	4.37E-19
PMT2	Protein mannosyltransferase (PMT)	-1.57	1.26E-08
C5_04330W_A	Putative adhesin-like protein	-1.57	2.36E-06
C4_06990W_A	Possible mannosyltransferase	-1.59	4.86E-14
PGA53	GPI-anchored cell surface protein of unknown function	-1.59	1.41E-07
YKE2	Possible heterohexameric Gim/prefoldin protein complex subunit	-1.60	1.48E-06
PRN4	Protein with similarity to pirins	-1.60	3.43E-06
C4_00710W_A	Has domain(s) with predicted ATP binding activity	-1.60	1.91E-08
C6_01950C_A	Protein of unknown function	-1.61	1.44E-07
C1_07050C_A	Ortholog(s) have ATP-dependent 5'-3' DNA helicase activity	-1.61	1.09E-11
C1_09220W_A	Predicted MFS family membrane transporter	-1.63	1.03E-09
RAD50	Putative DNA double-strand break repair factor	-1.64	1.94E-11
CDC11	Septin	-1.65	4.36E-06
C3_03370C_A	Protein of unknown function	-1.67	7.02E-10
C6_02490C_A	Protein of unknown function	-1.68	5.17E-10
PGI1	Glucose-6-phosphate isomerase	-1.68	4.50E-06
CR_06270W_A	Predicted protein of unknown function	-1.69	1.81E-06

ENO1	Enolase	-1.69	7.99E-06
C6_02650C_A	Predicted membrane transporter	-1.69	1.97E-10
C2_08620W_A	Protein of unknown function	-1.71	1.09E-09
CR_00290W_A	Ortholog of S. cerevisiae Rts3; a component of the protein phosphatase type 2A complex	-1.71	1.53E-12
MON2	Peripheral membrane protein	-1.72	1.03E-10
CLN3	G1 cyclin	-1.73	4.62E-07
SEC61	ER protein-translocation complex subunit	-1.73	7.05E-06
C1_11120C_A	Protein similar to S. cerevisiae Gvp36p	-1.74	7.21E-20
REV3	Ortholog(s) have DNA-directed DNA polymerase activity	-1.74	7.15E-11
CDC12	Septin	-1.75	2.88E-11
FBA1	Fructose-bisphosphate aldolase	-1.76	1.55E-09
KRE5	UDP-glucose:glycoprotein glucosyltransferase	-1.77	8.50E-08
EXG2	GPI-anchored cell wall protein	-1.78	3.25E-09
C7_02850W_A	Ortholog of Candida albicans WO-1 : CAWG_05627	-1.78	1.26E-12
SEC23	Putative GTPase-activating protein	-1.78	4.88E-08
C1_02780W_A	Predicted dienelactone hydrolase domain	-1.79	1.24E-08
FCY2	Purine-cytosine permease of pyrimidine salvage	-1.82	2.03E-07
C4_05250W_A	Putative ubiquitin-protein ligase	-1.83	2.58E-09
C5_03490C_A	Stationary phase enriched protein	-1.83	8.07E-17
AGO1	Putative Argonaute protein	-1.83	2.64E-06
C1_11250W_A	Microtubule-binding protein	-1.84	6.83E-15
C5_02520W_A	Protein of unknown function	-1.85	1.94E-11
MTS1	Sphingolipid C9-methyltransferase	-1.85	7.43E-12
C1_09130W_A	Protein of unknown function	-1.86	3.11E-09
PMI1	Phosphomannose isomerase	-1.87	2.84E-07
ACB1	Protein similar to a region of acyl-coenzyme-A-binding protein	-1.88	2.34E-06
PMT4	Protein mannosyltransferase	-1.89	2.35E-07
NAT4	Putative histone acetyltransferase	-1.90	1.69E-10
STE13	Putative pheromone-processing dipeptidyl aminopeptidase	-1.92	8.23E-16
CR_09330C_A	Protein with a Staphylococcal nuclease domain	-1.93	1.40E-11
C5_02850W_A	H/ACA box small nucleolar RNA (snoRNA)	-1.94	5.62E-08
C2_02220C_A	Protein of unknown function	-1.94	3.76E-07
TPM2	Putative tropomyosin isoform 2	-1.95	2.44E-09
PSD2	Ortholog(s) have phosphatidylserine decarboxylase activity	-1.95	3.88E-22
TPK2	cAMP-dependent protein kinase catalytic subunit	-1.96	8.11E-11
DPM1	Dolichol-phosphate mannose synthase catalytic subunit	-1.98	2.55E-11
SSO2	Plasma membrane t-SNARE	-1.98	3.53E-30
C1_04470C_A	Protein of unknown function	-2.00	1.35E-10
INN1	Protein with similarity to *S. cerevisiae* Inn1p	-2.02	1.71E-29
PST3	Flavodoxin-like protein	-2.02	1.62E-06
CDC19	Pyruvate kinase	-2.04	1.43E-09

ERG11	Lanosterol 14-alpha-demethylase	-2.04	1.72E-07
DAP1	Similar to mammalian membrane-associated progesterone receptors involved in DNA damage response	-2.08	7.04E-21
C2_07220W_A	Putative 20S proteasome assembly protein	-2.10	3.22E-11
GST3	Glutathione S-transferase	-2.11	7.62E-06
YVC1	Putative vacuolar cation channel shock	-2.13	1.40E-14
CEK1	ERK-family protein kinase	-2.14	9.93E-15
C7_01390W_A	Protein of unknown function	-2.16	2.02E-07
CRL1	Predicted GTPase of RHO family	-2.16	2.30E-24
ERG2	C-8 sterol isomerase	-2.18	7.43E-12
CR_09040W_A	Protein of unknown function	-2.18	5.37E-08
GND1	6-phosphogluconate dehydrogenase	-2.19	2.01E-06
C3_04420W_A	Ortholog of *S. cerevisiae* Hul4	-2.19	9.81E-12
SLD1	Sphingolipid delta-8 desaturase	-2.20	1.06E-17
MSC7	*S. cerevisiae* ortholog Mcs7	-2.23	8.80E-52
NIP100	p150 subunit of dynactin	-2.25	6.27E-12
C3_04740C_A	Ortholog(s) have DNA topoisomerase type I activity	-2.26	1.94E-11
C2_09810C_A	Protein with predicted peptidase domains	-2.27	1.69E-07
CR_04870C_A	Trimethylaminobutyraldehyde dehydrogenase	-2.27	3.11E-09
MNN12	Predicted alpha-1,3-mannosyltransferase	-2.27	1.01E-09
SOD4	Cu-containing superoxide dismutase	-2.30	2.10E-08
MLC1	Microtubule-dependent localized protein	-2.30	5.78E-18
GAL4	Zn(II)2Cys6 transcription factor	-2.31	3.58E-13
GFA1	Glucosamine-6-phosphate synthase	-2.31	1.18E-13
LAP41	Putative aminopeptidase yscI precursor	-2.31	6.47E-11
C5_01070C_A	Protein of unknown function	-2.34	6.20E-08
C1_11790W_A	Ortholog(s) have thymidylate kinase activity	-2.36	7.23E-16
ZWF1	Glucose-6-phosphate dehydrogenase	-2.36	2.18E-08
ERG5	Putative C-22 sterol desaturase	-2.37	3.22E-11
C4_00860C_A	Protein of unknown function	-2.38	4.82E-11
C1_09980C_A	Ortholog(s) have acylglycerol lipase activity	-2.41	1.63E-09
ERG27	3-Keto sterol reductase of ergosterol biosynthesis	-2.42	5.26E-14
PGA54	GPI-anchored protein	-2.43	1.37E-10
CHS8	Chitin synthase	-2.43	4.32E-19
CR_04770C_A	Ortholog of C. dubliniensis CD36 : Cd36_30140	-2.43	3.73E-18
CHS2	Chitin synthase	-2.46	2.51E-41
C6_03320W_A	Stationary phase enriched protein	-2.47	1.43E-06
C1_04580C_A	Ortholog of Rmd6 involved in *S. cerevisiae* sporulation	-2.48	1.05E-19
CR_09070C_A	Protein of unknown function	-2.49	5.32E-17
ERG10	Acetyl-CoA acetyltransferase	-2.49	1.94E-11
C1_11730W_A	Protein with SEL-1 like protein domain	-2.50	3.63E-25
C1_11720W_A	Ortholog of Candida albicans WO-1 : CAWG_00265	-2.51	7.55E-10
UPC2	Zn2-Cys6 transcript factor	-2.55	3.81E-41

C3_06670C_A	Protein of unknown function	-2.55	2.39E-06
C4_01860C_A	Possible Golgi membrane protein	-2.56	1.24E-11
CR_04120C_A	Ortholog(s) have DNA-dependent ATPase activity	-2.61	1.81E-17
C1_11260C_A	Putative adhesin-like protein	-2.66	2.18E-14
ERG1	Squalene epoxidase	-2.68	8.42E-31
C5_04260W_A	Has domain(s) with predicted RNA binding	-2.68	2.26E-08
ERG26	C-3 sterol dehydrogenase	-2.68	8.72E-20
IHD1	GPI-anchored protein	-2.75	1.98E-06
C6_02200C_A	Protein of unknown function	-2.75	1.83E-07
CHO1	Putative phosphatidylserine synthase	-2.80	1.92E-15
PGA6	GPI-anchored cell wall adhesin-like protein	-2.81	3.29E-09
C7_02260W_A	Ortholog of C. parapsilosis CDC317	-2.85	1.13E-06
C1_13490C_A	Ortholog(s) have role in cellular calcium ion homeostasis	-2.90	1.74E-35
LDG3	Putative LDG family protein	-2.90	3.08E-06
ECM331	GPI-anchored protein	-2.93	2.62E-11
C2_02910W_A	Protein of unknown function	-3.03	6.12E-12
BMT7	Beta-mannosyltransferase	-3.04	3.11E-13
C6_03260W_A	Putative ribonuclease H1	-3.06	1.15E-20
RBT4	Pry family protein	-3.10	1.73E-11
ERG4	Protein similar to sterol C-24 reductase	-3.10	1.64E-11
DAG7	Secretory protein	-3.19	1.06E-09
C6_02100W_A	Secreted potein	-3.21	8.98E-09
ERG24	C-14 sterol reductase	-3.25	3.37E-29
IPK2	Putative inositol polyphosphate multikinase	-3.46	2.13E-39
C7_03310W_A	Protein of unknown function	-3.59	2.59E-10
RBR1	Glycosylphosphatidylinositol (GPI)-anchored cell wall protein	-3.60	2.87E-12
ERG6	Delta(24)-sterol C-methyltransferase	-3.70	1.37E-25
DDR48	Immunogenic stress-associated protein	-3.78	1.95E-14
C1_04590W_A	Protein of unknown function	-3.79	2.46E-29
HWP1	Hyphal cell wall protein	-4.10	7.81E-10
C3_06660C_A	Ortholog of C. dubliniensis CD36 : Cd36_86630	-4.16	3.09E-10
PGA45	Putative GPI-anchored cell wall protein	-4.19	2.22E-13
C2_10160W_A	Secreted protein	-4.45	2.94E-28
PGA23	Putative GPI-anchored protein of unknown function	-4.52	3.16E-23
CRH11	GPI-anchored cell wall transglycosylase	-4.67	2.00E-30
MRV8	Protein of unknown function	-5.46	4.10E-22
PGA13	GPI-anchored cell wall protein involved in cell wall synthesis	-5.67	2.51E-41
C3_01540W_A	Plasma-membrane-localized protein	-6.68	5.72E-43

Supplementary Table S6: Gene expression data; Significantly upregulated/downregulated genes in *C. albicans* treated with C12AHL compared to fluconazole only treated samples. The genes that are known to be associated with antimicrobial sensitivity/resistance are shaded in light blue (adjusted *p-value* < $1e^{-5}$).

Name	annotation	log2 Fold Change	Adjusted P value
JEN2	Dicarboxylic acid transporter	5.30	9.71E-47
ATX1	Putative cytosolic copper metallochaperone	4.96	4.99E-48
RME1	Zinc finger protein	3.94	2.76E-27
CRP1	Copper transporter	3.84	6.55E-26
HSP30	Putative heat shock protein	3.77	4.01E-14
IFE2	Putative alcohol dehydrogenase	3.61	7.29E-14
OPT3	Oligopeptide transporter	3.59	2.19E-30
ADH3	Putative NAD-dependent (R,R)-butanediol dehydrogenase	3.51	3.26E-10
C1_11320C_A	Protein of unknown function	3.50	2.29E-18
HSP104	Heat-shock protein	3.41	5.31E-16
C5_04980W_A	Putative adhesin-like protein	3.40	8.25E-17
PHO84	High-affinity phosphate transporter	3.38	3.55E-11
FCY21	High affinity, high capacity, hypoxanthine-adenine-guanine-cytosine/H+ symporter	3.33	5.32E-17
HSP78	Heat-shock protein	3.32	4.14E-20
HNM3	Putative transporter	3.29	5.02E-12
TPO3	Putative polyamine transporter	3.28	5.64E-19
CDR4	Putative ABC transporter superfamily	3.19	3.03E-40
UGT51C1	UDP-glucose: sterol glucosyltransferase	3.03	1.65E-38
C7_03560W_A	Protein of unknown function	3.02	1.07E-28
ZRT2	Zinc transporter	3.02	3.64E-15
C7_00350C_A	Protein of unknown function	3.02	2.71E-10
C4_07140W_A	Predicted mitochondrial intermembrane space protein	2.94	1.58E-19
CTN1	Carnitine acetyl transferase	2.90	6.21E-11
SSA2	HSP70 family chaperone	2.86	2.44E-09
CR_08270W_A	Protein of unknown function	2.84	1.35E-06
GCV2	Glycine decarboxylase P subunit	2.84	2.36E-10
ALP1	Cystine transporter	2.80	6.01E-12
HSP21	Small heat shock protein	2.72	2.65E-08
FMP45	Predicted membrane protein induced during mating	2.68	5.50E-08
DLD1	Putative D-lactate dehydrogenase	2.60	3.81E-12
CAN1	Basic amino acid permease	2.59	3.90E-08
C6_00810C_A	Protein of unknown function	2.59	7.90E-09
SIT1	Transporter of ferrichrome siderophores	2.57	4.54E-08
C1_07160C_A	Protein conserved among the CTG-clade	2.51	6.56E-08
C1_08350C_A	Ortholog of *C. dubliniensis* CD36 : Cd36_02630	2.44	8.75E-15
RBE1	Pry family cell wall protein	2.37	1.16E-06
C2_00760C_A	Protein of unknown function	2.29	1.47E-11
AQY1	Aquaporin water channel	2.27	8.18E-09
GIT3	Glycerophosphocholine permease	2.25	9.75E-12
CR_08880C_A	Protein of unknown function	2.24	1.67E-12
C7_01170C_A	Putative oxidoreductase	2.23	2.39E-06
C2_03110W_A	Protein of unknown function	2.23	7.53E-10
C7_04090C_A	Predicted mitochondrial cardiolipin-specific phospholipase	2.22	3.03E-10
ARG1	Argininosuccinate synthase	2.18	1.91E-07
CTA4	Zn(II)2Cys6 transcription factor	2.16	2.65E-10

C6_03180C_A	Ortholog(s) have protein binding, bridging, ubiquitin protein ligase binding activity	2.15	1.45E-08
RPS27A	Ribosomal protein S27	2.13	8.62E-09
GNP1	Similar to asparagine and glutamine permease	2.11	4.72E-14
MUP1	Putative high affinity methionine permease	2.09	1.06E-06
SIS1	Putative Type II HSP40 co-chaperone	2.09	1.49E-07
CDR11	Putative transporter of PDR subfamily of ABC family	2.09	1.23E-16
C6_00290W_A	Protein of unknown function	2.08	1.44E-13
GOR1	Ortholog(s) have glyoxylate reductase activity	2.06	2.31E-08
C7_00770W_A	Protein of unknown function	2.05	3.57E-08
CAN3	Predicted amino acid transmembrane transporter	2.05	1.34E-12
SNQ2	Protein similar to S. cerevisiae Snq2p transporter	2.04	2.27E-12
PHO87	Putative phosphate permease	2.02	1.24E-08
SSC1	Heat shock protein	2.01	8.61E-17
CPH2	Myc-bHLH transcription factor	1.96	1.30E-15
C2_08100W_A	Mitochondrial apoptosis-inducing factor	1.96	1.92E-06
ALR1	Putative transporter of divalent cations	1.95	9.50E-06
CR_08420W_A	Putative DnaJ-like heat shock/chaperone	1.93	6.87E-06
C1_13820C_A	Protein of unknown function	1.92	8.60E-08
C7_00880C_A	Protein of unknown function	1.91	2.35E-09
COX15	Cytochrome oxidase assembly protein	1.91	8.42E-09
GIT2	Putative glycerophosphoinositol permease	1.90	2.56E-08
C3_01260C_A	Putative adhesin-like protein	1.89	2.31E-08
C4_03370C_A	Ortholog of *C. parapsilosis* CDC317 : CPAR2_403360	1.88	7.31E-07
C1_01130W_A	Putative ubiquitin ligase complex component	1.88	5.64E-19
GRF10	Putative homeodomain transcription factor	1.86	8.93E-08
PUT1	Putative proline oxidase	1.85	4.85E-07
CAL0000191350	Ortholog(s) have ATP binding, DNA replication origin binding activity	1.84	2.14E-12
NDT80	Ortholog of Ndt80	1.83	2.15E-09
SUL2	Putative sulfate transporter	1.83	1.20E-08
C7_03590C_A	Putative member of the multi-drug and toxin extrusion (MATE) family	1.82	4.86E-06
ITR1	MFS inositol transporter	1.80	3.55E-09
DED1	Predicted ATP-dependent RNA helicase	1.79	3.07E-07
PTR22	Oligopeptide transporter involved in uptake of di-/tripeptides	1.78	1.88E-06
C6_03200W_A	Ortholog of *C. dubliniensis* CD36: Cd36_63860	1.78	2.34E-12
ARD	D-arabitol dehydrogenase	1.77	3.88E-16
C4_05900C_A	Protein of unknown function	1.77	6.46E-12
STI1	Protein that interacts with Cdc37 and Crk1 in two-hybrid	1.77	1.19E-06
PLB3	GPI-anchored cell surface phospholipase B	1.77	6.28E-07
ALS7	ALS family protein	1.75	6.00E-22
CR_07170W_A	Possible dehydrogenase	1.73	1.17E-08
HSP90	Essential chaperone	1.72	6.74E-07
C5_02590C_A	Putative mitochondrial membrane protein	1.71	2.26E-06
MET2	Homoserine acetyltransferase	1.70	1.71E-06
HSP60	Heat shock protein	1.70	1.49E-12
YIM1	Protein similar to protease of mitochondrial inner membrane	1.69	9.97E-07
KGD1	Putative 2-oxoglutarate dehydrogenase	1.68	1.23E-06
ROA1	Putative PDR-subfamily ABC transporter	1.62	2.65E-07

PDE2	High affinity cyclic nucleotide phosphodiesterase	1.62	1.52E-08
HAL9	Putative Zn(II)2Cys6 transcription factor	1.61	3.46E-11
C3_00840C_A	Ortholog(s) have mitochondrion localization	1.60	8.66E-06
MDJ1	Putative member of the HSP40 (DnaJ) family of chaperones	1.59	3.15E-06
DCK1	Putative guanine nucleotide exchange factor	1.57	9.29E-06
GAP5	General amino acid permease	1.57	1.27E-06
CR_03120W_A	Ortholog of S. cerevisiae Mpm1	1.57	1.62E-13
C1_05520W_A	Protein of unknown function	1.56	2.73E-06
RNR3	Putative ribonucleotide reductase large subunit	1.56	5.67E-09
MNN22	Alpha-1,2-mannosyltransferase	1.56	5.89E-11
C1_08520C_A	Ortholog(s) have structural constituent of ribosome activity	1.55	1.38E-10
RIM101	Transcription factor	1.54	3.88E-08
C2_10090C_A	Ortholog(s) have mitochondrion localization	1.54	5.89E-08
RIM2	Putative mitochondrial carrier protein	1.54	4.81E-07
ROD1	Protein similar to *S. cerevisiae* Rod1	1.52	9.08E-06
C6_01620W_A	RING/FYVE/PHD zinc finger protein	1.52	5.36E-11
C6_03440W_A	Ortholog(s) have rRNA binding activity	1.52	7.14E-09
SSY1	Amino acid sensor	1.50	1.12E-06
TUF1	Translation elongation factor TU	1.49	5.38E-07
ZCF29	Zn(II)2Cys6 transcription factor	1.49	3.87E-11
MIF2	Centromere-associated protein	1.48	3.07E-20
CR_03940W_A	Putative rRNA processing protein	1.47	3.18E-07
C5_00570W_A	Putative aminophospholipid translocase (flippase)	1.46	8.94E-06
TIM23	Protein involved in mitochondrial matrix protein import	1.44	3.55E-11
HRR25	Predicted protein serine/threonine kinase	1.43	1.41E-06
C2_04790C_A	Ortholog(s) have structural constituent of ribosome activity	1.40	1.16E-07
C5_02740W_A	Ortholog(s) have oxidoreductase activity	1.40	3.97E-11
C2_02180W_A	Ortholog of *S. cerevisiae* Zrt3	1.39	5.74E-11
MGE1	Putative mitochondrial matrix cochaperone	1.38	4.07E-06
RSN1	Protein of unknown function	1.38	1.10E-09
C1_14190C_A	Protein phosphatase inhibitor	1.37	3.89E-06
C1_01150C_A	Ortholog(s) have tRNA methyltransferase activity	1.37	2.44E-06
WSC1	Putative cell wall component	1.35	1.52E-08
ISW2	Ortholog of *S. cerevisiae* Isw2	1.35	1.37E-06
RIM9	Protein required for alkaline pH response	1.33	2.29E-08
HIP1	Amino acid permeases	1.33	8.59E-11
C3_01850W_A	Ortholog(s) have structural constituent of ribosome activity	1.32	3.94E-06
CWC22	Predicted spliceosome-associated protein	1.32	8.07E-10
C5_04050W_A	Plasma membrane-localized protein of unknown function	1.30	2.88E-06
C4_03410W_A	Ortholog(s) have structural constituent of ribosome activity	1.30	6.53E-10
MSW1	Protein similar to *S. cerevisiae* Msw1p	1.30	3.88E-10
C1_06350W_A	Protein of unknown function	1.27	4.81E-07
GIS2	Translational activator for mRNAs with internal ribosome entry sites	1.27	2.88E-06
PTC8	Predicted type 2C protein phosphatase	1.26	3.05E-06
CTA2	Putative transcription factor	1.25	6.22E-07
C2_03490C_A	Ortholog of *C. dubliniensis* CD36 : Cd36_18230	1.25	8.79E-08
MRPL10	Putative mitochondrial large subunit ribosomal protein	1.22	6.50E-06
SSU81	Predicted adaptor protein	1.21	1.37E-06

MNN23	Alpha-1,2-mannosyltransferase	1.21	4.86E-06
C1_14090W_A	Ortholog(s) have chaperone binding, unfolded protein binding activity	1.21	2.02E-07
C5_00250C_A	Protein with a predicted role in microtubule-related processes	1.19	2.61E-06
MIA40	Predicted component of the mitochondrial intermembrane space import machinery	1.18	1.24E-06
FLO8	Transcription factor	1.16	3.40E-06
FGR15	Putative transcription factor with zinc finger DNA-binding motif	1.16	8.13E-08
MIH1	Putative protein phosphatase of the PTP family (tyrosine-specific)	1.14	7.23E-06
C7_03000C_A	Ortholog(s) have structural constituent of ribosome activity	1.14	1.46E-07
WHI3	Putative RNA binding protein	1.13	6.19E-06
C1_14330W_A	Ortholog(s) have tRNA-5-taurinomethyluridine 2-sulfurtransferase activity	1.11	9.12E-06
ALS6	ALS family protein	1.10	1.42E-08
C2_07440C_A	Ortholog(s) have sterol esterase activity	1.10	9.29E-07
C1_05210C_A	Protein of unknown function	1.09	1.52E-06
C7_01910C_A	Similar to bacterial DnaJ	1.08	8.13E-08
ADA2	Zinc finger and homeodomain transcriptional coactivator	1.08	2.42E-06
YOR1	Protein similar to *S. cerevisiae* Yor1	1.03	2.98E-07
CR_03110W_A	Ortholog(s) have role in mitochondrial translation	1.02	1.63E-06
C4_00910C_A	Protein with similarity to mutator-like element (MULE) transposase	1.01	6.12E-08
CR_10420W_A	Protein with a mitochondrial distribution and morphology domain	0.99	1.99E-06
C1_13880C_A	C2H2 transcription factor	0.95	9.55E-06
C6_00390W_A	Ortholog(s) have serine-tRNA ligase activity	0.90	4.48E-07
C4_03780C_A	Putative mitochondrial membrane protein	0.84	6.06E-06
AFG3	Similar to *S. cerevisiae* Afg3p	0.81	2.26E-08
C2_00730C_A	Ortholog of *C. dubliniensis* CD36 : Cd36_15670	0.80	2.51E-11
SWI6	Putative component of the MBF and SBF transcription complexes	0.74	1.72E-07
WAR1	Zn(II)2Cys6 transcription factor	0.74	1.63E-06
PDR6	Putative pleiotropic drug resistance regulatory protein 6	0.67	3.90E-07
ZCF9	Putative Zn(II)2Cys6 transcription factor	-0.59	1.73E-06
C5_03940C_A	Putative multidrug resistance protein	-0.66	9.64E-07
C3_01510W_A	Ortholog(s) have phosphatidate phosphatase activity	-0.74	6.33E-06
VPS8	Putative vacuolar sorting protein	-0.76	5.73E-09
CR_01130W_A	Ortholog(s) have role in positive regulation of TORC1 signaling and Seh1-associated complex	-0.80	4.39E-06
SEC34	Ortholog(s) have protein transporter activity and role in CVT pathway	-0.80	7.00E-06
RLM1	Putative transcription factor	-0.81	2.97E-06
APL5	Ortholog of *S. cerevisiae* and *S. pombe* Apl5	-0.83	5.54E-06
CWH43	Putative sensor/transporter protein	-0.85	4.81E-06
PSD1	Phosphatidylserine decarboxylase	-0.86	2.91E-06
TAF60	Putative TFIID and SAGA complex subunit	-0.86	6.04E-06
PKH2	Putative serine/threonine protein kinase	-0.87	2.55E-06
C2_01490C_A	Putative RNA polymerase transcription factor TFIIH core component	-0.87	2.11E-06
POM152	Putative nuclear pore membrane glycoprotein	-0.91	7.60E-07
C5_04320C_A	RNI-like superfamily domain-containing protein	-0.92	5.20E-06
C1_12740W_A	Predicted cysteine proteinase domain	-0.95	5.88E-07
C5_01350W_A	Ortholog(s) have role in ER-dependent peroxisome organization	-0.96	2.51E-06
C3_02080W_A	Has domain(s) with predicted zinc ion binding activity	-0.96	5.65E-07

C1_04780C_A	Ortholog(s) have role in negative regulation of TORC1 signaling	-0.97	1.79E-11
RAD7	Protein similar to *S. cerevisiae* Rad7p	-0.98	1.35E-06
C3_02690C_A	Ortholog(s) have dolichol kinase activity	-0.98	5.02E-06
VPS36	ESCRT II protein sorting complex subunit	-0.98	1.99E-06
C4_01790W_A	Adapter protein for pexophagy and the cytoplasm-to-vacuole targeting (Cvt) pathway	-0.99	1.12E-07
TEL1	Ortholog(s) have protein serine/threonine kinase activity	-0.99	9.83E-11
SHE3	mRNA-binding protein	-0.99	1.09E-06
CR_06470W_A	Ortholog(s) have ubiquitin protein ligase activity	-1.00	6.12E-06
C5_01290C_A	Ortholog(s) have 2-octoprenyl-3-methyl-6-methoxy-1,4-benzoquinone hydroxylase activity	-1.00	3.21E-06
ORC3	Protein similar to *S. cerevisiae* Orc3p	-1.01	1.20E-08
CR_08550W_A	Ortholog(s) have fructose-2,6-bisphosphate 2-phosphatase activity	-1.02	2.80E-07
CAS4	RAM cell wall integrity signaling network protein	-1.02	9.78E-08
C4_00380W_A	Ortholog(s) have D-arabinose 1-dehydrogenase [NAD(P)+] activity	-1.02	1.72E-07
SMF3	Putative vacuolar iron transporter	-1.04	1.80E-07
C5_00750C_A	Predicted protein tyrosine phosphatase	-1.04	3.34E-07
CR_05890C_A	Ortholog(s) have acetyltransferase activator activity	-1.05	8.35E-08
C4_06740C_A	Ortholog(s) have protein binding, bridging activity	-1.06	8.51E-07
C3_07230W_A	Aminophospholipid translocase (flippase)	-1.06	2.88E-12
RTA2	Flippase	-1.06	9.25E-09
C7_04210C_A	Protein similar to Aspergillus CYSK O-acetylserine sulfhydrylase	-1.08	3.29E-06
HEM2	Putative porphobilinogen synthase	-1.08	2.42E-07
SOL3	Putative 6-phosphogluconolactonase	-1.09	8.75E-06
MKC1	MAP kinase	-1.09	2.28E-07
C4_05800C_A	Protein of unknown function	-1.10	3.99E-06
ERV46	Putative ER-derived vesicle protein	-1.10	8.60E-08
PGA63	Component COPII vesicle coat	-1.10	9.85E-07
C2_02020W_A	Ortholog(s) have Lys48-specific deubiquitinase activity	-1.11	1.12E-06
C1_04230W_A	Ortholog(s) have ubiquitin binding activity	-1.12	4.50E-07
C5_01090C_A	Planktonic growth-induced gene	-1.12	7.69E-06
C4_06020C_A	Ortholog(s) have DNA-directed DNA polymerase activity	-1.13	3.61E-10
CR_09340W_A	Ortholog(s) have ubiquitin conjugating enzyme activity	-1.13	2.68E-07
C1_01210W_A	Ortholog of *Candida tropicalis* MYA-3404 : CTRG_04422	-1.13	8.11E-07
SSN8	Ortholog of S. cerevisiae Ssn8	-1.14	8.23E-09
C5_01940W_A	Ortholog(s) have 2,5-diamino-6-ribitylamino-4(3H)-pyrimidinone 5'-phosphate deaminase activity	-1.15	2.62E-08
MUQ1	Putative choline phosphate cytidylyltransferase/phosphoethanolamine cytidylyltransferase	-1.15	9.25E-09
PMT1	Protein mannosyltransferase	-1.16	8.45E-06
SEN1	Putative helicase	-1.16	1.44E-07
C4_06130W_A	Ortholog(s) have tubulin binding activity	-1.16	1.23E-07
CR_08430W_A	Protein similar to *S. cerevisiae* Vac14p	-1.18	1.89E-11
C7_00140C_A	Ortholog(s) have cytosol, nucleus localization	-1.18	1.77E-10
CDC10	Septin	-1.18	7.05E-06
UBA4	Putative ubiquitin activating protein	-1.18	1.07E-08
C1_01300W_A	Putative thioredoxin	-1.18	1.58E-09

C1_03330C_A	Ortholog(s) have phosphatidylinositol-3,5-bisphosphate binding activity	-1.21	1.65E-15
ARO80	Zn(II)2Cys6 transcription factor	-1.22	1.75E-12
LSC1	Putative succinate-CoA ligase subunit	-1.23	1.16E-06
CYK3	Essential protein involved in cytokinesis	-1.23	1.46E-06
C1_02440C_A	Ortholog(s) have thiol-dependent ubiquitin-specific protease activity	-1.23	1.35E-06
C5_02050W_A	Ortholog(s) have role in Golgi to endosome transport, cell morphogenesis, cleistothecium development, conidium formation and endoplasmic reticulum organization	-1.23	5.38E-11
BET2	Putative Type II geranylgeranyltransferase beta subunit	-1.23	1.16E-08
C2_07010W_A	Predicted ER protein involved in ER-nucleus signaling	-1.23	2.10E-10
CR_04080C_A	Endoplasmic reticulum (ER) protein-translocation complex subunit	-1.24	4.96E-11
PFY1	Profilin; functional homolog of *S. cerevisiae* Pfy1	-1.24	2.43E-06
RET2	Delta subunit of the coatomer complex (COPI)	-1.25	1.88E-06
MYO2	Class V myosin	-1.25	7.31E-07
SEC7	Putative guanine nucleotide exchange factor (GEF)	-1.27	2.62E-11
C4_01280C_A	Ortholog(s) have RNA binding activity	-1.27	1.52E-08
COQ6	Ortholog(s) have oxidoreductase activity	-1.28	3.81E-12
VPS1	Dynamin-family GTPase-related protein	-1.28	1.19E-09
CCH1	Voltage-gated Ca2+ channel of the high affinity calcium uptake system	-1.29	2.60E-14
CHS7	Protein required for wild-type chitin synthase III activity	-1.29	6.28E-10
KTR4	Mannosyltransferase	-1.30	1.44E-07
IML2	Protein of unknown function	-1.30	8.23E-09
VMA4	H+ transporting ATPase E chain	-1.31	9.47E-06
TUB1	Alpha-tubulin	-1.32	1.16E-08
C1_01400C_A	Ortholog(s) have sphingosine-1-phosphate phosphatase activity	-1.33	2.00E-07
TIP120	Protein similar to human CAND1 (Cullin-Associated Nedd8-Dissociated) protein	-1.33	5.93E-07
C3_07770C_A	Ortholog(s) have microtubule binding, protein homodimerization activity	-1.33	1.64E-10
C3_06750W_A	Ortholog(s) have P-P-bond-hydrolysis-driven protein transmembrane transporter activity	-1.34	1.94E-07
C3_04460W_A	Ortholog(s) have role in positive regulation of TORC1 signaling	-1.35	8.86E-07
RNR1	Ribonucleotide reductase large subunit	-1.35	3.71E-06
STT3	Putative oligosaccharyltransferase complex component	-1.35	1.12E-06
MYO1	Component of actomyosin ring at neck of newly-emerged bud	-1.35	1.62E-13
PDS5	Putative protein with a predicted role in establishment and maintenance of sister chromatid condensation and cohesion	-1.36	1.59E-06
NPT1	Putative nicotinate phosphoribosyltransferase	-1.36	3.75E-13
RHO2	Ortholog(s) have GTPase activity	-1.37	1.44E-07
USO6	Putative vesicular transport protein	-1.37	2.59E-11
C6_00890W_A	Ortholog(s) have cytoplasm localization	-1.38	7.22E-10
DOA1	WD repeat protein	-1.38	1.34E-06
ERG7	2,3-epoxysqualene-lanosterol cyclase (lanosterol synthase)	-1.38	8.50E-07
PGA53	GPI-anchored cell surface protein of unknown function	-1.40	4.71E-06
SEC13	Putative protein transport factor	-1.40	2.11E-08
C6_02680W_A	Ortholog(s) have SNAP receptor activity	-1.40	8.34E-07
C1_02770W_A	Protein of unknown function	-1.40	2.43E-10
MPT5	Putative RNA-binding protein	-1.40	1.11E-12

C1_07050C_A	Ortholog(s) have ATP-dependent 5'-3' DNA helicase activity	-1.41	4.26E-09
C1_09220W_A	Predicted MFS family membrane transporter	-1.41	1.90E-07
C2_05720C_A	Putative oxysterol binding protein family	-1.41	2.73E-16
DYN1	Dynein heavy chain	-1.42	8.45E-16
C3_06710W_A	Protein of unknown function	-1.43	7.44E-17
SRB1	Essential GDP-mannose pyrophosphorylase	-1.43	7.34E-06
SMP2	Putative Mg2+-dependent phosphatidate phosphatase	-1.44	6.93E-07
C1_06700W_A	SH3-domain protein	-1.45	1.96E-06
C6_02490C_A	Protein of unknown function	-1.46	1.01E-07
C1_07260C_A	Ortholog(s) have DNA replication origin binding, single-stranded DNA binding activity	-1.46	1.23E-21
RAD50	Putative DNA double-strand break repair factor	-1.46	2.94E-09
CHS5	Putative chitin biosynthesis protein	-1.46	2.47E-08
C1_03100W_A	Putative lipid-binding protein	-1.46	7.05E-08
SCT2	Putative glycerol-3-phosphate acyltransferase	-1.47	3.90E-09
BFR1	Protein involved in the maintenance of normal ploidy;	-1.47	2.38E-08
UGP1	UTP-glucose-1-phosphaturidyl transferase	-1.47	8.94E-06
YKE2	Possible heterohexameric Gim/prefoldin protein complex subunit	-1.49	9.55E-06
PTP3	Putative protein tyrosine phosphatase	-1.49	3.43E-11
LIG4	DNA ligase	-1.50	4.13E-12
C1_14170W_A	Ortholog(s) have ubiquitin-protein transferase activity	-1.50	6.89E-12
MON2	Peripheral membrane protein	-1.51	2.04E-08
PMM1	Phosphomannomutase	-1.52	2.38E-08
BMT3	Beta-mannosyltransferase	-1.52	2.48E-09
CDC3	Septin	-1.53	4.15E-12
C2_09480W_A	Ortholog(s) have DNA-directed DNA polymerase activity	-1.54	1.25E-10
C6_02650C_A	Predicted membrane transporter	-1.55	7.14E-09
C6_01950C_A	Protein of unknown function	-1.56	4.60E-07
PHM7	Putative transporter	-1.56	6.42E-06
C1_13500C_A	H/ACA box small nucleolar RNA (snoRNA)	-1.57	1.40E-06
C2_00230W_A	Ortholog(s) have arginyltransferase activity	-1.57	1.57E-12
PMT2	Protein mannosyltransferase (PMT) with roles in hyphal growth and drug sensitivity	-1.57	1.32E-08
GTT11	Glutathione S-transferase	-1.58	3.08E-10
CR_02300C_A	Protein with a Vps9 vacuolar protein sorting protein domain	-1.58	5.19E-15
C3_03370C_A	Protein of unknown function	-1.58	6.19E-09
C5_04470C_A	Predicted RNA binding protein	-1.58	9.03E-06
NCP1	NADPH-cytochrome P450 reductase	-1.59	3.71E-14
BTS1	Putative geranylgeranyl diphosphate synthase	-1.60	4.11E-13
REV3	Ortholog(s) have DNA-directed DNA polymerase activity	-1.60	2.61E-09
EXG2	GPI-anchored cell wall protein	-1.61	9.76E-08
C7_02850W_A	Ortholog of *Candida albicans* WO-1 : CAWG_05627	-1.63	9.53E-11
SET3	NAD-dependent histone deacetylase	-1.64	8.22E-14
CLN3	G1 cyclin	-1.65	2.02E-06
CYB5	Cytochrome b(5)	-1.66	6.50E-07
C1_11120C_A	Protein similar to *S. cerevisiae* Gvp36p	-1.67	2.26E-18
PMI1	Phosphomannose isomerase	-1.67	6.30E-06
SVF1	Putative survival factor	-1.68	2.68E-13

C5_04350C_A	Ortholog(s) have cell division site, cell tip, cytosol, nucleus localization	-1.68	3.51E-14
PMT4	Protein mannosyltransferase	-1.69	5.51E-06
SEC23	Putative GTPase-activating protein	-1.69	3.05E-07
C1_02780W_A	Predicted dienelactone hydrolase domain	-1.69	9.75E-08
FAS2	Alpha subunit of fatty-acid synthase	-1.69	1.66E-07
RDI1	Putative rho GDP dissociation inhibitor	-1.72	3.90E-06
C4_06990W_A	Possible mannosyltransferase	-1.73	1.58E-16
TPK2	cAMP-dependent protein kinase catalytic subunit	-1.75	8.64E-09
C2_08620W_A	Protein of unknown function	-1.76	3.24E-10
C3_04740C_A	Ortholog(s) have DNA topoisomerase type I activity	-1.76	2.64E-07
CR_09010C_A	Putative cystathionine gamma-synthase	-1.77	8.86E-07
CR_04870C_A	Trimethylaminobutyraldehyde dehydrogenase	-1.77	5.45E-06
AGO1	Putative Argonaute protein involved in RNA silencing	-1.78	6.15E-06
ECM33	GPI-anchored cell wall protein	-1.78	1.15E-10
CDC12	Septin	-1.78	1.40E-11
CR_09330C_A	Protein with a Staphylococcal nuclease domain	-1.80	3.71E-10
C5_02520W_A	Protein of unknown function	-1.81	5.89E-11
ENO1	Enolase	-1.81	1.73E-06
SEC61	ER protein-translocation complex subunit	-1.81	2.61E-06
CR_00290W_A	Ortholog of S. cerevisiae Rts3	-1.82	4.72E-14
C2_02220C_A	Protein of unknown function	-1.82	2.61E-06
ERG2	C-8 sterol isomerase	-1.82	1.14E-08
C1_09130W_A	Protein of unknown function	-1.82	7.74E-09
STE13	Putative pheromone-processing dipeptidyl aminopeptidase	-1.84	1.24E-14
PSD2	Ortholog(s) have phosphatidylserine decarboxylase activity	-1.84	7.61E-20
NIP100	p150 subunit of dynactin	-1.85	2.42E-08
ACB1	Protein similar to a region of acyl-coenzyme-A-binding protein	-1.89	2.24E-06
DPM1	Dolichol-phosphate mannose synthase catalytic subunit	-1.90	1.58E-10
YVC1	Putative vacuolar cation channel shock	-1.90	7.81E-12
FBA1	Fructose-bisphosphate aldolase	-1.91	5.04E-11
C1_11250W_A	Microtubule-binding protein of the cortical microtubule	-1.91	5.84E-16
C3_06860C_A	Putative xylose and arabinose reductase	-1.91	3.56E-06
ERG3	C-5 sterol desaturase	-1.94	5.82E-06
C4_05250W_A	Putative ubiquitin-protein ligase	-1.94	2.43E-10
INN1	Protein with similarity to *S. cerevisiae* Inn1p	-1.94	2.19E-27
C5_02850W_A	H/ACA box small nucleolar RNA (snoRNA)	-1.95	5.87E-08
MTS1	Sphingolipid C9-methyltransferase	-1.95	5.19E-13
C5_01070C_A	Protein of unknown function	-1.96	8.72E-06
C2_09810C_A	Protein with predicted peptidase domains	-1.96	8.75E-06
FAV2	Adhesin-like protein	-1.99	1.12E-06
NAT4	Putative histone acetyltransferase	-2.00	1.49E-11
C3_04420W_A	Ortholog of *S. cerevisiae* Hul4	-2.01	5.36E-10
SSO2	Plasma membrane t-SNARE	-2.02	2.14E-31
C5_03490C_A	Stationary phase enriched protein	-2.03	1.75E-20
CDC19	Pyruvate kinase at yeast cell surface	-2.05	1.11E-09
TPM2	Putative tropomyosin isoform 2	-2.08	1.57E-10
CR_09070C_A	Protein of unknown function	-2.09	1.64E-12

ERG9	Putative farnesyl-diphosphate farnesyl transferase (squalene synthase)	-2.11	3.41E-06
ERG251	C-4 sterol methyl oxidase	-2.11	2.28E-06
SLD1	Sphingolipid delta-8 desaturase	-2.12	1.74E-16
CR_04120C_A	Ortholog(s) have DNA-dependent ATPase activity	-2.13	7.35E-12
C1_11790W_A	Ortholog(s) have thymidylate kinase activity	-2.16	1.62E-13
GAL4	Zn(II)2Cys6 transcription factor	-2.17	9.77E-12
C1_11730W_A	Protein with SEL-1 like protein domain	-2.18	4.07E-19
ERG27	3-Keto sterol reductase	-2.18	1.46E-11
DAP1	Similar to mammalian membrane-associated progesterone receptors	-2.18	5.65E-23
ERG11	Lanosterol 14-alpha-demethylase	-2.20	1.79E-08
MSC7	*S. cerevisiae* ortholog Mcs7	-2.20	1.08E-50
PST3	Flavodoxin-like protein	-2.20	1.59E-07
CRL1	Predicted GTPase of RHO family	-2.20	2.57E-25
C7_01390W_A	Protein of unknown function	-2.20	1.18E-07
C2_07220W_A	Putative 20S proteasome assembly protein	-2.21	3.12E-12
CEK1	ERK-family protein kinase	-2.22	8.30E-16
C1_04470C_A	Protein of unknown function	-2.23	7.36E-13
C4_01860C_A	Possible Golgi membrane protein	-2.23	5.01E-09
C1_09980C_A	Ortholog(s) have acylglycerol lipase activity	-2.25	2.20E-08
MNN12	Predicted alpha-1,3-mannosyltransferase activity	-2.25	1.85E-09
C1_11720W_A	Ortholog of *Candida albicans* WO-1 : CAWG_00265	-2.26	4.14E-08
LAP41	Putative aminopeptidase yscI precursor	-2.28	1.34E-10
CHS8	Chitin synthase required for synthesis of long-chitin fibrils	-2.28	7.44E-17
MLC1	Microtubule-dependent localized protein	-2.29	7.04E-18
GND1	6-phosphogluconate dehydrogenase	-2.31	5.32E-07
PGA54	GPI-anchored protein	-2.31	1.27E-09
C1_04580C_A	Ortholog of Rmd6 involved in *S. cerevisiae* sporulation	-2.33	1.30E-17
CR_09040W_A	Protein of unknown function	-2.35	4.48E-09
ZWF1	Glucose-6-phosphate dehydrogenase	-2.36	2.54E-08
ECM331	GPI-anchored protein	-2.40	6.90E-08
C4_00860C_A	Protein of unknown function	-2.41	2.85E-11
CR_04770C_A	Ortholog of *C. dubliniensis* CD36 : Cd36_30140	-2.41	6.35E-18
ERG5	Putative C-22 sterol desaturase	-2.46	5.02E-12
GFA1	Glucosamine-6-phosphate synthase	-2.50	7.47E-16
ERG10	Acetyl-CoA acetyltransferase	-2.52	1.16E-11
BMT7	Beta-mannosyltransferase	-2.53	2.18E-09
ERG26	C-3 sterol dehydrogenase	-2.56	4.64E-18
UPC2	Zn2-Cys6 transcript factor	-2.57	1.42E-41
C1_13490C_A	Ortholog(s) have role in cellular calcium ion homeostasis	-2.59	9.62E-29
C1_11260C_A	Putative adhesin-like protein	-2.63	3.80E-14
CHS2	Chitin synthase	-2.64	2.54E-47
ERG1	Squalene epoxidase	-2.67	1.36E-30
PGA6	GPI-anchored cell wall adhesin-like protein	-2.73	9.82E-09
PGA31	Cell wall protein	-2.75	9.31E-06
C7_02280W_A	Ortholog of *C. parapsilosis* CDC317 : CPAR2_808370	-2.76	7.50E-07
CHO1	Putative phosphatidylserine synthase	-2.78	3.25E-15
C6_03260W_A	Putative ribonuclease H1	-2.82	1.15E-17

ERG24	C-14 sterol reductase	-2.92	5.37E-24
ERG4	Protein similar to sterol C-24 reductase	-3.12	1.38E-11
IPK2	Putative inositol polyphosphate multikinase	-3.17	1.60E-33
C2_02910W_A	Protein of unknown function	-3.18	5.17E-13
C7_02260W_A	Ortholog of *C. parapsilosis* CDC317: CPAR2_808350	-3.18	4.33E-08
RBT4	Pry family protein	-3.24	2.14E-12
DAG7	Secretory protein;	-3.26	4.72E-10
LDG3	Putative LDG family protein	-3.30	9.04E-08
C7_03310W_A	Protein of unknown function	-3.33	5.67E-09
C6_02100W_A	Secreted potein	-3.39	1.10E-09
C3_06660C_A	Ortholog of *C. dubliniensis* CD36: Cd36_86630	-3.50	1.92E-07
HWP1	Hyphal cell wall protein	-3.62	8.94E-08
C1_04590W_A	Protein of unknown function	-3.65	3.47E-27
DDR48	Immunogenic stress-associated protein	-3.78	2.14E-14
PGA45	Putative GPI-anchored cell wall protein	-3.82	2.94E-11
ERG6	Delta (24)-sterol C-methyltransferase	-3.82	3.15E-27
RBR1	Glycosylphosphatidylinositol (GPI)-anchored cell wall protein	-3.85	6.87E-14
C2_10160W_A	Secreted protein	-3.92	2.18E-22
CRH11	GPI-anchored cell wall transglycosylase	-4.33	2.12E-26
PGA23	Putative GPI-anchored protein of unknown function	-4.67	9.84E-25
MRV8	Protein of unknown function	-4.82	9.56E-18
PGA13	GPI-anchored cell wall protein	-5.42	6.17E-38
C3_01540W_A	Plasma-membrane-localized protein	-6.41	4.64E-40

Supplementary Table S7: Gene expression data; Significantly upregulated/downregulated genes in *C. albicans* treated with C12AHL+fluconazole compared to fluconazole only treated samples. The genes that are known to be associated with antimicrobial sensitivity/resistance are shaded in light blue (adjusted *p-value* < $1e^{-5}$).

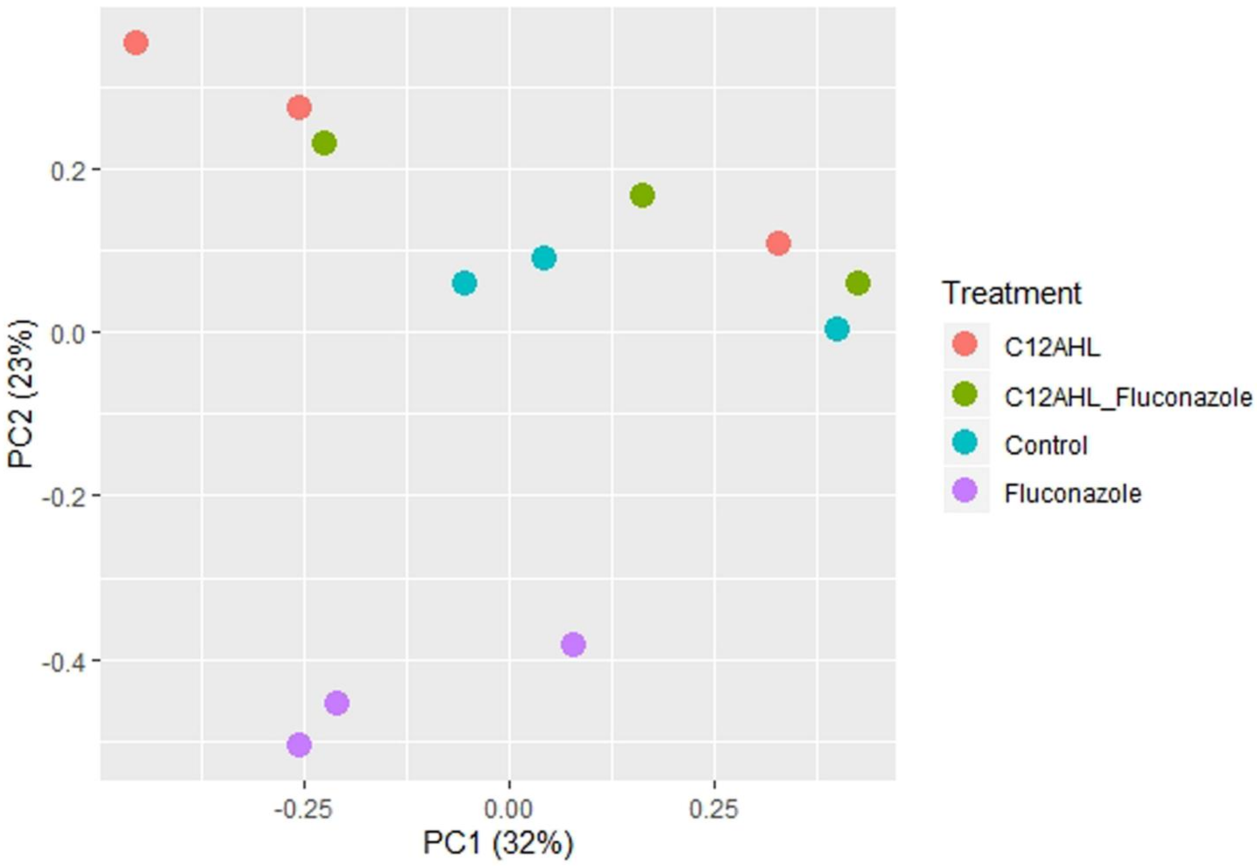

Supplementary Figure S8: Principle component analysis of *C. albicans* gene expression. The gene expression profiles for the control, C12AHL, C12AHL+fluconazole, and fluconazole only treatments were statistically compared using a principle component analysis to identify variability in gene expression between treatments. Each colour represents a different treatment as indicated in the right-side panel, and each circle represents the gene expression for one of the replicates per treatment.

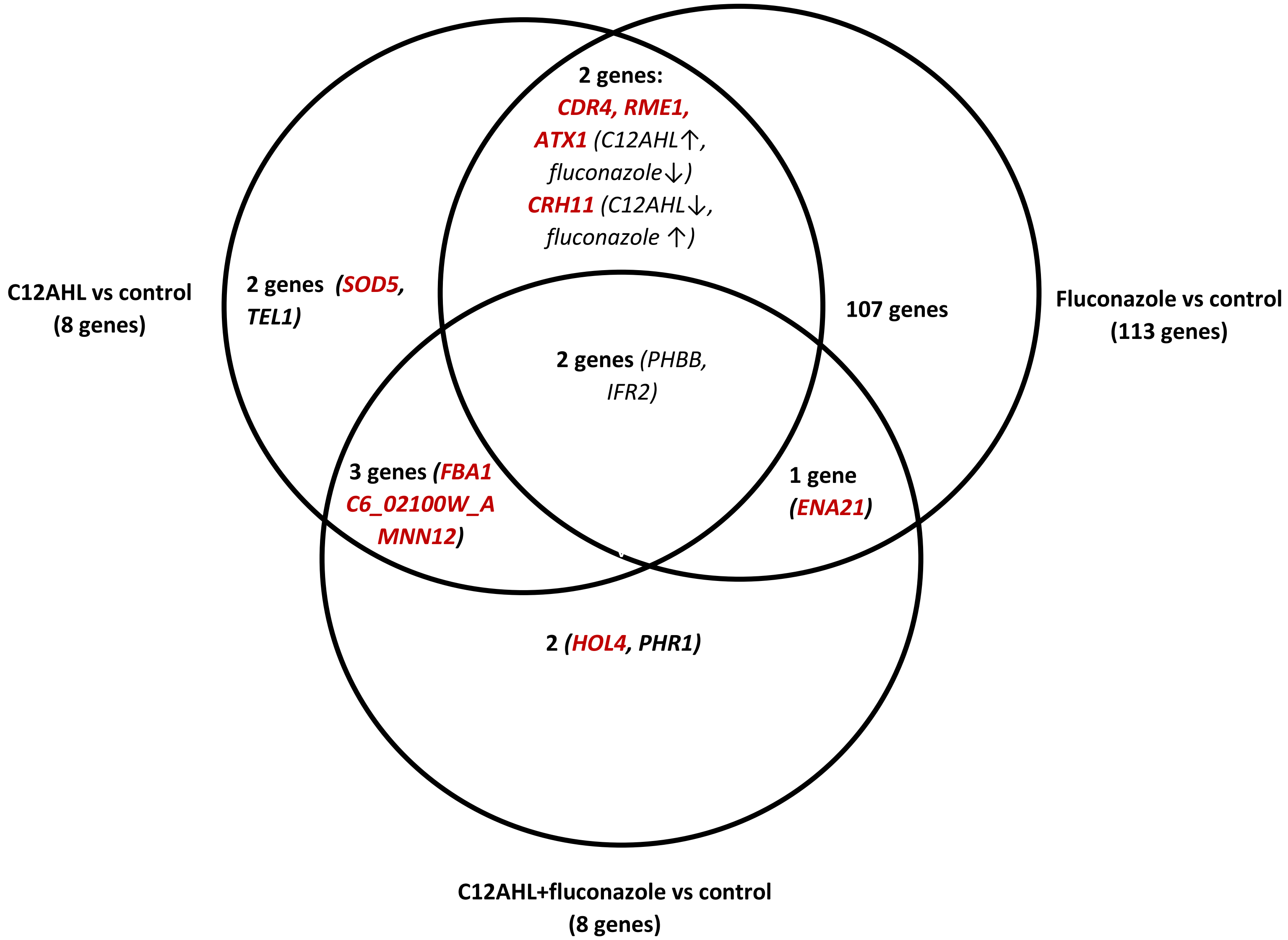

Supplementary Figure S9: Comparisons among significantly downregulated genes in *C. albicans* exposed to fluconazole, C12AHL or C12AHL+fluconazole. Genes that are highlighted in red are known to be associated with antifungal resistance (adjusted p-value < $1e^{-5}$). Genes that were significantly downregulated between treatments are shown in Supplementary Tables 2-6.

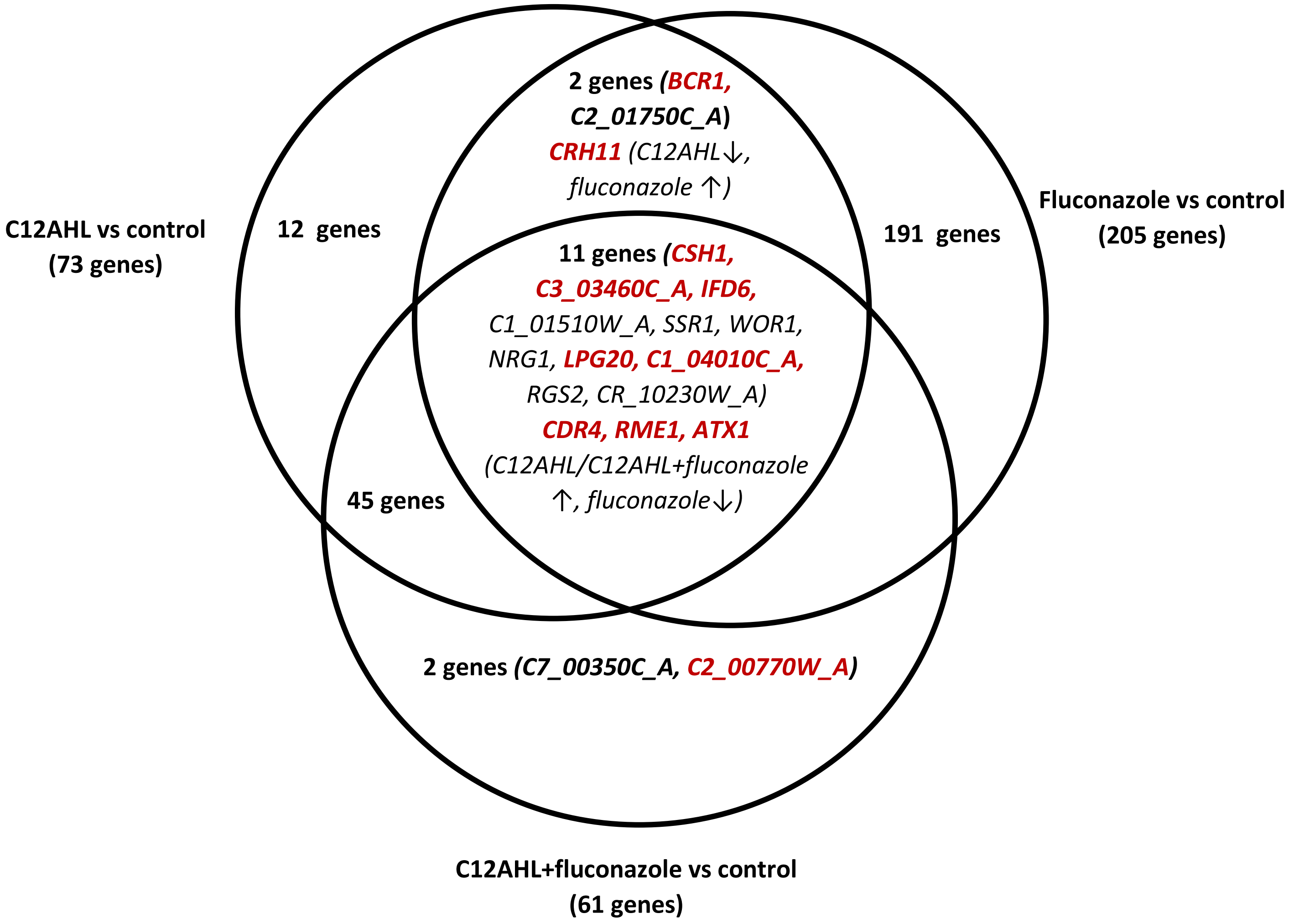

Supplementary Figure S10: Comparisons among significantly upregulated genes in *C. albicans* exposed to fluconazole, C12AHL or C12AHL+fluconazole. Genes that are highlighted in red are known to be associated with antifungal resistance (adjusted p-value < $1e^{-5}$). Genes that were significantly upregulated between treatments are shown in Supplementary Tables 2-6.

Biological function	Protein (accession No.)	Protein family	Gene	Notes	Reference
Antifungal resistance	Gcy1p (gi|68472117)	Glycerol 2-dehydrogenase (NADP(+))	*GCY1*	mutation confers hypersensitivity to toxic ergosterol analogue	[1,2]
	Lsc1p (gi|68475125)***	Succinate-CoA ligase (putative)	*LSC1*	Induced by fluconazole	[3]
Respiration	Cox4p (gi|68466651)	Cytochrome c oxidase subunit IV	*COX4*		[4,5]
	Lpd1p (gi|68487498)***	Dihydrolipoyl dehydrogenase (putative)	*LPD1*		[6]
	Pda1p (gi|68481122)***	Pyruvate dehydrogenase (acetyl-transferring) subunit E1 alpha	*PDA1*	Induced by fluconazole	[3,6]
	Atp1p (gi|238879075)	ATP synthase alpha unit	*ATP1*	Induced by ciclopirox, ketoconazole, flucytosine	[7]
Oxidative stress	Sod1p (gi|6094316)	Superoxide dismutase	*SOD1*		[8]
	Cyp5p (gi|68479834)	Peptidyl-prolyl cis-trans isomerase	*CYP5*		[9]
	Pst1p (gi|68491679)	NAD(P)H dehydrogenase (flavodoxin-like protein)	*PST3*		[10,11]
	Mxr1p (gi|68472567)***	Peptide-methionine -S-oxide reductase (putative)	*MXR1*	Induced by flucytosine	[7]
Apoptosis	Cpr3p (gi|68466799)	Peptidyl-prolyl cis-trans isomerase (putative)	*CPR3*		[12]
Peptide synthesis/Protein folding	Cr_04650wp_a (gi|23887992)	Protein involved in microtubule morphogenesis	*CR_04650W_A (RBL2)*		[13]
	Mmd1p (Hmf1p)** (gi|238878875)	Deaminase	*MMD1 (HMF1)***		[9,13]
Nucleic acid synthesis	Dut1p (gi|68475057)***	Bifunctional dITP/dUTP diphosphatase	*DUT1*		[14]
	Hnt1p (gi|68484224)***	Adenosine 5'-monophosphoramidase	*HNT1*		[6]
Cellular metabolism	Ach1p (gi|68482646)***		*ACH1*	Induced by ketoconazole	[7]
Unknown	Hypothetical (gi|68487695)[x]	Unknown	*CaO19.9673*[x]		

*Nomenclature conflict, ** Orthologs, *** Obsolete record in NCBI, thus matched for an identical protein, [x] Obsolete record in NCBI

Supplementary Table S11: Protein expression data; Under-expressed proteins in C12AHL+fluconazole treated *C. albicans* samples compared to fluconazole only treated samples

1 Askew, C. *et al.* Transcriptional regulation of carbohydrate metabolism in the human pathogen *Candida albicans*. *PLoS Pathog* **5**, e1000612, doi:10.1371/journal.ppat.1000612 (2009).

2 Xu, D. *et al.* Genome-wide fitness test and mechanism-of-action studies of inhibitory compounds in *Candida albicans*. *PLoS Pathog* **3**, e92, doi:10.1371/journal.ppat.0030092 (2007).

3 Copping, V. M. *et al.* Exposure of *Candida albicans* to antifungal agents affects expression of *SAP2* and *SAP9* secreted proteinase genes. *J Antimicrob Chemother* **55**, 645-654, doi:10.1093/jac/dki088 (2005).

4 Cabezon, V., Llama-Palacios, A., Nombela, C., Monteoliva, L. & Gil, C. Analysis of *Candida albicans* plasma membrane proteome. *Proteomics* **9**, 4770-4786, doi:10.1002/pmic.200800988 (2009).

5 Mitrovich, Q. M., Tuch, B. B., Guthrie, C. & Johnson, A. D. Computational and experimental approaches double the number of known introns in the pathogenic yeast *Candida albicans*. *Genome Res* **17**, 492-502, doi:10.1101/gr.6111907 (2007).

6 Kusch, H. *et al.* A proteomic view of *Candida albicans* yeast cell metabolism in exponential and stationary growth phases. *Int J Med Microbiol* **298**, 291-318, doi:10.1016/j.ijmm.2007.03.020 (2008).

7 Liu, T. T. *et al.* Genome-wide expression profiling of the response to azole, polyene, echinocandin, and pyrimidine antifungal agents in *Candida albicans*. *Antimicrob Agents Chemother* **49**, 2226-2236, doi:10.1128/AAC.49.6.2226-2236.2005 (2005).

8 Hwang, C. S. *et al.* Copper- and zinc-containing superoxide dismutase and its gene from *Candida albicans*. *Biochim Biophys Acta* **1427**, 245-255 (1999).

9 Fernandez-Arenas, E. *et al.* Integrated proteomics and genomics strategies bring new insight into *Candida albicans* response upon macrophage interaction. *Mol Cell Proteomics* **6**, 460-478, doi:10.1074/mcp.M600210-MCP200 (2007).

10 Li, L., Naseem, S., Sharma, S. & Konopka, J. B. Flavodoxin-Like Proteins Protect *Candida albicans* from Oxidative Stress and Promote Virulence. *PLoS Pathog* **11**, e1005147, doi:10.1371/journal.ppat.1005147 (2015).

11 Cowen, L. E. *et al.* Population genomics of drug resistance in *Candida albicans*. *Proc Natl Acad Sci U S A* **99**, 9284-9289, doi:10.1073/pnas.102291099 (2002).

12 Hirakawa, M. P. *et al.* Genetic and phenotypic intra-species variation in *Candida albicans*. *Genome Res* **25**, 413-425, doi:10.1101/gr.174623.114 (2015).

13 Nobile, C. J. *et al.* A recently evolved transcriptional network controls biofilm development in *Candida albicans*. *Cell* **148**, 126-138, doi:10.1016/j.cell.2011.10.048 (2012).

14 McIntosh, E. M., Looser, J., Haynes, R. H. & Pearlman, R. E. MluI site-dependent transcriptional regulation of the *Candida albicans* dUTPase gene. *Curr Genet* **26**, 415-421 (1994).

Biological function	Protein (Accession No.)	Protein family	Gene	Notes	Reference
Respiration	Gpm1p (gi|68469783)***	Phosphoglycerate mutase	*GPM1*	Induced by fluconazole	[1]
	Cit1p (gi|238879810)	ATP citrate synthase	*CIT1*		[2]
	Pck1p (gi|68474596)	Phosphoenolpyruvate carboxykinase (ATP)	*PCK1*	Induced by fluconazole	[1]
Oxidative stress	Hsp21p (gi|68469633)***	Unknown	*HSP21*	Downregulated by fluconazole	[1,3]
Apoptosis	Tdh3p (gi|68472227)	Glyceraldehyde-3-phosphate dehydrogenase (NAD+) (phosphorylating)	*TDH3/TDH1/GAP1**		[4]
Peptide synthesis/Protein folding	Lap3p (gi|68477033)***	Aminopeptidase (bleomycin hydrolase)	*LAP3*		[2]
Fermentation	Adh4p (gi|68471589)***	Predicated 3-hydroxyacyl-CoA dehydrogenase	*ADH4*	upregulated in azole-resistant strain overexpressing *MDR1*/ in fluconazole resistance strains	[5,6]

*Nomenclature conflict, ** Orthologs, *** Obsolete record in NCBI, thus matched for an identical protein, ^{x} Obsolete record in NCBI

Supplementary Table S12: Protein expression data: Overexpressed proteins when treated with C12AHL+fluconazole + *C. albicans* compared to fluconazole only treated samples

1 Copping, V. M. *et al.* Exposure of *Candida albicans* to antifungal agents affects expression of *SAP2* and *SAP9* secreted proteinase genes. *J Antimicrob Chemother* **55**, 645-654, doi:10.1093/jac/dki088 (2005).

2 Lan, C. Y. *et al.* Regulatory networks affected by iron availability in *Candida albicans*. *Mol Microbiol* **53**, 1451-1469, doi:10.1111/j.1365-2958.2004.04214.x (2004).

3 Mayer, F. L. *et al.* Small but crucial: the novel small heat shock protein Hsp21 mediates stress adaptation and virulence in *Candida albicans*. *PLoS One* **7**, e38584, doi:10.1371/journal.pone.0038584 (2012).

4 Delgado, M. L., Gil, M. L. & Gozalbo, D. Starvation and temperature upshift cause an increase in the enzymatically active cell wall-associated glyceraldehyde-3-phosphate dehydrogenase protein in yeast. *FEMS Yeast Res* **4**, 297-303, doi:10.1016/S1567-1356(03)00159-4 (2003).

5 Karababa, M., Coste, A. T., Rognon, B., Bille, J. & Sanglard, D. Comparison of gene expression profiles of *Candida albicans* azole-resistant clinical isolates and laboratory strains exposed to drugs inducing multidrug transporters. *Antimicrob Agents Chemother* **48**, 3064-3079, doi:10.1128/AAC.48.8.3064-3079.2004 (2004).

6 Cowen, L. E. *et al.* Population genomics of drug resistance in *Candida albicans*. *Proc Natl Acad Sci U S A* **99**, 9284-9289, doi:10.1073/pnas.102291099 (2002).

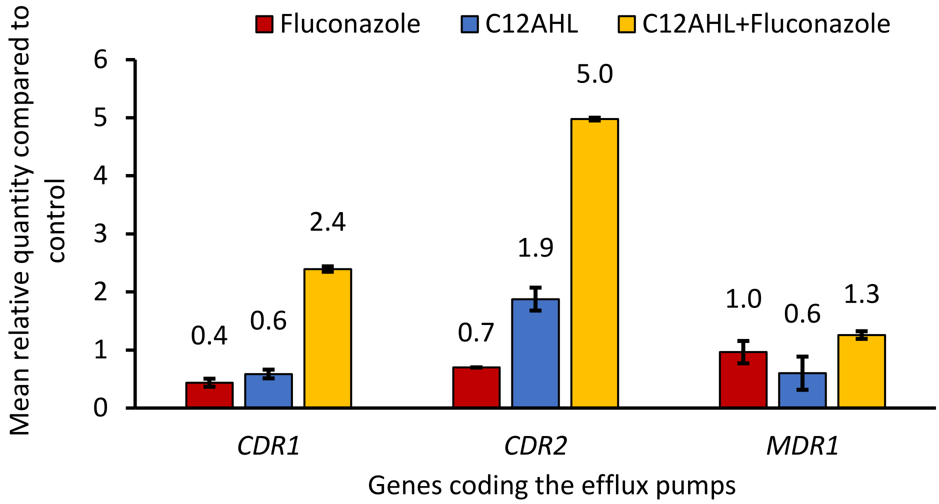

Supplementary Figure S13: Differential expression of *CDR1, CDR2* and *MDR1* to C12AHL, fluconazole or their combination. Real-time quantitative PCR assay data. Note the significant upregulation of *C. albicans CDR1* and *CDR2* when exposed to C12AHL+fluconazole and *CDR2* when exposed to C12AHL compared to solvent controls (P<0.05).

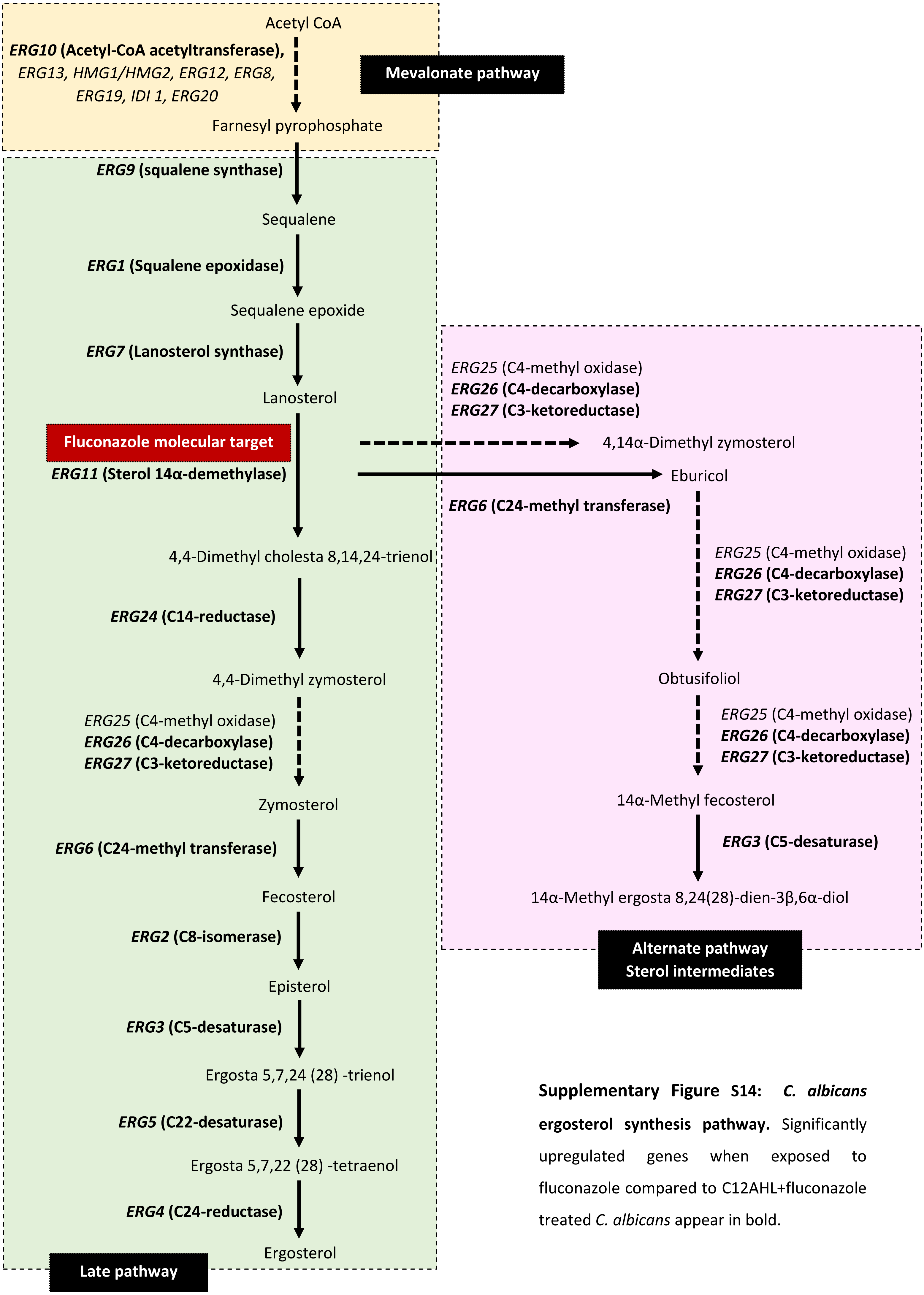

Supplementary Figure S14: *C. albicans* ergosterol synthesis pathway. Significantly upregulated genes when exposed to fluconazole compared to C12AHL+fluconazole treated *C. albicans* appear in bold.

C. albicans	Strain name	Genotype	Reference
**Wild type**	CAF2-1	*Δura3*::*imm434/URA3*	[1]
Δcdr1	DSY448	*Δcdr1*::*hisG-URA3-hisG/Δcdr1*::*hisG*	[1]
Δmdr1	DSY465	*Δmdr1*::*hisG-URA3-hisG/Δmdr1*::*hisG*	[1,2]
Δcdr1 Δcdr2	DSY654	*Δcdr1::hisG/Δcdr1::hisG*<br>*Δcdr2::hisG-URA3-hisG/Δcdr2::hisG*	[3]
Δcdr1 Δcdr2 Δmdr1	DSY1050	*Δcdr1*::*hisG/Δcdr1*::*hisG Δcdr2*::*hisG/Δcdr2*::*hisG*<br>*Δmdr1*::*hisG-URA3-hisG/Δmdr1*::*hisG*	[2]
Δerg3	DSY1751	*erg3AΔ::hisG/erg3BΔ::hisG-URA3-hisG*<br>*erg11Δ::hisG/ERG11*	[4]
Δerg11 Δerg3	DSY1764	*erg3AΔ::hisG/erg3BΔ::hisG*<br>*erg11Δ::hisG/erg11Δ::hisG-URA3-hisG*	[4]
Δerg11	DSY1769	*erg11Δ::hisG-URA3-hisG erg11Δ::hisG-URA3-hisG*	[4]

Supplementary Table S15: *Candida albicans* strains used in this study

1 Sanglard, D., Ischer, F., Monod, M. & Bille, J. Susceptibilities of *Candida albicans* multidrug transporter mutants to various antifungal agents and other metabolic inhibitors. *Antimicrob Agents Chemother* **40**, 2300-2305 (1996).

2 Mukherjee, P. K., Chandra, J., Kuhn, D. M. & Ghannoum, M. A. Mechanism of fluconazole resistance in *Candida albicans* biofilms: phase-specific role of efflux pumps and membrane sterols. *Infect Immun* **71**, 4333-4340 (2003).

3 Sanglard, D., Ischer, F., Monod, M. & Bille, J. Cloning of *Candida albicans* genes conferring resistance to azole antifungal agents: characterization of *CDR2*, a new multidrug ABC transporter gene. *Microbiology* **143 (Pt 2)**, 405-416, doi:10.1099/00221287-143-2-405 (1997).

4 Sanglard, D., Ischer, F., Parkinson, T., Falconer, D. & Bille, J. *Candida albicans* mutations in the ergosterol biosynthetic pathway and resistance to several antifungal agents. *Antimicrob Agents Chemother* **47**, 2404-2412, doi:10.1128/aac.47.8.2404-2412.2003 (2003).

C. albicans gene	Primer	Sequence (5′–3′)
CDR1	Forward	GCATATGGTGTTGCTAATGATTCTG
	Reverse	AAACCTTCTGTGGCTAATTTCCAA
CDR2	Forward	ACGGCAACATTAGTCATGAACAA
	Reverse	AGTTTTTCACCCAGTATTTGGCATT
MDR1	Forward	GCTAGTGTTGCTGATGTGGTTAAATT
	Reverse	CAAACAGCACCCAAACTCCAA
***EFB1* (House Keeping)**	Forward	TGTGAAAATCGGATCAATGCA
	Reverse	CATGGCCAATGCTCTTTCAA

Supplementary Table S16: The primers used for the Real-time quantitative PCR in this study
